# A realist review of factors critical for the implementation of eHealth in chronic disease management

**DOI:** 10.1186/s12913-025-12361-0

**Published:** 2025-04-02

**Authors:** Nida Shahid, Gillian Parker, Joanna M. Bielecki, Valeria Rac, Whitney Berta

**Affiliations:** 1 Institute of Health Policy, Management and Evaluation, Dalla Lana School of Public Health, University of Toronto, Toronto, ON Canada; 2 Program for Health System and Technology Evaluation, Ted Rogers Centre for Heart Research at Peter Munk Cardiac Centre, Toronto General Hospital Research Institute, University Health Network, Toronto, ON Canada

**Keywords:** Realist review, Health services research, Chronic disease management, eHealth, Telemedicine, Heart failure, Chronic kidney disease, Chronic obstructive pulmonary disease, Diabetes

## Abstract

**Background:**

In Canada, chronic disease is responsible for 88% of deaths and $120 billion in cost each year. With 44% of Canadian adults living with at least one chronic condition, only 66% receive necessary care. Ehealth interventions are instrumental in chronic disease management (CDM), especially since the pandemic, as they provide accessible, cost-effective solutions for self-management. Despite its promise and accelerated use, its implementation remains challenging. This paper reports on a realist review of critical factors for the implementation of eHealth interventions relevant to conditions such as heart failure, chronic obstructive pulmonary disease, chronic kidney disease, and diabetes. The findings are presented in terms of context, mechanisms, and outcomes.

**Methods:**

A realist review of the primary literature was conducted by searching five databases: Medline, Embase, Cochrane, CINALH and PsycInfo. The initial search was run for a date spanning from the databases’ inception to September 2018 and subsequently updated to dates spanning from October 2018 to May 2022. A systematic and iterative approach to data extraction was used. Thematic analysis was used to identify context-mechanism-outcome (CMO) configurations.

**Results:**

Among the 13,209 citations retrieved, 64 articles were included. This paper reports the top ten configurations found to facilitate or hinder eHealth implementation. Key themes related to context, such as team-based care, and action, including program use, perceived usefulness and motivation, are reported.

**Conclusions:**

This study explores the role of context, mechanisms, and outcomes in ehealth implementation, highlighting the nonlinear relationships between these factors. Future implications include empirical testing CMOs as middle-range theories in real-world settings to determine causality.

**Trial registration:**

The review protocol was registered with PROSPERO (CRD42020208275) on 1 October 2020.

**Supplementary Information:**

The online version contains supplementary material available at 10.1186/s12913-025-12361-0.

## Background

Chronic disease is responsible for 88% of all deaths in Canada and over $122.7^1^ billion in health care costs [1, 2]. Forty-four percent of Canadian adults live with one or more chronic conditions, such as heart failure (HF), diabetes, chronic obstructive pulmonary disease (COPD), and chronic kidney disease (CKD). The main types of chronic diseases in Canada are heart disease (e.g. heart failure), cancers, respiratory conditions (e.g., chronic obstructive pulmonary disease), and diabetes, which can lead to kidney disease [3]. Chronic kidney disease (CKD), affecting 8%−6% of people globally, is primarily caused by diabetes and hypertension [4, 5]. Chronic obstructive pulmonary disease (COPD) involves persistent lung abnormalities and strongly linked to diabetes, which increases the risk of severe outcomes airflow [6, 7]. Diabetes, a condition marked by insufficient insulin production or utilization, contributes significantly to CKD and other complications like heart failure (HF), another condition influenced by diabetes, obesity and hypertension [8–10]. Despite billions of dollars spent on management and treatment [2, 11–13], only 66% of people living with multiple chronic diseases receive the necessary treatment [14].

The impact of living with chronic disease can be reduced through policies and interventions such as eHealth interventions that support self-management, provide tailored care, and reduce health differences based on socioeconomic factors [15]. Canada spends more than $80B annually on chronic disease management [2], with the eHealth segment dominating the market at an estimated revenue value of $2.14B in 2024 [16]. Industry growth is driven by advancements in technology (e.g., machine learning, artificial intelligence, telehealth apps [17], reduced healthcare costs, and a rising preference for remote monitoring using advanced technology [18]. Sustainable eHealth implementation in CDM requires a comprehensive understanding of the implementation processes.

The rising prevalence of chronic diseases and staff shortages in healthcare systems have led to inequitable access, inadequate care, and rising costs [19, 20]. The COVID-19 pandemic has exacerbated these issues [14, 21]. The health system is expected to transform in the next decade, driven by digital health advancements and shifts from acute to long-term care approaches [21, 22]. Digital health is a broad umbrella term encompassing the use of ehealth, defined as the use of information and communications technology for better health and related outcomes [23]. The focus of this review is on eHealth designed to promote chronic disease management by improving access to and coordination of care and health outcomes [24] More specifically, ehealth interventions included are those that clearly: (i) aim to improve self-management (ii) use peripheral devices (e.g., blood pressure cuff, weighing scale) and (iii) involve active engagement between the patient and healthcare provider [25] (see Table 4 for more details). Some examples of ehealth interventions included in our review include the *Home Telehealth (HT) Program* by the Veterans Health Administration (VHA) in the United States for chronic disease patients including diabetes [26], the *TeleCare North* project in Denmark for COPD and HF patients [27], and *Medly*, a Canadian self-management program for HF patients [28]. The focus of this study is on CKD, COPD, diabetes and HF.

The COVID-19 pandemic has accelerated the adoption of digital health interventions, with the global health market expected to reach $660B by 2025, with North America and Europe being the largest contributors in 2021 [17, 18, 29–33]. Canada’s digital health market is expected to grow (2018–2027 forecast period), with the eHealth segment expected to reach a value of 2.6B USD by 2027 [34]. Ehealth has proven to be beneficial during the pandemic by providing a variety of care services (e.g., diagnosis, treatment, and active health management, despite limited medical resources) [35].

Ehealth offers a critical solution for addressing the growing demands of CDM [36]. Despite its potential, slow and uneven implementation can lead to negative impacts (e.g., resource planning), underscoring the need for healthcare practitioners, leaders and policy-makers to adopt precise and contextually appropriate implementation processes [25, 37, 38]. Effective ehealth implementation requires addressing complex factors such as individual digital literacy, organizational workflows, and socio-economic barriers, which can significantly influence implementation success [25, 39, 40]. While ease of use is a common facilitator, limited digital literacy remains a key challenge [41, 42]. Mixed evidence and methodological limitations highlight the need for more robust studies to optimize ehealth implementation in CDM [43, 44].

### Rationale for an updated and comprehensive review

The implementation of eHealth in chronic disease management (CDM) is a complex process involving multilevel factors including as technology interoperability [45, 46], provider training, and patient engagement [47]. For example, despite robust policy efforts, the fragmentation of the UK’s National Health Services (NHS) system is the primary obstacle to eHealth adoption [48]. In Australia, patient perspectives (*n* = 56) on eHealth for CDM included key implementation factors such as local organizational context, clear provider roles, and engagement and integration of the intervention with existing clinical pathways [49].

The evolving nature of technology and the pandemic have significantly impacted eHealth implementation processes, necessitating an updated understanding of implementation processes [50], racial and social disparities among users [51], and ways to engage priority populations (e.g., those experiencing socioeconomic disadvantage, living in rural and remote areas, and Indigenous communities) [52, 53]. For example, a better understanding of patients’ preferences for a combination of in-person and virtual visits is needed despite eHealth’s ability to maintain patient-provider relationships during the pandemic [32]. Evaluating the acceptability and usability of eHealth in diverse patient populations can help individuals identify target population needs and understand perceived benefits, particularly in older or rural patients [54]. Ehealth implementation processes have been impacted in different ways since the pandemic, e.g., increased demand for digital health solutions [55, 56], changes in user attitudes [57], or streamlined regulatory processes [58, 59] warranting an updated and comprehensive understanding review.

### Rationale for realist review

Realist reviews are crucial tools for analyzing complex health interventions and identifying contexts, mechanisms, and outcomes to understand their effectiveness under different circumstances [60]. They are used by researchers, practitioners, and policymakers to understand the relationships amongst interventions, their outcomes, and features of the complex, real-world contexts in which they are implemented [61]. An informal search on Google Scholar for realist reviews published on our topic within the last 5 years emphasizes the need for an updated and comprehensive realist review inclusive of the study methodology, care setting, and community that applies to more than one type of chronic disease [25]. For example, Vassilev, et al. focused on qualitative studies (2009–2014) and HF, COPD and diabetes [62], whereas Varsi, C. et al. (2006–2018) included all study designs (qualitative, quantitative and mixed-methods), however, focused only on COPD and diabetes patients [63].

This review draws on the principles of diffusion of innovation (DOI) and dissemination and implementation science (DIS) as initial program theory (IPT) [25]. For example, in alignment with the concepts of context, mechanisms, and outcomes used in this review (Table 1), DOI describes factors affecting diffusion, including the attributes of an intervention, the characteristics of its adopters (e.g., perceptions or reactions) and the larger social or political context [64]. DIS principles focus on effective implementation strategies with outcomes related to spread, quality, or implementation reach [65]. Tables 2 and 3 include examples of the implementation strategies and outcomes considered in this review.

**Table 1:** Definitions of contexts, mechanisms and outcomes

*CMO Category*	*Definition* [25, 66, 67]
*Context*	Pre-existing conditions (e.g., people characteristics, geographic settings) outside the intervention, that influences the operation of the intervention *Example: Social context, environment, geography, policy, and regulations*
*Mechanism*	A causal force that explains why/how an outcome occurs. Typically occurs in two parts: resources ordered (action: e.g., nurse advice) related to intervention and cognitive/emotional response (response: e.g., trust). Mechanisms are often hidden, patterned behavior (e.g., people's reactions to the implementation) and can occur at different levels of analysis (e.g., intra-personal (e.g., motivation), interpersonal (e.g., sharing), organizational (e.g., leading), community (e.g., restructuring) etc *Example: Advice is given by a health provider, trust*
*Outcome*	Intended and/or unintended effects of implementation and/or process (because of a context-mechanism interaction) *Example: On-going demand for system change, interoperability issues, supply of vendors in the marketplace, and tailored care plans*

**Table 2:** Description of implementation strategies and processes

*Implementation Strategy/Process*	*Description* [63]
*Engage users*	Involving, preparing, and intervening with patients and the market to involve them and increase demand for clinical innovation
*Use evaluative and iterative strategies*	Planning and conducting the implementation process, including activities such as planning, assessing for readiness, identifying barriers and facilitators, evaluating performance and progress, and providing audit and feedback
*Change infrastructure*	Changing external structures such as legislation models, as well as internal conditions such as facilities and equipment
*Adapt and tailor to the context*	Tailoring the innovation to meet local needs and tailoring the implementation strategies toward the identified barriers and facilitators
*Develop stakeholder interrelationships*	Involving relevant internal and external stakeholders to support and move the implementation process forward
*Use financial strategies*	Changing the patient billing systems, fee structures, reimbursement policies, research funding, and clinician incentives
*Support clinicians*	Supporting clinical staff performance
*Provide interactive assistance*	Supporting implementation issues
*Train and educate stakeholders*	Providing written and oral training

**Table 3:** Description of implementation outcomes

*Implementation outcome*	*Description* [63]
*Acceptability*	Perception that a given treatment, service, practice, or innovation is agreeable, palatable, or satisfactory
*Adoption*	Intention, initial decision, or action to try or employ an innovation or evidence-based practice
*Appropriateness*	Perceived fit, relevance, or compatibility of the innovation or evidence-based practice for a given practice setting, provider, or consumer and/or perceived fit of the innovation to address a particular issue or problem
*Cost*	Cost impact of an implementation effort (incremental or implementation cost)
*Feasibility*	Extent to which a new treatment or innovation can be successfully used or carried out within a given agency or setting
*Fidelity*	Degree to which an intervention was implemented as it was prescribed in the original protocol or intended by the program developers
*Penetration*	Integration of a practice within a service setting and its subsystems
*Sustainability*	Extent to which a newly implemented treatment is maintained or institutionalized within a service setting’s ongoing, stable

## Methods

### Objectives and focus of the review

This review aims to provide a comprehensive understanding of the factors influencing the implementation of eHealth interventions for managing chronic disease CDM (HF, COPD, CKD and diabetes). The objective is to synthesize critical evidence into configurations of context, mechanisms, and outcomes (CMOs) to answer the following research question: *What CMOs are critical to eHealth implementation in CDM?* This paper reports the CMO configurations found. Descriptive findings (i.e. intervention characteristics) resulting from the review will be published elsewhere. This review refines program theory by presenting evidence demonstrating how particular mechanisms generate particular outcomes [68] as middle-range theories.

### Design

Pawson’s framework [60] was used iteratively to conduct this realist review, following the RAMESES standards [69] to ensure methodological rigour. The process involved: (i) defining the scope of the review, (ii) searching for evidence, (iii) evaluating documents, and (iv) extracting, analyzing and synthesizing evidence.

### Scope and search

**Fig. 1:**
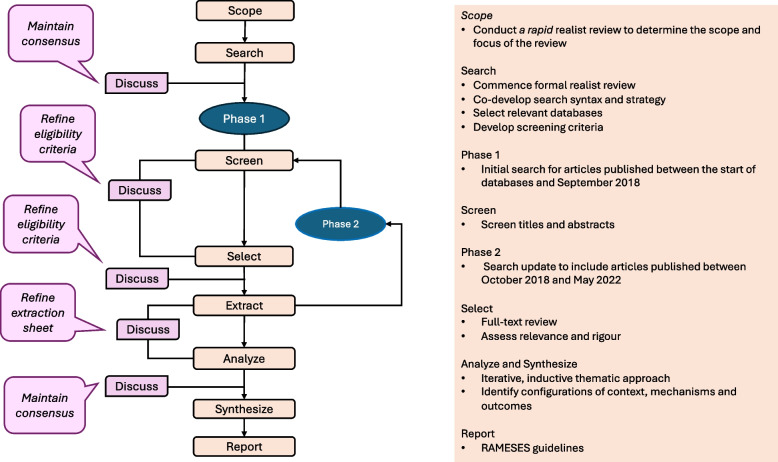
Iterative search process

A rapid realist review was first conducted to determine the initial scope and focus of the review [25], followed by a formal literature search (Fig. 1). The final search strategy was codeveloped with a medical information specialist (JB) (Supplementary Materials 1 and 2). Search terms related to ‘ehealth’, ‘health care delivery or organization’ and chronic diseases (e.g., HF, COPD, diabetes) within the scope of the review were used. The literature search was conducted in two phases (until September 2018 and October 2018-May 2022) using five databases: Medline Ovid, Embase Ovid, Cochrane Library, CINAHL Ebsco, and PsycInfo. The initial search was developed in Medline Ovid syntax and subsequently translated into the appropriate syntax for each database.

Two reviewers (NS and GP) independently screened titles and abstracts using Covidence [70]. Articles deemed eligible, ineligible, or uncertain were randomly selected for discussion to ensure reliability and consensus (Fig. 1). The inclusion criteria were further refined and applied for the remainder of the study (Table 4).

**Table 4:** Review screening criteria

	*Inclusion*	*Exclusion*
*Publication type*	• Peer-reviewed and grey literature (e.g., white papers, reports), Abstracts with information for data extraction • Empirical (qualitative, quantitative and mixed-methods design) or nonempirical literature (e.g., conceptual or theoretical) • Topic relates to implementation processes and/or outcomes related to implementation (e.g., acceptability, adoption, appropriateness, cost or feasibility of implementing an ehealth intervention) • Includes discussion of factors contributing to the implementation of ehealth intervention	• Editorials, Opinions, Commentaries, Abstracts with limited information • Does not relate to/discuss implementation and/ or adoption processes and outcomes • Does not relate to/discuss factors related to processes and/or outcome of implementing an ehealth Intervention • General feasibility study without information related to implementation
*Intervention*	• Includes combination of an active (or live) engagement of a healthcare provider and remote monitoring of patient condition using peripheral digital health device technology (e.g., blood pressure cuff, weighing scale, oximeter) • Overall aim is to improve patient self-management (i.e., ability to manage daily tasks living with the chronic condition) • Defined and/or described in manuscript (e.g., XYZ device, using ABC peripheral device vs. general reference to an ehealth intervention)	• Does not include combination of an active (or live) engagement of a healthcare provider and remote monitoring or patient condition using peripheral digital health device technology (e.g., use of telehealth websites, video consultations, telephone-only engagement without the use of monitoring equipment) • Overall aim is not to improve patient self-management (i.e., treatment of severe or short-term episode of illness, deterioration of health requiring immediate medical attention) • A hypothetical or general reference to ehealth intervention, e.g., ‘telehomecare’ • Does not include an active provider for self-management, e.g., is reactive in care in case of emergency etc
*Population*	• Includes diagnosis of at least one of the conditions: HF, COPD, CKD or diabetes (type 1 or 2) • Populations aged ≥ 18 years	• Chronic conditions outside study scope (e.g., cancer, mental health, pregnant women, children, youth) • Populations aged < 18 years
*Setting*	• Any care setting (e.g., primary, community or home)	

### Selection, appraisal and extraction

Using Endnote [71] as a reference manager, articles were reviewed in full text and evaluated for relevance and rigour (NS and GP). Methodological soundness was assessed based on clearly articulated research goals, relevance to the study purpose, and overall usefulness of findings for understanding CMOs. The Mixed Methods Appraisal Tool (v. 2018) was not used as originally intended due to its incompatibility with certain study designs (e.g., economic studies or literature reviews) and due to the discouragement of quality appraisal checklists in realist reviews [60]. Articles published in languages other than English were translated using DeepL [72].

Data fields included study characteristics, eHealth characteristics, information related to context, mechanisms and outcomes, study conclusions and any knowledge gaps reported. The extraction field was refined based on the richness of available information. A glossary was created for consistency and transparency (Tables 1– 3), and the data were extracted with MS Excel (NS and GP). Considering the subjective nature of interpretation and the need for transparency, the data extracted were coded for the data extractor’s confidence in their ability to identify the CMO (i.e., confident, or not confident) from the article. Deciding not to use the MMAT and agreeing to include confidence level in extracting data were the only two changes made to the review process that were initially planned.

### Analysis and synthesis

The analysis and synthesis followed an iterative process using an inductive thematic analysis approach [73]. The raw data were first reviewed for familiarity and then coded for emergent features. For example, the analysis led to four subcategories (system-, program-, provider-, and patient-level information). Mechanisms comprised factors associated with actions or responses and could be further categorized as positive (facilitator) or negative (barrier) factors. Outcome data were synthesized to indicate anticipated and unanticipated outcomes.

The realist approach involved identifying underlying causal mechanisms and exploring how they work under what conditions [74]. CMOs were mapped by starting with the mechanisms and working backward to identify the context and outcomes. Others have reported starting with outcomes and working backward to identify mechanism and context or articulating the CMO configuration as an ‘if, then’ statement [75]. The configurations were further synthesized into overarching themes of mechanisms and listed in order of prominence. Further details on the methodology of this review will be published separately.

Quantitative studies were analyzed by mapping the findings onto the CMO framework used for data collection. For example, a cost-analysis study of an ehealth intervention for HF patients in primary care (context) reports most of the programme costs (outcome) are attributed to program equipment (mechanism).

## Results

**Fig. 2:**
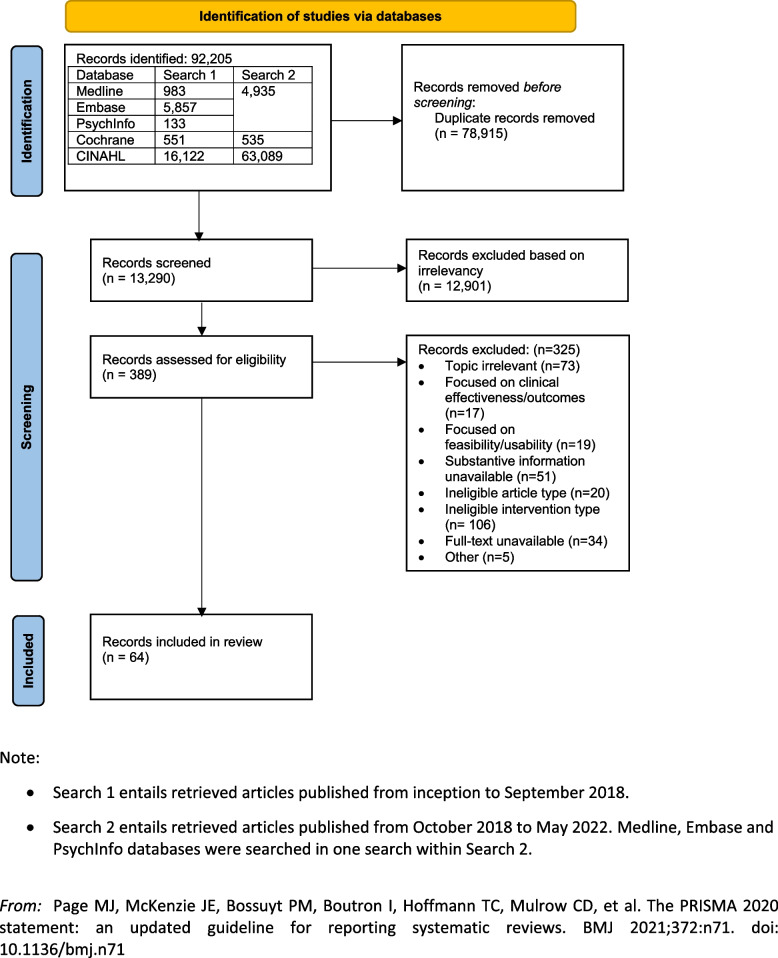
PRISMA flowchart

The search was conducted in two main phases, yielding 91,935 articles. The initial search (literature published through September 2018) resulted in 6681 articles, 42 of which contributed to the synthesis. The updated search (October 2018–May 2022) resulted in 6609 articles, 22 of which contributed to the synthesis. A total of 64 articles were included in the final synthesis (Fig. 2).

### Study characteristics

Of the 64 articles included, most took a qualitative approach (*n* = 54), focusing on diabetes care (*n* = 20) and patients (*n* = 27) as participants. Sample sizes ranged from *n* = 7 to 37 articles if in the form of a literature review, and *n* = 7 to 1573 if an empirical study (e.g., experimental or observational). The average age of patient participants was 65 years with females accounting for 2.5–100% of the participants. See Table 5 for details.

**Table 5:** Study characteristics

***Study ID***	***Authors (Year); Country***	***Approach*** ***▸ Design***	***Primary Condition(s)***	***Participant Type(s)***	***Sample Size***	***Female N (%)***	**Average Patient Age (years)**
1	Nanevicz T et al. (2000); USA [77]	Quantitative▸Descriptive	HF	Patients, Providers	N=50・Patients (n=50)	10 (20%)	60
7	Kjellstrom B et al. (2005); USA [147]	Quantitative▸Descriptive	HF	Patients	N=20	11 (55%)	59
8	Scherr D et al. (2006); Austria [78]	Quantitative▸Descriptive	HF	Patients	N=14	1 (6%)	50
10	Hopp FP et al. (2007); USA [148]	Qualitative▸Observational	Diabetes	Providers	N=10	Not reported	Not reported
11	Jaana M (2007); Canada [79]	Qualitative▸Literature review	Diabetes	Not reported	17 articles	Not reported	40
13	Kleinpell RM (2007); USA [124]	Quantitative▸Descriptive	HF	Patients	N=10	5 (50%)	74
16	Tudiver F et al. (2007); USA [123]	Mixed Methods▸Descriptive	Diabetes	Providers	N=116	32 (28%)	48
19	Horton K. (2008); UK [117]	Qualitative▸Descriptive	COPD	Other: Homecare Teams and social care staff	Not reported	Not reported	Not reported
21	Liddy C et al. (2008); Canada [80]	Mixed Methods▸Descriptive	Combination(Diabetes, Cardiovascular disease, COPD)	Patients	N=22	10 (45%)	73
22	Masella C, Zanaboni P, Di Stasi F, et al. (2008); [81]	Qualitative▸Observational	HF	Patients, Providers	N=67	13 (20%)	64
23	Trief PM et al. (2008); USA [118]	Qualitative▸Observational	Diabetes	Patients	N=25	17 (68%)	67.93
26	Sandberg J et al. (2009); USA [108]	Qualitative▸Observational	Diabetes	Providers	N=10	10 (100%)	47
27	Watson AJ, Kvedar JC, Rahman B, et al. (2009) [100]	Quantitative▸Descriptive	Diabetes	Patients, Providers	N=7・Patients (n=3)・Providers (n=4)	4 (57%)	51
28	Whitten P, Bergman A, Meese MA, et al. (2009); USA [95]	Mixed Methods▸Descriptive	HF	Patients	N=50・Interviews (n=35)	33 (66%)	78
34	Nijland N, van Gemert-Pijnen JE, Kelders SM, et al. (2011) [101]	Mixed Methods▸Descriptive	Diabetes	Patients, Providers	N=50	13 (26%)	61
35	O'Hanlon A, et al. (2011); Ireland [89]	Qualitative▸Observational	Combination(Diabetes, Heart disease)	Patients	N=40・Intervention (n=30)・Control (n=10)	Not reported	Not reported
37	Chau JPC. et al. (2012); Hong Kong [96]	Qualitative▸Observational	COPD	Patients	N=40・Intervention (n=22)・Control (n=18)	1(2.5%)	72.86
38	Fairbrother P. et al (2012); Scotland [82]	Qualitative▸Observational	COPD	Patients, Providers, Administrators/Decision-makers	N=70・Patients (n=38)・Stakeholders (n=32)	20 (53%) (Patients)	67.5
41	Seto E, Leonard KJ, Cafazzo JA, et al. (2012); Canada [97]	Qualitative▸Observational	HF	Patients, Providers	N=70・Patients (n=22)・Clinicians (n=5)	4 (18%)	57
43	Ure J. et al. (2012); UK [119]	Mixed Methods▸Observational	COPD	Patients, Administrators	N=45・Professionals (n=25)・Patients (n=20)	・Professionals: 35%・Patients: 39%	68.9
49	Carlisle K, Warren R. (2013) [109]	Mixed Methods▸Experimental	Diabetes	Patients, Providers	N=12・Patients (n=4)・Healthcare practioners (n=8)	Not reported	Not reported
51	Chang CP. et al. (2013); Taiwan [83]	Qualitative▸Observational	Diabetes	Providers	N=9	9 (100%)	36.5(26-47 years)
54	Henderson C. et al. (2013); England [110]	Qualitative▸Experimental	Combination (COPD, Diabetes, HF)	Patients	N=1573・Control (n=728)・Intervention (n=845)	・Control: 290 (40%)・Intervention: 347 (41%)	・Control: 70.6・Intervention: 70.1
55	Hiratsuka V, Delafield R, Starks H, et al. (2013) [91]	Qualitative▸Observational	Diabetes	Patients, Providers	N=40・Focus groups (n=6)	30 (76%)	45+
60	Brewster L. et al. (2014); UK [88]	Qualitative▸Literature review	Combination (CHF, COPD)	Administrators/Decision-makers	Not reported	Not reported	Not reported
62	Fairbrother P. et al. (2014); Scotland [92]	Qualitative▸Descriptive	HF	Patients, Administrators	N=23・Patients (n=18)・Health professionals (n=5)	7 (39%) (Patients)	75
63	Gorst SL. et al. (2014); UK [86]	Qualitative▸Literature review	Combination (COPD, HF)	Patients	37 articles	12 (38-41%)	65-68
65	Koopman RJ, Wakefield BJ, Johanning JL, et al. (2014) [122]	Qualitative▸Observational	Diabetes	Patients, Providers	N=23・Patients (n=93)・Physicians (n=12)Nurses (n=6)	Not reported	Not reported
67	Rho MJ. et al. (2014); South Korea [111]	Quantitative▸Descriptive	Diabetes	Patients	N=81	30 (37%)	50 years
68	Sharma U, Clarke M. (2014); UK [121]	Qualitative▸Observational	HF	Providers	Focus groups (4-8 participants per group) (n=3)Interviews (n=8)	Not reported	Not reported
71	Brunton L. et al. (2015); UK [102]	Qualitative▸Literature review	COPD	Other: Patients, Health Professionals	10 articles	65 (51%)	67.8
73	Hanley J, Fairbrother P, McCloughan L, et al. (2015) [149]	Qualitative▸Observational	Diabetes	Patients, Providers	N=33・Patients (n=23)・Physicians (n=4)Nurses (n=6)	7 (30%)	60
74	Hunting G. et al. (2015); Canada [87]	Qualitative▸Descriptive	Combination(COPD, HF)	Patients, Providers, Administrators/Decision-makers	N=89・Patient/Caregiver (n=39)・Healthcare provider (n=23)・Technician (n=2)・Administrator (n=12)・Decision-maker (n=13)	19 (49%) (Patients)	73
76	Stoddart A. et al. (2015); UK [150]	Quantitative▸Experimental	COPD	Patients	N=256・Control (n=128)・Intervention (n=128)	・Control:65 (51%)・Intervention: 76 (59%)	・Control: 68.4・Intervention: 69.4
77	Taylor J. et al. (2015); UK [93]	Qualitative▸Descriptive	Combination (CHF, COPD)	Patients, Providers, Administrators/Decision-makers	N=58・Staff (n=57)・Patient (n=1)	Not reported	Not reported
79	Vassilev I. et al. (2015); UK [62]	Qualitative▸Literature review	Combination (COPD, Diabetes, HF)	Patients, Providers, Administrators/Decision-makers	15 articles	Not reported	Not reported
87	Pekmezaris R et al. (2016); USA [151]	Qualitative▸Observational	HF	Other: Patients, caregivers, patient advocates, providers, health policy and finance, disparities experts	N=18Focus group 1 and 2 (n=14)Focus group 3 (n=4)	Not reported	Not reported
93	Alvarado MM. et al. (2017); USA [115]	Qualitative▸Literature review	Diabetes	Patients	53 articles	Varied	50s
94	Ditchburn JL. et al.. (2017); UK [98]	Qualitative▸Observational	CKD	Administrators/decision-makers	N=10	Not reported	Not reported
99	Vatnoy TK. Et al. (2017); Norway [90]	Qualitative▸Observational	COPD	Patients	N=10	3 (30%)	64.5
100	Vest BM, Hall VM, Kahn LS, et al. (2017); USA [103]	Qualitative▸Observational	Diabetes	Providers	N=8	Not reported	Not reported
103	Jaana M, et al. (2019); Canada [152]	Quantitative▸Descriptive	HF	Patients	N=23	7 (30%)	75.2
*117*	Alghamdi, S. M. et al. (2021); Saudi Arabia [120]	Qualitative▸Literature review	COPD	Patients	27 articles・COPD patients (n=4157)	Not reported	65
*118*	Alodhayani, A. A. et al. (2021); Saudi Arabia [125]	Qualitative▸Observational	Combination (Diabetes, Hypertension)	Providers, Administrators	N=7・Nurse (n=4)・Physician (n=2)・Information Technology (n=1)	3 (43%)	Not reported
*124*	Ammenwerth, E. et al. (2018); Austria [153]	Quantitative▸Descriptive	HF	Patients	N=28	5 (18%)	64.5
*170*	Chua, V. et al. (2022); Singapore [154]	Qualitative▸Literature review	Combination (CHF, Diabetes)	Patients	11 articles	Not reported	Not reported
*171*	Clarke, M. et al. (2018); UK [155]	Quantitative▸Descriptive	COPD	Patients	N=227	112 (50%)	70.9
*219*	Gordon, K. et al. (2020); Canada [94]	Mixed Methods▸Descriptive	Combination (Diabetes, HF)	Patients	N=26・Patients (n=17)	9 (53%)	73.8
*221*	Hanley, J.; Pinnock, H.; Paterson, M.; McKinstry, B. (2018) [112]	Qualitative▸Literature review	Combination (COPD, Diabetes, HF)	Patients, Providers	7 articles	N not reported (40%) (Patients)	66.4
*222*	Haynes, S. C. et al. (2020); USA [104]	Qualitative▸Observational	HF	Patients	N=12	4 (33%)	76
*267*	Lee, J. Y. et al. (2019); Malaysia [156]	Qualitative▸Descriptive	Diabetes	Patients	N=48・Focus groups (n=12)・ Interviews (n=2)	27 (56.3%)	51.9
*303*	Michaud, T. L. et al. (2023); USA [157]	Quantitative▸Descriptive	Diabetes	Other: Healthcare system	N=3307・ Intervention (n=1943)・ Control (n=1364)	Not reported	Not reported
305	Michaud, T. L.; Zhou, J.; McCarthy, M. A.; Siahpush, M.; Su, D. (2018); USA [158]	Quantitative▸Descriptive	Combination (COPD, Diabetes, HF)	Other: Ehealth intervention	12 articles	Not reported	Not reported
*312*	Nathania, J. et al. (2022); Singapore [105]	Mixed Methods▸Descriptive	HF	Patients	N=37・Interviewed (n=19)	8 (22%)	65.1
*315*	Nelson, L. A. et al. (2020); USA [126]	Qualitative▸Literature review	Diabetes	Patients, Providers, Administrators/Decision-makers	11 articles	Not reported	Not reported
*340*	Ross, J. et al. (2018); UK [84]	Qualitative▸Other	Diabetes	Other: Ehealth intervention	Not reported	Not reported	Not reported
*349*	Schmaderer, Myra et al. (2021); USA [106]	Qualitative▸Descriptive	HF	Patients	N=10	4(40%)	55.8
*353*	Seto, Emily; Morita, Plinio Pelegrini; Tomkun, Jonathan; et al. (2019); Canada [107]	Mixed Methods▸Descriptive	HF	Patients, Providers	N=7・ Patients (n=6)・ Cardiologist (n=1)	Not reported	Not reported
*357*	Sim, R et al (2021); Malaysia [99]	Qualitative▸Literature review	Diabetes	Patients	20 articles	Not reported	56-59
*371*	Van Lieshout, F. et al. (2020); Canada [45]	Qualitative▸Observational	COPD	Patients, Providers, Administrators/Decision-makers	N=16・Patient/Caregiver (n=8)・Healthcare provider (n=8)	5 (62%) (Patient/Caregiver)	74.6
*378*	Wali, Sahr et al. (2021); Australia [159]	Qualitative▸Descriptive	HF	Patients, Providers, Administrators/Decision-makers	N= 29・Patients (n=16)・Clinicians (n=9),・Operational staff (n=4)	8 (50%) (Patients)	54.5
*380*	Walker, R. C. et al. (2019); Australia [54]	Qualitative▸Literature review	Combination (CKD, COPD, HF, Diabetes)	Patients	16 articles・Patients (n=307)	Not reported	64.3
386	Wilson, Jessica; Heinsch, Milena; Betts, David; et al. (2021); Australia [85]	Qualitative▸Literature review	Combination (HF, Cadiovascular disease)	Patients	14 articles・Patients (n=137)	Not reported	69.5
*394*	Zaman, Sojib Bin et al. (2022); Australia [114]	Qualitative▸Literature review	Combination (Cardiovascular disease, COPD, Diabetes)	Patients, Providers	31 articles	Not reported	Not reported

### Context

Context refers to pre-existing conditions, independent of the eHealth intervention. The expanded categories identified occurred at four broad levels: intervention setting, intervention characteristics, provider characteristics, and patient-level factors (Table 6) [76]. Four key levels of care were identified: primary, secondary, tertiary, and home or community care. Levels of care were identified according to the article description or where the intervention was described (e.g., an eHealth intervention program based on an HF clinic in an urban hospital). Table 6 illustrates the different categories, subcategories and themes of information related to context.

**Table 6:** Emerging categories, subcategories and themes related to context

Category	Sub-category	Theme	Mentions (n)
Setting	Country	International settings	10
	Care-level	Primary care	13
		Tertiary care	8
		Community/home care	6
		Primary and community/home care	2
	Community	Varied	8
		Urban	4
		Underserved	3
		Semi-rural	1
		Other: Native communities and/or villages	1
Intervention	Features	Range of modalities and approaches used	6
		Technical support/training provided	4
	Implementation	Beyond pilot phase	9
		Pilot phase	3
Provider	Roles	Healthcare professionals (team-based approach)	17
		Physicians and Nurses	7
		Nurses	3
		Physicians	1
	Experience	Experience with ehealth as an intervention	5
		Experience with CDM	5
Patient	Patient factors	Elderly (65 years or older)	6
		Poorly controlled disease or at-risk of complications	5
		Mixed educational and/or ethnic backgrounds	5

### Mechanisms

**Fig. 3:**
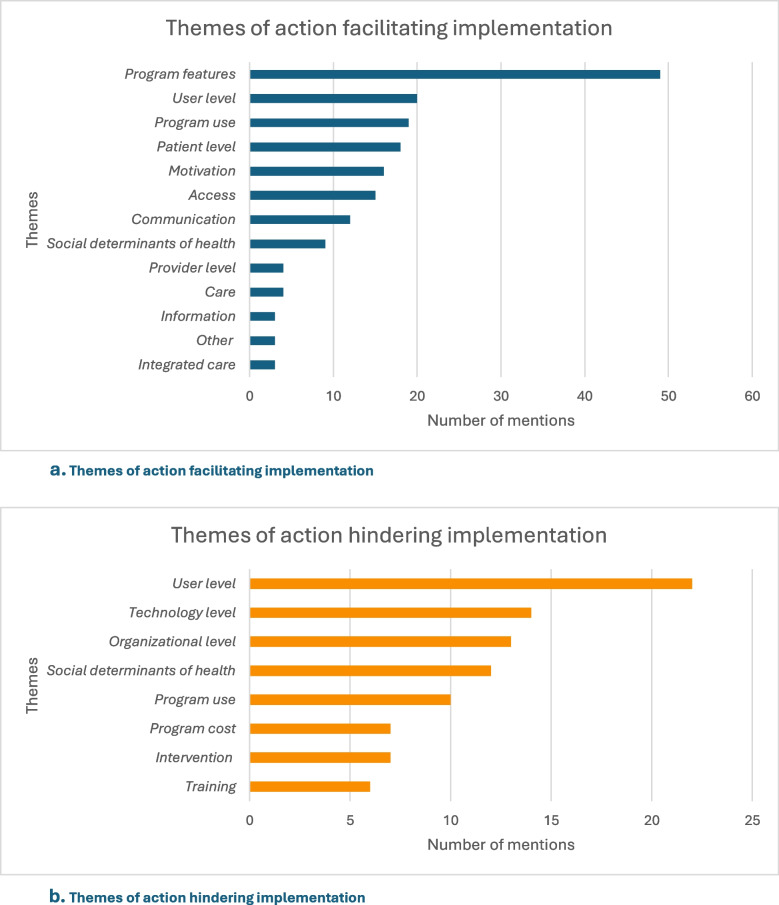
**a** and **b **Mechanism related themes influencing implementation

Mechanisms explain an implementation outcome and are often a manifestation of hidden behavior. User-level factors (e.g., building relationships, perceived usefulness, and digital literacy) and program use are broad themes of action associated with facilitators and barriers. Program features were most frequently described as facilitators, while user-level factors were generally described as barriers (Fig. 3a and b). Responses were found across multiple levels (e.g., user, patient, provider, organization). Supplementary Materials 3 and 4 include a detailed overview of broad and specific themes of actions and responses associated with facilitating and hindering eHealth implementation.

**Fig. 4:**
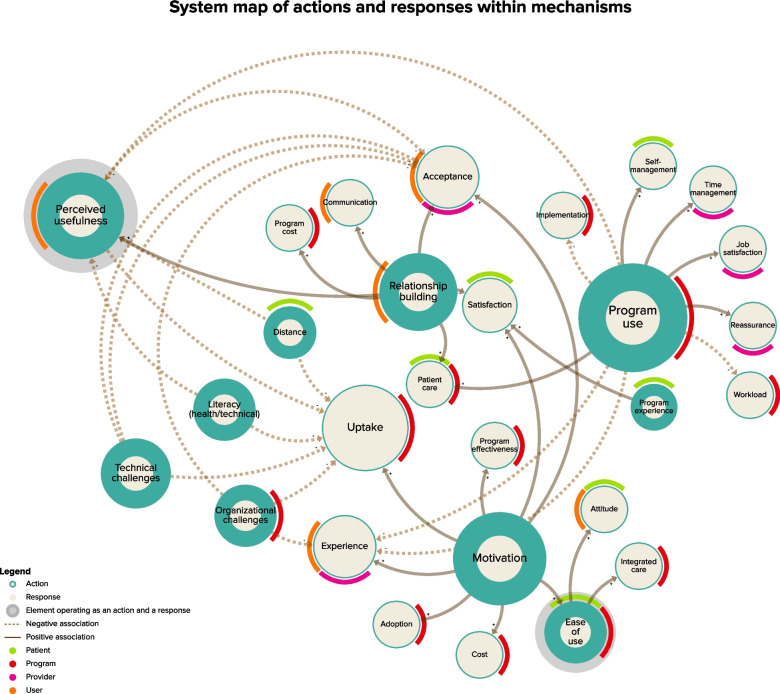
System map of actions and responses within mechanisms

A systems map of the actions and responses within the mechanisms found in the resulting CMO is presented in Fig. 4. The map illustrates key themes of actions and responses based on the number of connections (i.e., the larger the sphere is, the more connections there are). Action elements are tagged with colours corresponding to their mechanism level (i.e., program, patient, etc.). A negative association between an action and a response is indicated with a dotted line, and a positive association is reflected through a continued line. Program use, perceived usefulness and motivation are key themes of action. Program uptake and acceptance are key themes of response. Perceived usefulness emerged as a theme of action and response.

### Outcomes

This review examines outcomes as intended and/or unintended effects of implementation and/or processes. The 64 studies included implementation outcomes (*n* = 13) and experiences (*n* = 13), followed by acceptance (*n* = 10) and feasibility (*n* = 9). The expected outcomes were mainly acceptance (*n* = 6) and satisfaction (*n* = 5), while the unanticipated outcomes varied (e.g., perceived usefulness, benefits, and impact of using the intervention). Most studies reported successful implementation (*n* = 39); however, a significant portion of studies (*n* = 22) were ambiguous due to the nature of the investigation and studied outcome (e.g., implementation factors, views, perspectives).

### CMO Configurations

**Fig. 5:**
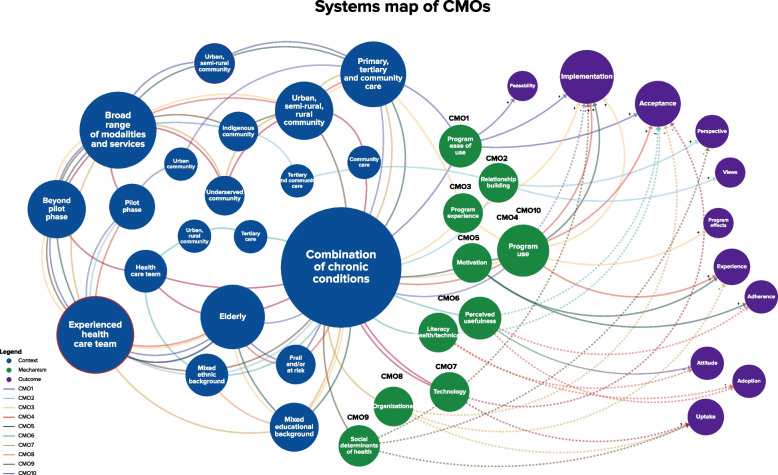
System map of CMOs

Thematic analysis identified a total of 12 configurations. The predominant (comprised of most commonly occurring themes) configurations facilitating (*n* = 5) and hindering (*n* = 5) eHealth implementation in CDM are described below (Fig. 5). Studies included in analysis are quoted when possible to strengthen inferential explanations of the CMOs. A detailed list of CMO configurations is included in Supplementary Materials 5 and 6, and a visual breakdown is provided in Supplementary Material 7. An overview of emerging individual facilitators and barriers will be published separately.

Figure 5 illustrates key features (e.g., an experienced health care team, a multimodal intervention) of context and themes of action (e.g., program use) within mechanisms and outcomes (e.g., implementation outcomes, acceptance) critical to eHealth implementation.

### Facilitating eHealth implementation configurations

#### Configuration 1 (CMO1) effectiveness of ehealth through care integration and user-centered design

If a widely implemented multimodal eHealth intervention is administered by an experienced healthcare team in urban and semirural communities across different care settings (C), then program features such as ease of use are associated with better patient- and user-level outcomes (e.g., satisfaction) and programs (e.g., integrated care) (M), in turn facilitating implementation outcomes, program acceptance and feasibility (O) [54, 77–90]. This is illustrated by the following direct quote from one of the source articles upon which CMO1 is based on, in which the authors study a telemonitoring service for COPD patients administered by a team of experienced health professionals (community respiratory physiotherapists, nurses or general physicians) across four geographical regions in Scotland.

“I think it's very good. It makes you feel like somebody’s looking after you. If anything goes wrong, you can get in touch with them any time you want ... you’ve got the confidence that they're going to get something done. I can't fault them anyway. (Male, 79 years)” [82].

#### Configuration 2 (CMO2) strengthening patient and program outcomes through relationship building in dispersed communities

If a pilot multimodal intervention is implemented in dispersed communities, including Indigenous communities, across tertiary and community care settings by an experienced health care team (C), then building relationships is associated with improved patient (e.g., satisfaction), user (e.g., perceived usefulness) and program-level (e.g., cost) outcomes (M), in turn facilitating perspectives and views (O) [45, 82, 91–94]. A quote from one of the source articles for CMO2 is presented here for illustration. The authors assess the implementation of an ehealth intervention by an experienced healthcare team (respiratory therapists to care for COPD patients in community-based hospitals as part of a randomized controlled trial (RCT).

“So a lot of the patients call me because they know I’ll answer and it’s just to help get a prescription filled or sort of some other issue that they’re having… cause they know I’ll answer and have access to their physicians. They’ll call me over calling [the doctor’s] receptionist… Which maybe isn’t good, but, is also kind of good for [patients] because they feel more comfortable. Cause the relationship is there. [Allied Health Professional, very high interaction]” [45].

#### Configuration 3 (CMO3) enhancing user satisfaction through accessible training

If a widely implemented multimodal intervention is implemented across all communities and care settings with the option of leasing equipment and accessing education (C), then patient program experience is associated with improved satisfaction levels (M), in turn facilitating implementation outcomes (O) [80, 95–99]. This is illustrated by the following quote from Ditchburn, et al. who studied the experiences of providers (doctors, renal nurses, technical staff) caring for CKD patients with ehealth service with regional coverage across an area with 1.4M people. “‘Before, with a new patient, I didn’t feel comfortable coming in here [referring to nurses’ room] whereas now I can keep my eye on her [referring to a patient currently on remote dialysis] and do some work on the computer, finish my emails off, desk work’ (renal nurse)” [98].

#### Configuration 4 (CMO4) empowering the elderly through program use: enhancing patient care and provider efficiency

If a multimodal intervention is piloted across different communities and care settings for elderly and frail populations (C), then program use is associated with better patient (e.g., self-management, care) and provider (e.g., time management) outcomes (M), in turn facilitating implementation, user experiences and acceptance [54, 83, 98–107]. The following is a direct patient quote from a pilot study of an ehealth program designed for diabetes patients and administered within the Massachusetts General Hospital practice by an experienced healthcare team (nurse practitioner, nurse, nutritionist). “Sometimes I forget to take my blood glucose, but if I knew that someone was looking at them, then I will be more compliant” [100].

#### Configuration 5 (CMO5) driving program and patient success through motivation

If a widely implemented multimodal intervention is administered by an experienced healthcare team across all communities and care settings (C), then motivation is associated with improved program (e.g., uptake, cost-effectiveness), patient (e.g., satisfaction) and user-level (e.g., acceptance) outcomes (M), in turn facilitating implementation outcomes, patient adherence and experiences [62, 80, 86, 87, 91, 92, 103, 104, 108–114]. One of the source articles underpinning CMO5 is a realist review conducted by Vassilev et al. that reported several core mechanisms essential to implementing ehealth interventions in CDM. Here, I share a quote from the perspective of a provider on the role of motivation for chronic disease patient in securing internet connection which is also essential to using the ehealth intervention.

“We teach them to write e-mails. And there was one man, he had a son who lives in Japan. And in the meantime he has become a grandparent, but he had never heard of the internet. So he got this internet connection at home, and his son sent him his email address. And I helped him type the email address, and when he got an answer he got pictures and saw his grandchild for the first time. Really, if you see this older man looking a picture with tears in his eyes” [62].

### Configurations hindering eHealth implementation

Overall, eight configurations hindering eHealth implementation were found. The five most prominent CMOs are described below.

#### Configuration 6 (CMO6) impact of user-level factors on program outcomes across diverse settings

If an eHealth intervention is implemented by a healthcare team in urban and rural communities in a tertiary care setting (C), then user-level factors such as perceived usefulness and low (health/technical) literacy are associated with decreased provider (e.g., acceptance), user (e.g., perceived usability) and implementation level (e.g., uptakes) outcomes (M), in turn hindering user acceptance, attitudes, adherence and program adoption [86, 88, 104, 112, 115, 116]. This is illustrated by the following quote from Haynes, et al. who studied patient adherence to CardioMEMS, a remote monitoring system provided to HF patients in tertiary care settings. “With the old machine, I just purposely didn’t take it with me because it wasn’t worth my time and effort after going three days without a reading because I couldn’t sit there for 20 min moving back and forth it just wasn’t worth it” [104].

#### Configuration 7 (CMO7) influence of technology-level challenges to ehealth implementation in community settings

If a widely implemented multimodal intervention is administered in the community setting by a healthcare team across all communities, including the underserved (C), then technology-level factors such as technical challenges are associated with decreased user (e.g., acceptance), provider (e.g., experience) and program-level (e.g., uptake) outcomes (M), in turn hindering program acceptance and uptake (O) [82, 86–88, 108, 117–120]. A quote from Alghamdi et al.’s systematic review on factors influencing overall acceptance and completion of ehealth interventions is presented here for illustration:

“Refusal to complete TH interventions is primarily attributed to the interventions themselves. It was noted that TH interventions with multiple components were fraught with complexities and technical difficulties that have resulted in decreased treatment sessions or even termination. This could lead to participant dissatisfaction and, ultimately, dropping out of the study” [120].

#### Configuration 8 (CMO8) organizational challenges and their impact across different communities and care settings

If a multimodal intervention is administered by an experienced healthcare team to a population with stable and poorly controlled chronic disease across various communities and care settings (C), then organizational-level factors such as disruption are more likely to decrease user (e.g., experience), provider (e.g., acceptance) and implementation level (e.g., uptake) outcomes (M), in turn hindering program acceptance, implementation, uptake and experiences (O) [82, 86, 88, 97, 109, 112, 115, 118, 121–123]. To illustrate this, I quote Koopman, et al. from a qualitative study on the implementation of ehealth for diabetic patients in primary care. This quote was used by the authors to support the broader theme of ehealth not being well-received by healthcare providers in a fee-for-service payment model.

“The payment model doesn’t, and the work expectations don’t. I mean because honestly, it is at this point in the development of an expanded primary care model, this is just an add-on. I mean I don’t see two fewer patients so that I can go through all these messages or anything like that. It just becomes an add-on. And that’s of course what you hear many primary care physicians lamenting, ‘‘This is just one more thing that I have to do that I don’t get paid for.’’” (Physician 2) [122]

#### Configuration 9 (CMO9) influences of social determinants of success of widely implemented ehealth interventions

If a widely implemented intervention is administered by an experienced healthcare team across different communities, including Indigenous and geographically dispersed communities (C), then social determinants of health, such as geographical distance, is associated with decreased user (e.g., perceived usability) and implementation level (e.g., uptake) outcomes (M), in turn hindering implementation, uptake and user perspectives (O) [54, 86, 87, 91, 113]. To illustrate this, I quote Hiratsuka, et al.’s observations in one of the studies underpinning CMO9. The authors conducted a qualitative study on the perspectives of patients and providers using ehealth for diabetes care across two geographically dispersed healthcare systems serving Indigenous communities and villages in Hawaii and Alaska.

“It works great when it works, but let me tell you, in my clinics, it’s more not working than working. And if it is working, the staff doesn’t know how to use it. At one of my other clinics, they added on all these new tools, but by the time they [were] added on, [the staff] didn’t know quite how to use [it]. And so it’s one of those missing links of getting people together to actually get the equipment running and then making sure we have internet access, which is spotty out in ... [Rural Alaska areas] ... and having the technicians that are capable to run it on a regular basis.” [91]

### Configuration 10 (CMO10) impact of increased program involvement across different settings

If an eHealth intervention is piloted in urban communities across different settings by an experienced healthcare team (C), then increased involvement from program use is associated with decreased provider (e.g., experience), user (e.g., perceived usability), and organizational outcomes (e.g., care disruption) (M), in turn hindering implementation outcomes (O) [45, 79, 92, 97, 107, 124–126]. This is illustrated by the following quotes from a van Lieshout’s (2020) study on the implementation of ehealth in CDM based in a community hospital setting.

“So a lot of the patients call me because they know I’ll answer and it’s just to help get a prescription filled or sort of some other issue that they’re having... cause they know I’ll answer and have access to their physicians. They’ll call me over calling [the doctor’s] receptionist... Which maybe isn’t good, but, is also kind of good for [patients] because they feel more comfortable. Cause the relationship is there. [Allied Health Professional, very high interaction]”

“I was very skeptical when the study was first started because uh, a lot of the times, these sort of home monitoring or self-monitoring or other programs kind of invent the technology first before asking a lot of important clinical questions of whether it’s actually gonna be of benefit or uh, examining why patients actually have exacerbations or the mechanisms and then it’s sort of just implemented into this study and then we see what happens, so, it’s uh, a bit of the uh, cart before the horse kind of scenario [Physician, moderate interaction]” [45]

## Discussion

This review identifies factors critical to eHealth implementation in CDM in ten configurations of context, mechanisms, and outcomes. While previous realist reviews focused on specific populations, care settings, or mechanisms, and were outdated, they did not provide a comprehensive overview of factors critical to eHealth implementation in CDM.

Study findings suggest that an easy-to-use intervention, a positive experience, strong motivation, and relationships can help implementation by improving user experience in any setting and benefit the program regardless of its implementation stage (i.e., pilot vs. beyond pilot).

Regardless of the care setting or geography, the identified enabling configurations have program- and user-level mechanisms. With a mix of care settings, the identified hindering configurations have user-, provider-, or organization-level mechanisms. This finding is consistent with that of Noordman et al. who identified the most relevant contextual factors influencing the implementation of chronic disease self-management interventions as being at the patient, professional or interaction levels [127].

Gonzalez et al. suggest that the setting (e.g., clinic size and geography) in which ehealth is used is important to its implementation. For example, interventions with fewer participants, those conducted over shorter periods, or in single clinical settings supported the use of ehealth [128]. This differs from the findings presented in this study, where enabling configurations consisted of various contexts (e.g., care setting, geography, and implementation stage).

Intuitive technology providing quick and accurate information aids ehealth implementation for cardiovascular management in any care setting [40]. Examples of how ease of use of an intervention can be improved include using the technology more often, having a support (e.g., a family member or an aide) present, or using the intervention at dedicated times [129].

There is growing evidence that trusting relationships between patients and providers are critical for the effective implementation of health services [130]. Review findings are consistent with those of other studies which found the quality of the patient-provider relationship impacted a patient’s self-management behaviour [127]. An Australian study of patients’ (*n* = 153) and providers’ (*n* = 29) perspectives on predictors of using ehealth in CDM among rural populations identified relationship-building as a critical factor [131]. A realist review (*n* = 11) on the same topic by Varsi et al. identified stakeholder engagement, including training, education, and developing interrelationships, as an important part of implementation strategies [63]. Trusting relationships can pull the necessary levers for desired behaviour changes during implementation [130]. From the provider’s perspective, stronger relationships help provide timely care [132].

In line with previous research, motivation has a significant influence on how long or often a person with chronic disease will use ehealth to self-manage their condition. For example, a qualitative study (*n* = 16) on how COPD patients use ehealth found that their motivation levels affect usage over time [133]. A lack of perceived benefits and/or frustration with technology can reduce the motivation to use ehealth for CDM in any care setting [40].

Configurations found to hinder ehealth implementation ranged in terms of their context, and the information necessary to specify them was sometimes not documented. Mechanisms occurred mostly on the user and program levels and pertained to perceived usability, acceptance, and uptake.

This review found that low levels of user-perceived usefulness or (health or technical) literacy can lead to reduced acceptance or uptake in a tertiary care setting. This finding differs from that of Belachew et al. who found low levels of ehealth use among chronic disease patients (*n* = 422) in a tertiary care hospital in Ethiopia were present despite high levels of perceived usefulness (49%) and perception (71%) [134]. Digital literacy on the other hand seems to be critical to ehealth adoption [30]. Herrera et al. conducted an integrative review (*n* = 14) on ehealth implementation in chronic cardiovascular management within any care setting. They found technological literacy was a barrier to adoption if it was low, and a facilitator if it was high [40].

We found that increased workload associated with program use can negatively influence the implementation of ehealth in any care setting. Similarly, a scoping review of healthcare professionals (*n* = 19) identified factors crucial to upscaling ehealth across different geographic settings, with increased workload being a common perceived barrier [135]. Healthcare professionals face significant barriers to eHealth’s widespread use in different care settings, including infrastructure, workload, and time [136].

The findings demonstrate that the influence of context on ehealth implementation in CDM is multi-levelled. This is different from other studies that focus on works that focus on the context of healthcare at the individual level [137] or within a specific care setting [138], or that lack a clear description of the context altogether [63, 139].

Another interesting finding is that experienced healthcare teams are a key characteristic of the context for ehealth implementation. Team-based care (TBC) is a highly effective care model for complex healthcare needs, particularly in chronic disease patients with multiple providers and treatment plans [140, 141]. Digital health interventions like virtual care platforms reduce the caregiver burden by making care coordination easier [142]. TBC during eHealth implementation improves patient safety, quality of care, and satisfaction, transforming primary practice towards patient-centred care [143, 144]. A Canadian study revealed that interprofessional teams (*n* = 65) using eHealth effectively improve patient self-management in primary care settings for people with multimorbidity (*n* = 76) [145].

Policies that support context-specific implementation strategies can improve program experience across communities, settings and stages of implementation, focusing on user-centred design of interventions that adapt to population needs.

Investment in digital literacy is crucial for providing adequate training in using ehealth, including technical and educational support to patients and healthcare professionals.

Considerable gaps in our understanding remain. Research funding models that support evidence synthesis and evaluation of ehealth implementation in underserved communities or community care settings, will significantly advance our understanding of ehealth implementation.

### Strengths and limitations

There are several strengths of this review. The review addresses the need for a current and comprehensive understanding of factors critical to ehealth implementation. It contributes to advancing the field of implementation science by providing researchers with middle-range theories to test in real-world settings. Our review takes a transparent and iterative methodological approach that may be useful for future researchers in framing research questions and collecting data. With a focus on eHealth interventions implemented across all care settings, geography and countries, the findings are ‘externally valid’ to those of other similar eHealth interventions worldwide. The review places equal emphasis on context as it does on mechanisms in its exploration. These findings reflect the nuanced, dynamic and relational nature of eHealth implementation processes currently lacking in the literature [146].

There are a few limitations to consider. First, the overall aim of the study was ambitious, considering the vast amount and heterogeneity of the evidence. With an equal focus on context and mechanisms and four different chronic conditions, data collection was an iterative and lengthy process. Second, the lack of supporting literature makes it challenging to compare findings to reviews on the same topic using the same study design and approach. These qualities make the study seminal, with findings that are useful and relevant. Third, the heterogeneity of information available required subjectivity in data extraction and interpretation when identifying CMO configurations. The CMO configurations were coded 0 and 1 for better transparency.

## Conclusion

This is the first review, to our knowledge, that aims to provide a fulsome understanding of factors influencing the successful implementation of ehealth interventions, inclusive of context with a focus on four major chronic conditions, that are necessary for effectively managing chronic care in today’s strained healthcare system. It has revealed ten configurations of context, mechanisms and outcomes emerging from the vast body of implementation literature. Before this review, studies focused on specific chronic conditions, mechanisms only, or certain care settings. The findings advance our current knowledge of the contexts (e.g., care setting, geography) and mechanisms (e.g., action and response) involved in ehealth implementation processes. A key takeaway is the nonlinear and multilevel relationships existing within CMOs. More broadly, research is needed to determine causality by testing out the CMOs as middle-range theories in real-world settings. Questions arise about which aspects of context are crucial for ehealth implementation (e.g., care setting vs geography). Considerable more work is needed to determine how mechanisms can operate under different contexts.

## Supplementary Information

Supplementary Material 1.

Supplementary Material 2.

Supplementary Material 3.

Supplementary Material 4.

Supplementary Material 5.

Supplementary Material 6.

Supplementary Material 7.

## Data Availability

Data is provided within the manuscript or supplementary information files.

## Abbreviations

CDM: Chronic disease management
CINAHL: Cumilated Index to Nursing and Allied Health Literature
CKD: Chronic kidney disease
CMO: Context-Mechanism-Outcome configuration
COPD: Chronic obstructive pulmonary disease
COVID-19: Coronavirus disease of 2019
DIS: Dissemination and Implementation Science
DOI: Diffusion of Innovation Theory
HF: Heart Failure
IPT: Initial Program Theory
NHS: National Health Services
PROSPERO: International prospective register of systematic reviews
TBC: Team Based Care
US: United States
USD: United States dollar

## Footnotes

1 Cost adjusted for inflation (2024).
