# Supplementary material for: A realist review of factors critical for the implementation of eHealth in chronic disease management
Source: BMC Health Serv Res. 2025 Apr 2;25:496. doi: 10.1186/s12913-025-12361-0 (PMC11966836; doi:10.1186/s12913-025-12361-0)
Supplement: Supplementary file 1 — Supplementary Material 1. [file 12913_2025_12361_MOESM1_ESM.docx]

## Appendix 1. Phase 1 Search Strategy and Syntax (- 2018)

**Towards better understanding of factors critical for the implementation and/or adoption of initiatives using technology in heart failure, copd, chronic kidney disease, and diabetes management – realist review.**

**Search done for:** Nida Shahid <nida.shahid@theta.utoronto.ca>

**Performed by:** Joanna Bielecki (joanna.bielecki@theta.utoronto.ca)

**Databases searched:**

Medline Ovid

Embase Ovid

Cochrane Library Wiley

CINAHL Ebsco

PsycINFO Ovid

**MEDLINE SEARCH**

**Performed by:** Joanna Bielecki (joanna.bielecki@theta.utoronto.ca)

**Results sent to:** Nida Shahid <nida.shahid@theta.utoronto.ca>

**Date:** 27 September 2018

**Medline search name:** "eHealth-Telecare-Coaching-Four-Chronic-Cond-SR-Medline FINAL"; Nida Shahid Sept 2018

**Database(s):** **Ovid MEDLINE: Epub Ahead of Print, In-Process & Other Non-Indexed Citations, Ovid MEDLINE® Daily and Ovid MEDLINE®**1946-Present

**Search Strategy:**

| **#** | **Searches** | **Results** |
| --- | --- | --- |
| 1 | telemedicine/ or remote consultation/ or (((home or home-based or in-home) adj3 (telehealth or telemonitoring or telecare)) or telehealth or (mobile adj health) or mhealth or ehealth or "remote consultation$" or e-visit$ or telemedicine or telenursing or telehomecare or telecare or telemonitor$ or teleconsultation or telemanagement or telesurveillance).ti,ab. | 28437 |
| 2 | Distance Counseling/ or Health Education/ or Consumer Health Information/ or Health Literacy/ or Teach-Back Communication/ or patient education as topic/ or self care/ or behavior therapy/ or health knowledge, attitudes, practice/ or consumer participation/ or (((distance or e-health or web-based or internet-based or mhealth or m-health or telehealth or telemonitoring or telecare or "mobile health") adj2 (counseling or coaching or training or therap$)) or e-therap$ or "e therap$" or e-counseling or "e counseling" or "asynchronous communication?" or ((health or patient? or consumer?) adj2 education) or "self care" or self-care or self-management or self-efficacy or "self management" or "self efficacy" or ((conditioning or behavio?r) adj2 therap$) or "behavio?r modification?" or ((consumer? or patient? or public or communit$) adj2 (participation or involvement or action?))).ti,ab. | 371582 |
| 3 | exp heart failure/ or (((heart or myocardial or cardiac or diastolic or systolic) adj3 (fail$ or decompensation or dysfunction$)) or ((cardio renal or cardio-renal or renocardiac or cardiorenal or reno-cardiac or reno-cardiac) adj3 syndrome?) or (dyspnea? adj2 paroxysmal) or (asthma adj2 cardiac) or (cardiac adj2 edema?) or preserved ejection fraction or HFpEF or normal ejection fraction or HFnEF).mp. | 219957 |
| 4 | exp pulmonary disease, chronic obstructive/ or (COPD or COAD or (chronic adj4 obstructi$ adj4 (pulmonary or bronchopulmonary or bronchiti? or airway or airflow or lung or respiratory)) or chronic bronchitis or ((centrilobular or centriacinar or panlobular or panacinar or focal or pulmonary) adj3 emphysema?)).mp. | 87373 |
| 5 | exp renal insufficiency, chronic/ or (((end-stage or end stage or endstage$ or failure) adj4 (kidney or renal) adj4 (disease or insufficien$ or chronic)) or CKD or ESRD or ESKD or (frasier adj2 syndrome?) or ((glomerular filtration rate/ or kidney diseases/ or renal insufficiency/ or ((kidney or renal) adj4 (disease or insufficien$ or failure)) or "mild to moderate kidney disease" or MMKD or hemodialys?s or haemodialys?s or hemodiafiltrat$ or haemodiafiltrat$ or dialys?s or dialytic) adj6 (chronic disease/ or chronic))).mp. | 160921 |
| 6 | Diabetes Mellitus/ or Diabetes Mellitus, Type 1/ or Diabetes Mellitus, Type 2/ or (((diabetes or diabetic$) and (insulin depend$ or insulin treat$ or intensive insulin$ or non insulin depend$ or noninsulin depend$ or non-insulin-depend$ or non insulin-depend$ or maturity onset$ or maturity-onset or adult onset$ or adult-onset or slow onset$ or slow-onset or autoimmune)) or diabetes or diabetic$ or dm1 or IDDM or dm 1 or t1d$ or dm type 1 or type 1 diabet$ or dm type I or type one diabet$ or type I diabet$ or dm2 or NIDDM or dm 2 or t2d$ or dm type 2 or type 2 diabet$ or dm type II or type two diabet$ or type II diabet$ or MODY).mp. | 623213 |
| 7 | 3 or 4 or 5 or 6 | 1031552 |
| 8 | 1 and 2 and 7 | 1025 |
| 9 | (adolescent/ or exp child/ or exp infant/ or (adolescent$ or child$ or schoolchild$ or infant$ or girl$ or boy$ or teen? or teenager$ or youth$ or pediatr$ or paediatr$ or puber$).tw.) not (exp adult/ or (adult$ or man or men or woman or women).tw.) | 1961859 |
| 10 | 8 not 9 | 983 |

**EMBASE SEARCH**

**Performed by:** Joanna Bielecki (joanna.bielecki@theta.utoronto.ca)

**Results sent to:** Nida Shahid <nida.shahid@theta.utoronto.ca>

**Date:** 27 September 2018

**Search name:** "eHealth-Telecare-Coaching-Four-Chronic-Cond-SR-Embase FINAL", Nida Shahid Sept 2018

**Database(s):** **Embase Classic+Embase**1947 to 2018 September 26

**Search Strategy:**

| **#** | **Searches** | **Results** |
| --- | --- | --- |
| 1 | telemedicine/ or remote consultation/ or telehealth/ or teleconsultation/ or telemonitoring/ or teletherapy/ or (mhealth or telehealth or ehealth or e-health or (mobile adj2 health) or ((monitoring or consultation) adj2 remote) or (mobile adj health) or telemedicine or telenursing or telehomecare or telecare or teleconsultation? or telemonitor$ or telemanagement or telesurveillance or evisit or e-visit).mp. | 46029 |
| 2 | exp health care planning/ or health care organization/ or exp health care system/ or exp hospital organization/ or national health organization/ or national health service/ or health services research/ or health service/ or exp health care/ or public health service/ or needs assessment/ or comparative effectiveness/ or public health/ or health care need/ or health care facility/ or (((health or healthcare or "health care") adj3 (implementation? or adoption? or coalition? or rationing or reform? or priorit$ or resource? or "appropriateness review?")) or ((health or healthcare or health-care or "health care") adj3 service? adj3 (evaluation? or "comparative effectiveness" or research or need? or demand? or "needs assessment?" or "educational need?" or national$)) or ((organization$ or management) adj3 case adj3 stud$) or (comparative adj3 effectiveness adj3 research) or (("single payer" or single-payer) adj4 (system? or plan?)) or (medicine adj3 (socialized or state)) or (medically adj4 underserved adj4 area?) or (physician? adj4 shortage) or ((health or healthcare or health-care or "health care") adj4 (plan$ or program$ or intervention?) adj4 (support? or subsid$ or grant$ or organization? or center? or centre? or council? or agenc$ or guideline? or recommendation? or technical$ or national$ or regional$ or state? or province? or comprehensiv$ or communit$ or population-based or facilit$ or system?))).mp. | 5347095 |
| 3 | exp heart failure/ or (((heart or myocardial or cardiac or diastolic or systolic) adj3 (fail$ or decompensation or dysfunction$)) or ((cardio renal or cardio-renal or renocardiac or cardiorenal or reno-cardiac or reno-cardiac) adj3 syndrome?) or (dyspnea? adj2 paroxysmal) or (asthma adj2 cardiac) or (cardiac adj2 edema?) or preserved ejection fraction or HFpEF or normal ejection fraction or HFnEF).mp. | 543048 |
| 4 | exp chronic obstructive lung disease/ or (COPD or COAD or (chronic adj4 obstructi$ adj4 (pulmonary or bronchopulmonary or bronchiti? or airway or airflow or lung or respiratory)) or chronic bronchitis or ((centrilobular or centriacinar or panlobular or panacinar or focal or pulmonary) adj3 emphysema?)).mp. | 161257 |
| 5 | exp chronic kidney failure/ or (((end-stage or end stage or endstage$ or failure) adj4 (kidney or renal) adj4 (disease or insufficien$ or chronic)) or CKD or ESRD or ESKD or (frasier adj2 syndrome?) or ((glomerulus filtration rate/ or kidney disease/ or kidney failure/ or ((kidney or renal) adj4 (disease? or insufficien$ or failure)) or "mild to moderate kidney disease" or MMKD or hemodialys?s or haemodialys?s or hemodiafiltrat$ or haemodiafiltrat$ or dialys?s or dialytic) adj6 (chronic disease/ or chronic))).mp. | 222827 |
| 6 | Diabetes Mellitus/ or insulin dependent diabetes mellitus/ or non insulin dependent diabetes mellitus/ or (((diabetes or diabetic$) and (insulin depend$ or insulin treat$ or intensive insulin$ or non insulin depend$ or noninsulin depend$ or non-insulin-depend$ or non insulin-depend$ or maturity onset$ or maturity-onset or adult onset$ or adult-onset or slow onset$ or slow-onset or autoimmune)) or diabetes or diabetic$ or dm1 or IDDM or dm 1 or t1d$ or dm type 1 or type 1 diabet$ or dm type I or type one diabet$ or type I diabet$ or dm2 or NIDDM or dm 2 or t2d$ or dm type 2 or type 2 diabet$ or dm type II or type two diabet$ or type II diabet$ or MODY).mp. | 1051693 |
| 7 | 3 or 4 or 5 or 6 | 1824745 |
| 8 | 1 and 2 and 7 | 6073 |
| 9 | (adolescent/ or exp child/ or (adolescent$ or child$ or schoolchild$ or infant$ or girl$ or boy$ or teen? or teenager$ or youth$ or pediatr$ or paediatr$ or puber$).tw.) not (exp adult/ or exp aged/ or middle aged/ or (adult$ or man or men or woman or women).tw.) | 2491920 |
| 10 | 8 not 9 | 5857 |

**CINAHL SEARCH**

**Performed by:** Joanna Bielecki (joanna.bielecki@theta.utoronto.ca)

**Results sent to:** Nida Shahid <nida.shahid@theta.utoronto.ca>

**Date:** Thursday, September 27, 2018 5:40:52 PM

**Search name:** eHealth-Telecare-Coaching-Four-Chronic-Cond-SR-CINAHL- FINAL; Nida Shahid Sept2018

**Database(s):** Interface - EBSCOhost Research Databases; Advanced Search; Database – CINAHL

**Search Strategy:**

| **#** | **Query** | **Results** |
| --- | --- | --- |
| S24 | S22 not S23 | 285 |
| S23 | ((MH "Adolescence") OR (MH "Child") OR (MH "Infant") OR (MH "Infant, Newborn") OR TI ( (adolescent* or child* or schoolchild* or infant* or girl* or boy* or teen? or teenager* or youth* or pediatr* or paediatr* or puber*) ) OR AB ( (adolescent* or child* or schoolchild* or infant* or girl* or boy* or teen? or teenager* or youth* or pediatr* or paediatr* or puber*) ) OR SU ( (adolescent* or child* or schoolchild* or infant* or girl* or boy* or teen? or teenager* or youth* or pediatr* or paediatr* or puber*) ) ) NOT ( (MH "Adult+") OR TI ( (adult* or man or men or woman or women) ) OR AB ( (adult* or man or men or woman or women) ) OR SU ( (adult* or man or men or woman or women))) | 402,479 |
| S22 | S1 AND S2 AND S21 | 304 |
| S21 | S3 OR S4 OR S5 OR S6 OR S18 OR S19 OR S20 | 205,338 |
| S20 | TX (((diabetes or diabetic*) and (insulin depend* or insulin treat* or intensive insulin* or non insulin depend* or noninsulin depend* or non-insulin-depend* or non insulin-depend* or maturity onset* or maturity-onset or adult onset* or adult-onset or slow onset* or slow-onset or autoimmune)) or diabetes or diabetic* or dm1 or IDDM or dm 1 or t1d* or dm type 1 or type 1 diabet* or dm type I or type one diabet* or type I diabet* or dm2 or NIDDM or dm 2 or t2d* or dm type 2 or type 2 diabet* or dm type II or type two diabet* or type II diabet* or MODY) | 139,131 |
| S19 | (MH "Diabetes Mellitus") OR (MH "Diabetes Mellitus, Type 2") OR (MH "Diabetes Mellitus, Type 1") | 76,438 |
| S18 | S7 OR S8 OR S17 | 23,075 |
| S17 | S13 N6 S16 | 21,263 |
| S16 | S14 OR S15 | 174,419 |
| S15 | TX chronic | 174,419 |
| S14 | (MH "Chronic Disease") | 36,643 |
| S13 | S9 OR S10 OR S11 OR S12 | 51,748 |
| S12 | TX (((kidney or renal) N4 (disease or insufficien* or failure)) or "mild to moderate kidney disease" or MMKD or hemodialys?s or haemodialys?s or hemodiafiltrat* or haemodiafiltrat* or dialys?s or dialytic) | 50,691 |
| S11 | (MH "Renal Insufficiency") | 3,242 |
| S10 | (MH "Kidney Diseases") | 7,720 |
| S9 | (MH "Glomerular Filtration Rate") | 4,162 |
| S8 | TX (((end-stage or end stage or endstage* or failure) N4 (kidney or renal) N4 (disease or insufficien* or chronic)) or CKD or ESRD or ESKD or (frasier N2 syndrome)) | 16,838 |
| S7 | (MH "Renal Insufficiency, Chronic+") | 13,391 |
| S6 | TX (COPD or COAD or (chronic N4 obstructi* N4 (pulmonary or bronchopulmonary or bronchiti? or airway or airflow or lung or respiratory)) or chronic bronchitis or ((centrilobular or centriacinar or panlobular or panacinar or focal or pulmonary) N3 emphysema)) | 15,830 |
| S5 | (MH "Pulmonary Disease, Chronic Obstructive+") | 10,480 |
| S4 | TX (((heart or myocardial or cardiac or diastolic or systolic) N3 (fail* or decompensation or dysfunction*)) or ((cardio renal or cardio-renal or renocardiac or cardiorenal or reno-cardiac or reno-cardiac) N3 syndrome) or (dyspnea N2 paroxysmal) or (asthma N2 cardiac) or (cardiac N2 edema) or preserved ejection fraction or HFpEF or normal ejection fraction or HFnEF) | 39,863 |
| S3 | (MH "Heart Failure+") | 22,677 |
| S2 | (MH "Health Facility Planning+") OR (MH "State Health Plans") OR (MH "Health Systems Agencies") OR (MH "Health and Welfare Planning") OR (MH "Health Resource Allocation") OR (MH "Health Services Needs and Demand+") OR (MH "National Health Programs+") OR (MH "Allied Health Organizations") OR (MH "Health Maintenance Organizations") OR (MH "Health Care Reform") OR (MH "Health Resource Utilization") OR (MH "Health Services Research") OR (MH "Needs Assessment") OR TX (((health or healthcare or "health care") N3 (implementation* or adoption* or coalition* or rationing or reform* or priorit* or resource* or "appropriateness review*")) or ((health or healthcare or health-care or "health care") N3 service* N3 (evaluation* or "comparative effectiveness" or research or need* or demand* or "needs assessment*" or "educational need*" or national*)) or ((organization* or management) N3 case N3 stud*) or (comparative N3 effectiveness N3 research) or (("single payer" or single-payer) N4 (system* or plan*)) or (medicine N3 (socialized or state)) or (medically N4 underserved N4 area*) or (physician* N4 shortage) or ((health or healthcare or health-care or "health care") N4 (plan* or program* or intervention*) N4 (support* or subsid* or grant* or organization* or center* or centre* or council* or agenc* or guideline* or recommendation* or technical* or national* or regional* or state* or province* or comprehensiv* or communit* or population-based or facilit* or system*))) | 212,505 |
| S1 | (MH "Telemedicine") OR (MH "Telehealth") OR (MH "Remote Consultation") OR TX (mhealth or telehealth or ehealth or e-health or (mobile N2 health) or ((monitoring or consultation) N2 remote) or (mobile N health) or telemedicine or telenursing or telehomecare or telecare or teleconsultation* or telemonitor* or telemanagement or telesurveillance or evisit or e-visit) | 16,122 |

**COCHRANE SEARCH**

**Performed by:** Joanna Bielecki (joanna.bielecki@theta.utoronto.ca)

**Results sent to:** Nida Shahid <nida.shahid@theta.utoronto.ca>

**Date:** 19/10/2018 11:31:27

**Search name:** e-Health-Telecare-Coaching-ChronicDisease_Cochrane – FINAL, Nida Shahid - Oct 2018.

**Database(s):** Cochrane Database of Systematic Reviews, Issue 10 of 12, October 2018

Cochrane Central Register of Controlled Trials, Issue 9 of 12, September 2018

**Search Strategy:**


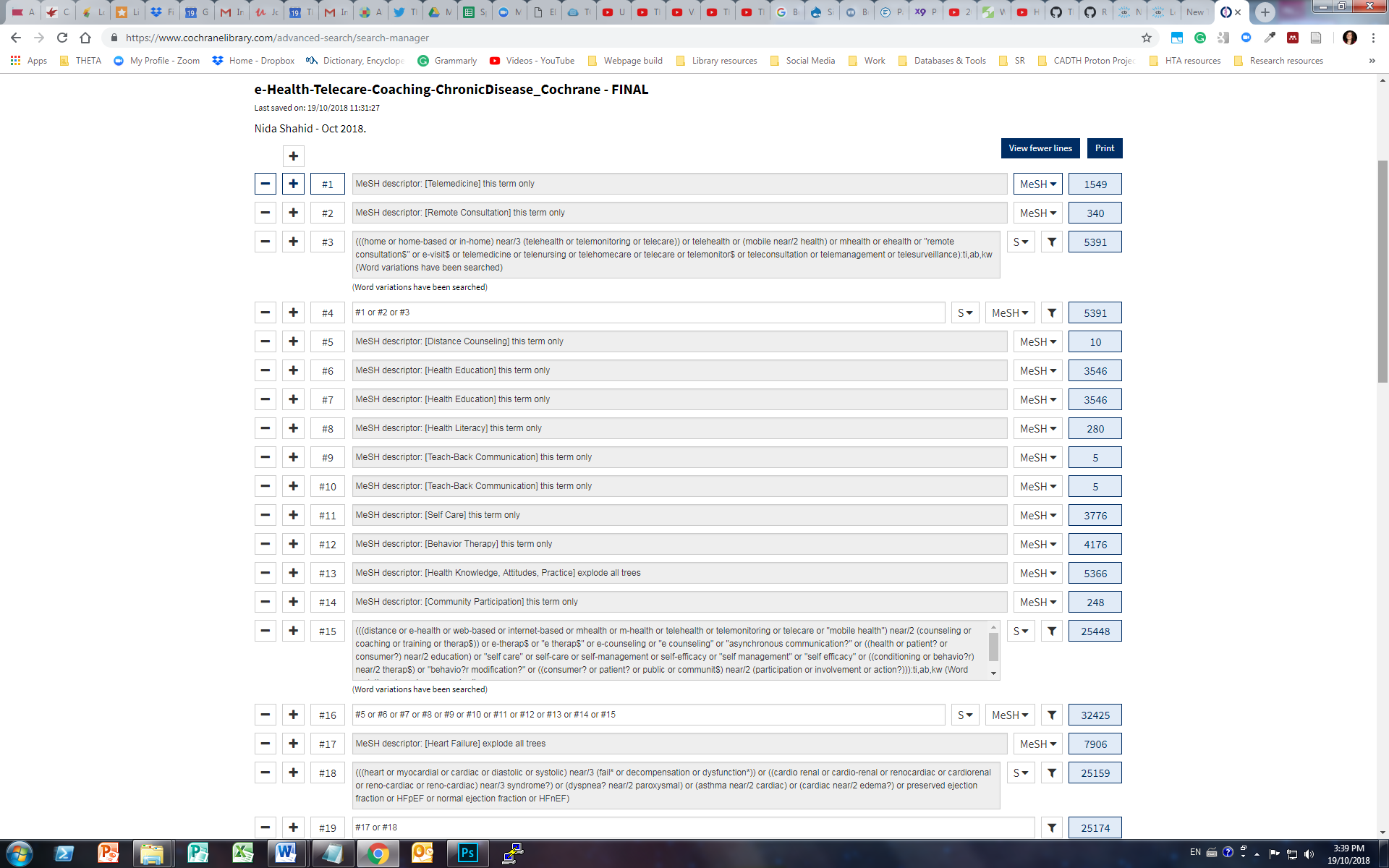


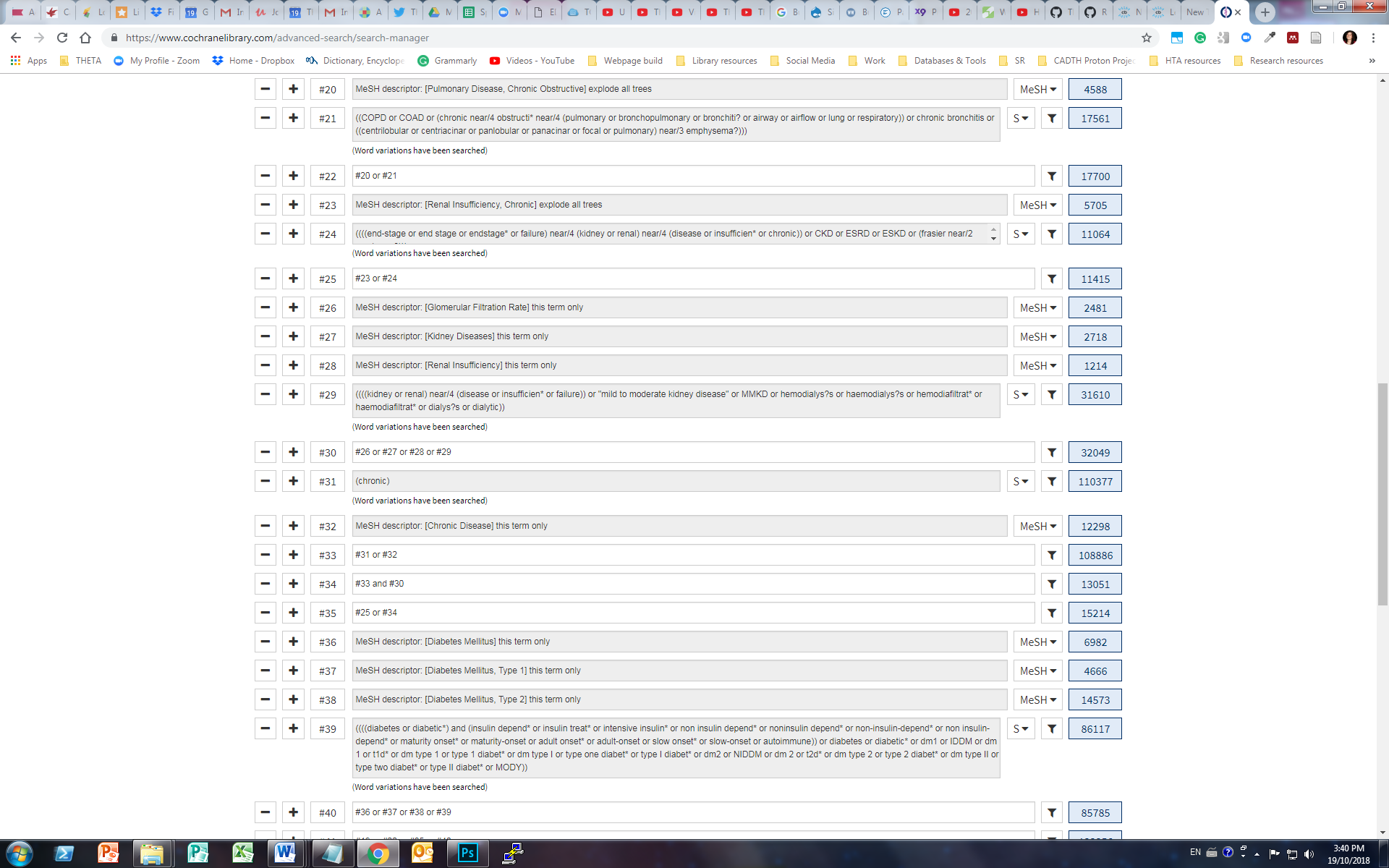


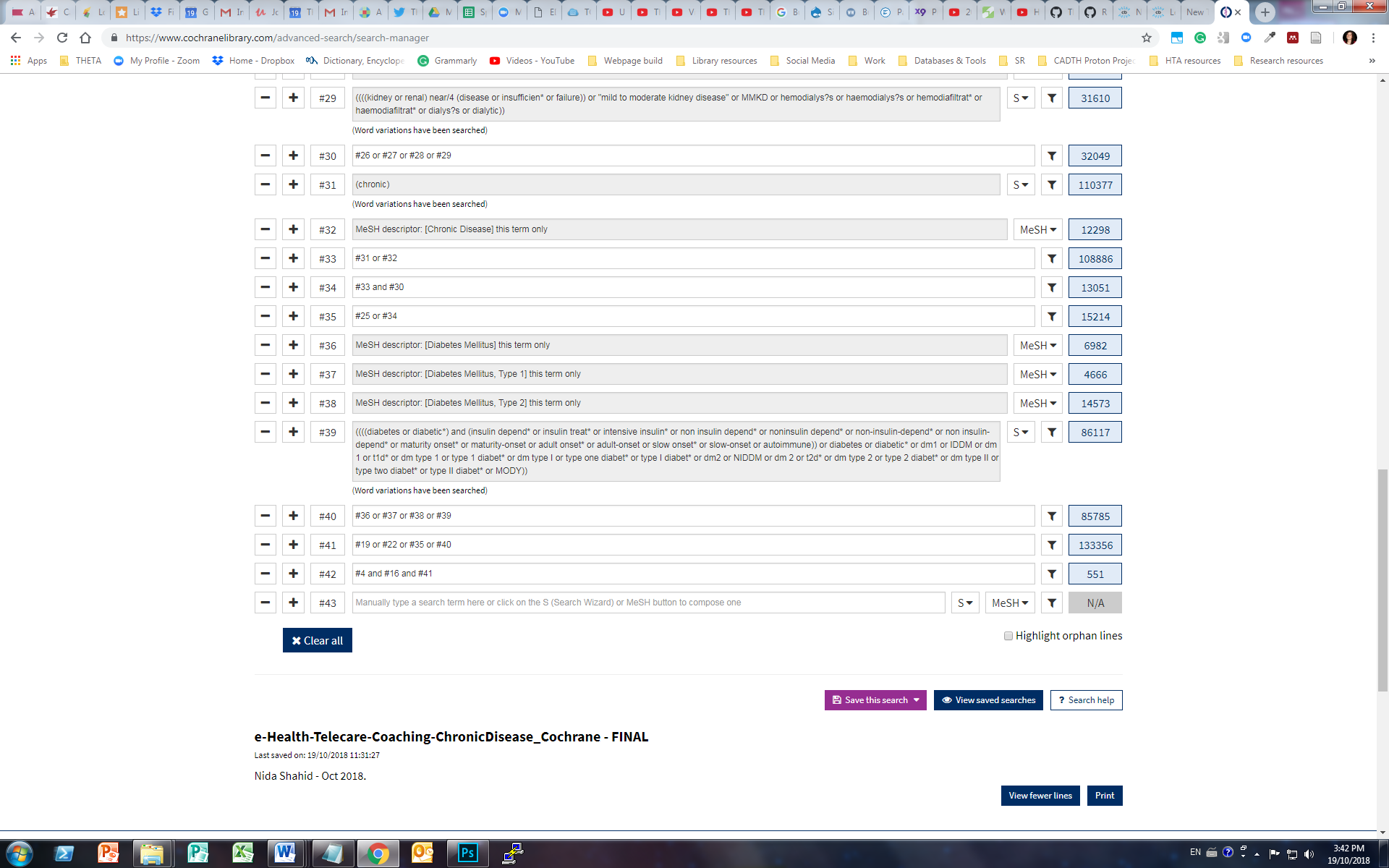


**PsycINFO SEARCH**

**Performed by:** Joanna Bielecki (joanna.bielecki@theta.utoronto.ca)

**Results sent to:** Nida Shahid <nida.shahid@theta.utoronto.ca>

**Date:** 27 September 2018

**Search name:** " eHealth-Telecare-Coaching-Four-Chronic-Cond-SR-PsycINFO FINAL ", Nida Shahid Sept 2018

**Database(s):** **PsycINFO**1806 to September Week 4 2018

**Search Strategy:**

Database(s): 
Search Strategy:

| **#** | **Searches** | **Results** |
| --- | --- | --- |
| 1 | telemedicine/ or (mhealth or telehealth or ehealth or e-health or (mobile adj2 health) or ((monitoring or consultation) adj2 remote) or (mobile adj health) or telemedicine or telenursing or telehomecare or telecare or teleconsultation? or telemonitor$ or telemanagement or telesurveillance or evisit or e-visit).mp. | 6886 |
| 2 | Health Care Delivery/ or Health Care Services/ or Health Care Reform/ or Health Care Policy/ or Program Development/ or Health Service Needs/ or Needs Assessment/ or Public Health/ or Community Health/ or Health Disparities.mp. or (((health or healthcare or "health care") adj3 (implementation? or adoption? or coalition? or rationing or reform? or priorit$ or resource? or "appropriateness review?")) or ((health or healthcare or health-care or "health care") adj3 service? adj3 (evaluation? or "comparative effectiveness" or research or need? or demand? or "needs assessment?" or "educational need?" or national$)) or ((organization$ or management) adj3 case adj3 stud$) or (comparative adj3 effectiveness adj3 research) or (("single payer" or single-payer) adj4 (system? or plan?)) or (medicine adj3 (socialized or state)) or (medically adj4 underserved adj4 area?) or (physician? adj4 shortage) or ((health or healthcare or health-care or "health care") adj4 (plan$ or program$ or intervention?) adj4 (support? or subsid$ or grant$ or organization? or center? or centre? or council? or agenc$ or guideline? or recommendation? or technical$ or national$ or regional$ or state? or province? or comprehensiv$ or communit$ or population-based or facilit$ or system?))).mp. [mp=title, abstract, heading word, table of contents, key concepts, original title, tests & measures] | 124976 |
| 3 | exp Heart Disorders/ or (((heart or myocardial or cardiac or diastolic or systolic) adj3 (fail$ or decompensation or dysfunction$)) or ((cardio renal or cardio-renal or renocardiac or cardiorenal or reno-cardiac or reno-cardiac) adj3 syndrome?) or (dyspnea? adj2 paroxysmal) or (asthma adj2 cardiac) or (cardiac adj2 edema?) or preserved ejection fraction or HFpEF or normal ejection fraction or HFnEF).mp. | 15348 |
| 4 | exp Chronic Obstructive Pulmonary Disease/ or (COPD or COAD or (chronic adj4 obstructi$ adj4 (pulmonary or bronchopulmonary or bronchiti? or airway or airflow or lung or respiratory)) or chronic bronchitis or ((centrilobular or centriacinar or panlobular or panacinar or focal or pulmonary) adj3 emphysema?)).mp. | 2661 |
| 5 | (((end-stage or end stage or endstage$ or failure) adj4 (kidney or renal) adj4 (disease or insufficien$ or chronic)) or CKD or ESRD or ESKD or (frasier adj2 syndrome?)).mp. | 1712 |
| 6 | (kidney diseases/ or (((kidney or renal) adj4 (disease or insufficien$ or failure)) or "mild to moderate kidney disease" or MMKD or hemodialys?s or haemodialys?s or hemodiafiltrat$ or haemodiafiltrat$ or dialys?s or dialytic).mp.) adj6 ("Chronicity (Disorders)"/ or chronic.mp.) | 2074 |
| 7 | 5 or 6 | 2838 |
| 8 | Diabetes Mellitus/ or Type 2 Diabetes/ or (((diabetes or diabetic$) and (insulin depend$ or insulin treat$ or intensive insulin$ or non insulin depend$ or noninsulin depend$ or non-insulin-depend$ or non insulin-depend$ or maturity onset$ or maturity-onset or adult onset$ or adult-onset or slow onset$ or slow-onset or autoimmune)) or diabetes or diabetic$ or dm1 or IDDM or dm 1 or t1d$ or dm type 1 or type 1 diabet$ or dm type I or type one diabet$ or type I diabet$ or dm2 or NIDDM or dm 2 or t2d$ or dm type 2 or type 2 diabet$ or dm type II or type two diabet$ or type II diabet$ or MODY).mp. | 29265 |
| 9 | 3 or 4 or 7 or 8 | 47628 |
| 10 | 1 and 2 and 9 | 140 |
| 11 | ((adolescent$ or child$ or schoolchild$ or infant$ or girl$ or boy$ or teen? or teenager$ or youth$ or pediatr$ or paediatr$ or puber$) not (adult$ or man or men or woman or women)).ti,id,tc. | 573421 |
| 12 | 10 not 11 | 133 |
