# Supplementary material for: A realist review of factors critical for the implementation of eHealth in chronic disease management
Source: BMC Health Serv Res. 2025 Apr 2;25:496. doi: 10.1186/s12913-025-12361-0 (PMC11966836; doi:10.1186/s12913-025-12361-0)
Supplement: Supplementary file 2 — Supplementary Material 2. [file 12913_2025_12361_MOESM2_ESM.docx]

## Appendix 2. Search Strategy and Syntax (2018-22)

**Towards better understanding of factors critical for the implementation and/or adoption of initiatives using technology in heart failure, COPD, chronic kidney disease, and diabetes management – realist review – UPDATE (02 May 2022)**

**Performed by:** Joanna Bielecki (joanna.bielecki@gmail.com)

**Results sent to:** Nida Shahid [nida.shahid@theta.utoronto.ca](mailto:nida.shahid@theta.utoronto.ca)

**Date:**

04 May 2022 (Ovid MEDLINE, Embase & PsychINFO)

10 May 2022 (Cochrane Library, CINAHL Ebsco)

**Databases searched:**

Ovid MEDLINE

Ovid Embase

Ovid PsycINFO

Cochrane Library Wiley

CINAHL Ebsco

**MULTIFILE SEARCH – Ovid MEDLINE, Embase & PsychINFO**

Search saved as:

eHealth-Telecare-Coaching-Four-Chronic-Cond-SR-Medline FINAL - update May 2022

eHealth-Telecare-Coaching-Four-Chronic-Cond-SR-Embase FINAL - update May 2022

eHealth-Telecare-Coaching-Four-Chronic-Cond-SR-PsycINFO FINAL - update May 2022

**Search run on:** 04 May 2022

Database(s): **Ovid MEDLINE: Epub Ahead of Print, In-Process & Other Non-Indexed Citations, Ovid MEDLINE® Daily and Ovid MEDLINE®**1946-Present**, Embase Classic+Embase**1947 to 2022 May 03**, APA PsycInfo**1806 to April Week 4 2022

Search Strategy:

| **#** | **Searches** | **Results** |
| --- | --- | --- |
| 1 | telemedicine/ or remote consultation/ or (((home or home-based or in-home) adj3 (telehealth or telemonitoring or telecare)) or telehealth or (mobile adj health) or mhealth or ehealth or "remote consultation$" or e-visit$ or telemedicine or telenursing or telehomecare or telecare or telemonitor$ or teleconsultation or telemanagement or telesurveillance).ti,ab. | 130857 |
| 2 | Distance Counseling/ or Health Education/ or Consumer Health Information/ or Health Literacy/ or Teach-Back Communication/ or patient education as topic/ or self care/ or behavior therapy/ or health knowledge, attitudes, practice/ or consumer participation/ or (((distance or e-health or web-based or internet-based or mhealth or m-health or telehealth or telemonitoring or telecare or "mobile health") adj2 (counseling or coaching or training or therap$)) or e-therap$ or "e therap$" or e-counseling or "e counseling" or "asynchronous communication?" or ((health or patient? or consumer?) adj2 education) or "self care" or self-care or self-management or self-efficacy or "self management" or "self efficacy" or ((conditioning or behavio?r) adj2 therap$) or "behavio?r modification?" or ((consumer? or patient? or public or communit$) adj2 (participation or involvement or action?))).ti,ab. | 1365644 |
| 3 | exp heart failure/ or (((heart or myocardial or cardiac or diastolic or systolic) adj3 (fail$ or decompensation or dysfunction$)) or ((cardio renal or cardio-renal or renocardiac or cardiorenal or reno-cardiac or reno-cardiac) adj3 syndrome?) or (dyspnea? adj2 paroxysmal) or (asthma adj2 cardiac) or (cardiac adj2 edema?) or preserved ejection fraction or HFpEF or normal ejection fraction or HFnEF).mp. | 996063 |
| 4 | exp pulmonary disease, chronic obstructive/ or (COPD or COAD or (chronic adj4 obstructi$ adj4 (pulmonary or bronchopulmonary or bronchiti? or airway or airflow or lung or respiratory)) or chronic bronchitis or ((centrilobular or centriacinar or panlobular or panacinar or focal or pulmonary) adj3 emphysema?)).mp. | 323999 |
| 5 | [exp renal insufficiency, chronic/ or (((end-stage or end stage or endstage$ or failure) adj4 (kidney or renal) adj4 (disease or insufficien$ or chronic)) or CKD or ESRD or ESKD or (frasier adj2 syndrome?) or ((glomerular filtration rate/ or kidney diseases/ or renal insufficiency/ or ((kidney or renal) adj4 (disease or insufficien$ or failure)) or "mild to moderate kidney disease" or MMKD or hemodialys?s or haemodialys?s or hemodiafiltrat$ or haemodiafiltrat$ or dialys?s or dialytic) adj6 (chronic disease/ or chronic))).mp.] | 0 |
| 6 | Diabetes Mellitus/ or Diabetes Mellitus, Type 1/ or Diabetes Mellitus, Type 2/ or (((diabetes or diabetic$) and (insulin depend$ or insulin treat$ or intensive insulin$ or non insulin depend$ or noninsulin depend$ or non-insulin-depend$ or non insulin-depend$ or maturity onset$ or maturity-onset or adult onset$ or adult-onset or slow onset$ or slow-onset or autoimmune)) or diabetes or diabetic$ or dm1 or IDDM or dm 1 or t1d$ or dm type 1 or type 1 diabet$ or dm type I or type one diabet$ or type I diabet$ or dm2 or NIDDM or dm 2 or t2d$ or dm type 2 or type 2 diabet$ or dm type II or type two diabet$ or type II diabet$ or MODY).mp. | 2191196 |
| 7 | 3 or 4 or 5 or 6 | 3341799 |
| 8 | 1 and 2 and 7 | 4420 |
| 9 | (adolescent/ or exp child/ or exp infant/ or (adolescent$ or child$ or schoolchild$ or infant$ or girl$ or boy$ or teen? or teenager$ or youth$ or pediatr$ or paediatr$ or puber$).tw.) not (exp adult/ or (adult$ or man or men or woman or women).tw.) | 6018399 |
| 10 | 8 not 9 | 4249 |
| 11 | limit 10 to dt="20180901-20221231" [MEDLINE update results for Sept 2018 - Current] | 3261 |
| 12 | telemedicine/ or remote consultation/ or telehealth/ or teleconsultation/ or telemonitoring/ or teletherapy/ or (mhealth or telehealth or ehealth or e-health or (mobile adj2 health) or ((monitoring or consultation) adj2 remote) or (mobile adj health) or telemedicine or telenursing or telehomecare or telecare or teleconsultation? or telemonitor$ or telemanagement or telesurveillance or evisit or e-visit).mp. | 178703 |
| 13 | exp health care planning/ or health care organization/ or exp health care system/ or exp hospital organization/ or national health organization/ or national health service/ or health services research/ or health service/ or exp health care/ or public health service/ or needs assessment/ or comparative effectiveness/ or public health/ or health care need/ or health care facility/ or (((health or healthcare or "health care") adj3 (implementation? or adoption? or coalition? or rationing or reform? or priorit$ or resource? or "appropriateness review?")) or ((health or healthcare or health-care or "health care") adj3 service? adj3 (evaluation? or "comparative effectiveness" or research or need? or demand? or "needs assessment?" or "educational need?" or national$)) or ((organization$ or management) adj3 case adj3 stud$) or (comparative adj3 effectiveness adj3 research) or (("single payer" or single-payer) adj4 (system? or plan?)) or (medicine adj3 (socialized or state)) or (medically adj4 underserved adj4 area?) or (physician? adj4 shortage) or ((health or healthcare or health-care or "health care") adj4 (plan$ or program$ or intervention?) adj4 (support? or subsid$ or grant$ or organization? or center? or centre? or council? or agenc$ or guideline? or recommendation? or technical$ or national$ or regional$ or state? or province? or comprehensiv$ or communit$ or population-based or facilit$ or system?))).mp. | 8554408 |
| 14 | exp heart failure/ or (((heart or myocardial or cardiac or diastolic or systolic) adj3 (fail$ or decompensation or dysfunction$)) or ((cardio renal or cardio-renal or renocardiac or cardiorenal or reno-cardiac or reno-cardiac) adj3 syndrome?) or (dyspnea? adj2 paroxysmal) or (asthma adj2 cardiac) or (cardiac adj2 edema?) or preserved ejection fraction or HFpEF or normal ejection fraction or HFnEF).mp. | 996063 |
| 15 | exp chronic obstructive lung disease/ or (COPD or COAD or (chronic adj4 obstructi$ adj4 (pulmonary or bronchopulmonary or bronchiti? or airway or airflow or lung or respiratory)) or chronic bronchitis or ((centrilobular or centriacinar or panlobular or panacinar or focal or pulmonary) adj3 emphysema?)).mp. | 323999 |
| 16 | [exp chronic kidney failure/ or (((end-stage or end stage or endstage$ or failure) adj4 (kidney or renal) adj4 (disease or insufficien$ or chronic)) or CKD or ESRD or ESKD or (frasier adj2 syndrome?) or ((glomerulus filtration rate/ or kidney disease/ or kidney failure/ or ((kidney or renal) adj4 (disease? or insufficien$ or failure)) or "mild to moderate kidney disease" or MMKD or hemodialys?s or haemodialys?s or hemodiafiltrat$ or haemodiafiltrat$ or dialys?s or dialytic) adj6 (chronic disease/ or chronic))).mp.] | 0 |
| 17 | Diabetes Mellitus/ or insulin dependent diabetes mellitus/ or non insulin dependent diabetes mellitus/ or (((diabetes or diabetic$) and (insulin depend$ or insulin treat$ or intensive insulin$ or non insulin depend$ or noninsulin depend$ or non-insulin-depend$ or non insulin-depend$ or maturity onset$ or maturity-onset or adult onset$ or adult-onset or slow onset$ or slow-onset or autoimmune)) or diabetes or diabetic$ or dm1 or IDDM or dm 1 or t1d$ or dm type 1 or type 1 diabet$ or dm type I or type one diabet$ or type I diabet$ or dm2 or NIDDM or dm 2 or t2d$ or dm type 2 or type 2 diabet$ or dm type II or type two diabet$ or type II diabet$ or MODY).mp. | 2191196 |
| 18 | 14 or 15 or 16 or 17 | 3341799 |
| 19 | 12 and 13 and 18 | 15199 |
| 20 | (adolescent/ or exp child/ or (adolescent$ or child$ or schoolchild$ or infant$ or girl$ or boy$ or teen? or teenager$ or youth$ or pediatr$ or paediatr$ or puber$).tw.) not (exp adult/ or exp aged/ or middle aged/ or (adult$ or man or men or woman or women).tw.) | 5804143 |
| 21 | 19 not 20 | 14611 |
| 22 | limit 21 to dc="20180901-20221231" [Embase update results for Sept 2018 - Current] | 4875 |
| 23 | telemedicine/ or (mhealth or telehealth or ehealth or e-health or (mobile adj2 health) or ((monitoring or consultation) adj2 remote) or (mobile adj health) or telemedicine or telenursing or telehomecare or telecare or teleconsultation? or telemonitor$ or telemanagement or telesurveillance or evisit or e-visit).mp. | 169847 |
| 24 | Health Care Delivery/ or Health Care Services/ or Health Care Reform/ or Health Care Policy/ or Program Development/ or Health Service Needs/ or Needs Assessment/ or Public Health/ or Community Health/ or Health Disparities.mp. or (((health or healthcare or "health care") adj3 (implementation? or adoption? or coalition? or rationing or reform? or priorit$ or resource? or "appropriateness review?")) or ((health or healthcare or health-care or "health care") adj3 service? adj3 (evaluation? or "comparative effectiveness" or research or need? or demand? or "needs assessment?" or "educational need?" or national$)) or ((organization$ or management) adj3 case adj3 stud$) or (comparative adj3 effectiveness adj3 research) or (("single payer" or single-payer) adj4 (system? or plan?)) or (medicine adj3 (socialized or state)) or (medically adj4 underserved adj4 area?) or (physician? adj4 shortage) or ((health or healthcare or health-care or "health care") adj4 (plan$ or program$ or intervention?) adj4 (support? or subsid$ or grant$ or organization? or center? or centre? or council? or agenc$ or guideline? or recommendation? or technical$ or national$ or regional$ or state? or province? or comprehensiv$ or communit$ or population-based or facilit$ or system?))).mp. [mp=ti, ab, ot, nm, hw, fx, kf, ox, px, rx, ui, sy, tn, dm, mf, dv, dq, tc, id, tm] | 1604730 |
| 25 | exp Heart Disorders/ or (((heart or myocardial or cardiac or diastolic or systolic) adj3 (fail$ or decompensation or dysfunction$)) or ((cardio renal or cardio-renal or renocardiac or cardiorenal or reno-cardiac or reno-cardiac) adj3 syndrome?) or (dyspnea? adj2 paroxysmal) or (asthma adj2 cardiac) or (cardiac adj2 edema?) or preserved ejection fraction or HFpEF or normal ejection fraction or HFnEF).mp. | 1886113 |
| 26 | exp Chronic Obstructive Pulmonary Disease/ or (COPD or COAD or (chronic adj4 obstructi$ adj4 (pulmonary or bronchopulmonary or bronchiti? or airway or airflow or lung or respiratory)) or chronic bronchitis or ((centrilobular or centriacinar or panlobular or panacinar or focal or pulmonary) adj3 emphysema?)).mp. | 324131 |
| 27 | (((end-stage or end stage or endstage$ or failure) adj4 (kidney or renal) adj4 (disease or insufficien$ or chronic)) or CKD or ESRD or ESKD or (frasier adj2 syndrome?)).mp. | 419960 |
| 28 | (kidney diseases/ or (((kidney or renal) adj4 (disease or insufficien$ or failure)) or "mild to moderate kidney disease" or MMKD or hemodialys?s or haemodialys?s or hemodiafiltrat$ or haemodiafiltrat$ or dialys?s or dialytic).mp.) adj6 ("Chronicity (Disorders)"/ or chronic.mp.) | 431030 |
| 29 | 27 or 28 | 524454 |
| 30 | Diabetes Mellitus/ or Type 2 Diabetes/ or (((diabetes or diabetic$) and (insulin depend$ or insulin treat$ or intensive insulin$ or non insulin depend$ or noninsulin depend$ or non-insulin-depend$ or non insulin-depend$ or maturity onset$ or maturity-onset or adult onset$ or adult-onset or slow onset$ or slow-onset or autoimmune)) or diabetes or diabetic$ or dm1 or IDDM or dm 1 or t1d$ or dm type 1 or type 1 diabet$ or dm type I or type one diabet$ or type I diabet$ or dm2 or NIDDM or dm 2 or t2d$ or dm type 2 or type 2 diabet$ or dm type II or type two diabet$ or type II diabet$ or MODY).mp. | 2191196 |
| 31 | 25 or 26 or 29 or 30 | 4559237 |
| 32 | 23 and 24 and 31 | 2833 |
| 33 | ((adolescent$ or child$ or schoolchild$ or infant$ or girl$ or boy$ or teen? or teenager$ or youth$ or pediatr$ or paediatr$ or puber$) not (adult$ or man or men or woman or women)).ti,id,tc. | 3791013 |
| 34 | 32 not 33 | 2752 |
| 35 | 34 and (201809* or 201810* or 201811* or 201812* or 2019* or 2020* or 2021* or 2022*).up. [PsychINFO update results for Sept 2018 - Current] | 1789 |
| 36 | 11 use ppez [MEDLINE results] | 626 |
| 37 | 22 use emczd [Embase results] | 4775 |
| 38 | 35 use psyc15,psyc16,psyc17,psyc18 [PsychINFO results] | 62 |
| 39 | 36 or 37 or 38 | 5463 |
| **40** | **remove duplicates from 39** | **4935** |

EndNote deduplication process: **14 duplicates** removed


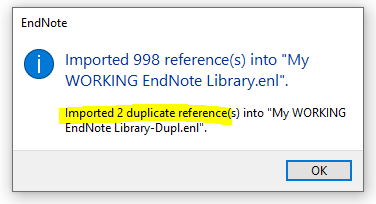


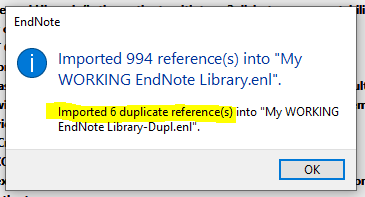


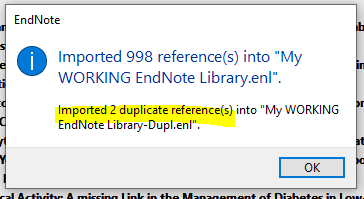


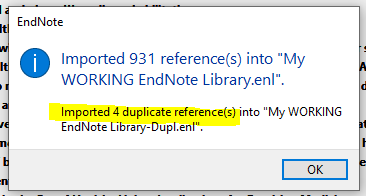


**Cochrane Library Search**

Search Name: e-Health-Telecare-Coaching-ChronicDisease_Cochrane - FINAL-update May 2022

Date Run: 11/05/2022 01:29:21

Comment: Nida Shahid - 10 May 2022

**ID Search Hits**

#1 MeSH descriptor: [Telemedicine] this term only 2687

#2 MeSH descriptor: [Remote Consultation] this term only 387

#3 (((home or home-based or in-home) near/3 (telehealth or telemonitoring or telecare)) or telehealth or (mobile near/2 health) or mhealth or ehealth or "remote consultation$" or e-visit$ or telemedicine or telenursing or telehomecare or telecare or telemonitor$ or teleconsultation or telemanagement or telesurveillance):ti,ab,kw (Word variations have been searched) 11241

#4 #1 or #2 or #3 11241

#5 MeSH descriptor: [Distance Counseling] this term only 23

#6 MeSH descriptor: [Health Education] this term only 4186

#7 MeSH descriptor: [Health Education] this term only 4186

#8 MeSH descriptor: [Health Literacy] this term only 447

#9 MeSH descriptor: [Teach-Back Communication] this term only 10

#10 MeSH descriptor: [Teach-Back Communication] this term only 10

#11 MeSH descriptor: [Self Care] this term only 4353

#12 MeSH descriptor: [Behavior Therapy] this term only 4890

#13 MeSH descriptor: [Health Knowledge, Attitudes, Practice] explode all trees 6306

#14 MeSH descriptor: [Community Participation] this term only 300

#15 (((distance or e-health or web-based or internet-based or mhealth or m-health or telehealth or telemonitoring or telecare or "mobile health") near/2 (counseling or coaching or training or therap$)) or e-therap$ or "e therap$" or e-counseling or "e counseling" or "asynchronous communication?" or ((health or patient? or consumer?) near/2 education) or "self care" or self-care or self-management or self-efficacy or "self management" or "self efficacy" or ((conditioning or behavio?r) near/2 therap$) or "behavio?r modification?" or ((consumer? or patient? or public or communit$) near/2 (participation or involvement or action?))):ti,ab,kw (Word variations have been searched) 79177

#16 #5 or #6 or #7 or #8 or #9 or #10 or #11 or #12 or #13 or #14 or #15 85996

#17 MeSH descriptor: [Heart Failure] explode all trees 10390

#18 (((heart or myocardial or cardiac or diastolic or systolic) near/3 (fail* or decompensation or dysfunction*)) or ((cardio renal or cardio-renal or renocardiac or cardiorenal or reno-cardiac or reno-cardiac) near/3 syndrome?) or (dyspnea? near/2 paroxysmal) or (asthma near/2 cardiac) or (cardiac near/2 edema?) or preserved ejection fraction or HFpEF or normal ejection fraction or HFnEF) 39307

#19 #17 or #18 39307

#20 MeSH descriptor: [Pulmonary Disease, Chronic Obstructive] 1 tree(s) exploded 6283

#21 ((COPD or COAD or (chronic near/4 obstructi* near/4 (pulmonary or bronchopulmonary or bronchiti? or airway or airflow or lung or respiratory)) or chronic bronchitis or ((centrilobular or centriacinar or panlobular or panacinar or focal or pulmonary) near/3 emphysema?))) (Word variations have been searched) 25341

#22 #20 or #21 25330

#23 MeSH descriptor: [Renal Insufficiency, Chronic] 2 tree(s) exploded 412

#24 ((((end-stage or end stage or endstage* or failure) near/4 (kidney or renal) near/4 (disease or insufficien* or chronic)) or CKD or ESRD or ESKD or (frasier near/2 syndrome?))) (Word variations have been searched) 19262

#25 #23 or #24 19035

#26 MeSH descriptor: [Glomerular Filtration Rate] this term only 2964

#27 MeSH descriptor: [Kidney Diseases] this term only 3658

#28 MeSH descriptor: [Renal Insufficiency] this term only 1644

#29 ((((kidney or renal) near/4 (disease or insufficien* or failure)) or "mild to moderate kidney disease" or MMKD or hemodialys?s or haemodialys?s or hemodiafiltrat* or haemodiafiltrat* or dialys?s or dialytic)) (Word variations have been searched) 50776

#30 #26 or #27 or #28 or #29 50434

#31 (chronic) (Word variations have been searched) 168228

#32 MeSH descriptor: [Chronic Disease] this term only 13674

#33 #31 or #32 166160

#34 #33 and #30 21026

#35 #25 or #34 25547

#36 MeSH descriptor: [Diabetes Mellitus] this term only 10546

#37 MeSH descriptor: [Diabetes Mellitus, Type 1] this term only 6016

#38 MeSH descriptor: [Diabetes Mellitus, Type 2] this term only 19841

#39 ((((diabetes or diabetic*) and (insulin depend* or insulin treat* or intensive insulin* or non insulin depend* or noninsulin depend* or non-insulin-depend* or non insulin-depend* or maturity onset* or maturity-onset or adult onset* or adult-onset or slow onset* or slow-onset or autoimmune)) or diabetes or diabetic* or dm1 or IDDM or dm 1 or t1d* or dm type 1 or type 1 diabet* or dm type I or type one diabet* or type I diabet* or dm2 or NIDDM or dm 2 or t2d* or dm type 2 or type 2 diabet* or dm type II or type two diabet* or type II diabet* or MODY)) (Word variations have been searched) 130696

#40 #36 or #37 or #38 or #39 130150

#41 #19 or #22 or #35 or #40 201968

#42 #4 and #16 and #41 1124

**#43 #4 and #16 and #41 Cochrane Library: publication date Between Sep 2018 and Dec 2022 535**

**CINAHL Search**

Database - CINAHL Plus with Full Text

Date run: Wednesday, May 11, 2022 5:10:00 AM

Last Run Via Interface - EBSCOhost Research Databases

Search Screen - Advanced Search

Limiters - Published Date: 20180901-20221231
Search modes - Boolean/Phrase

| Wednesday, May 11, 2022 5:10:00 AM |
| --- |

| **#** | **Query** | **Results** |
| --- | --- | --- |
| **S25** | **S22 not S23 (Limiters - Published Date: 20180901-20221231)** | **1,153** |
| S24 | S22 not S23 | 4,394 |
| S23 | ((MH "Adolescence") OR (MH "Child") OR (MH "Infant") OR (MH "Infant, Newborn") OR TI ( (adolescent* or child* or schoolchild* or infant* or girl* or boy* or teen? or teenager* or youth* or pediatr* or paediatr* or puber*) ) OR AB ( (adolescent* or child* or schoolchild* or infant* or girl* or boy* or teen? or teenager* or youth* or pediatr* or paediatr* or puber*) ) OR SU ( (adolescent* or child* or schoolchild* or infant* or girl* or boy* or teen? or teenager* or youth* or pediatr* or paediatr* or puber*) ) ) NOT ( (MH "Adult+") OR TI ( (adult* or man or men or woman or women) ) OR AB ( (adult* or man or men or woman or women) ) OR SU ( (adult* or man or men or woman or women))) | 833,124 |
| S22 | S1 AND S2 AND S21 | 4,632 |
| S21 | S3 OR S4 OR S5 OR S6 OR S18 OR S19 OR S20 | 591,080 |
| S20 | TX (((diabetes or diabetic*) and (insulin depend* or insulin treat* or intensive insulin* or non insulin depend* or noninsulin depend* or non-insulin-depend* or non insulin-depend* or maturity onset* or maturity-onset or adult onset* or adult-onset or slow onset* or slow-onset or autoimmune)) or diabetes or diabetic* or dm1 or IDDM or dm 1 or t1d* or dm type 1 or type 1 diabet* or dm type I or type one diabet* or type I diabet* or dm2 or NIDDM or dm 2 or t2d* or dm type 2 or type 2 diabet* or dm type II or type two diabet* or type II diabet* or MODY) | 409,824 |
| S19 | (MH "Diabetes Mellitus") OR (MH "Diabetes Mellitus, Type 2") OR (MH "Diabetes Mellitus, Type 1") | 156,631 |
| S18 | S7 OR S8 OR S17 | 84,908 |
| S17 | S13 N6 S16 | 77,950 |
| S16 | S14 OR S15 | 557,561 |
| S15 | TX chronic | 557,561 |
| S14 | (MH "Chronic Disease") | 70,063 |
| S13 | S9 OR S10 OR S11 OR S12 | 160,791 |
| S12 | TX (((kidney or renal) N4 (disease or insufficien* or failure)) or "mild to moderate kidney disease" or MMKD or hemodialys?s or haemodialys?s or hemodiafiltrat* or haemodiafiltrat* or dialys?s or dialytic) | 157,663 |
| S11 | (MH "Renal Insufficiency") | 6,841 |
| S10 | (MH "Kidney Diseases") | 16,727 |
| S9 | (MH "Glomerular Filtration Rate") | 11,394 |
| S8 | TX (((end-stage or end stage or endstage* or failure) N4 (kidney or renal) N4 (disease or insufficien* or chronic)) or CKD or ESRD or ESKD or (frasier N2 syndrome)) | 50,710 |
| S7 | (MH "Renal Insufficiency, Chronic+") | 31,409 |
| S6 | TX (COPD or COAD or (chronic N4 obstructi* N4 (pulmonary or bronchopulmonary or bronchiti? or airway or airflow or lung or respiratory)) or chronic bronchitis or ((centrilobular or centriacinar or panlobular or panacinar or focal or pulmonary) N3 emphysema)) | 52,415 |
| S5 | (MH "Pulmonary Disease, Chronic Obstructive+") | 21,847 |
| S4 | TX (((heart or myocardial or cardiac or diastolic or systolic) N3 (fail* or decompensation or dysfunction*)) or ((cardio renal or cardio-renal or renocardiac or cardiorenal or reno-cardiac or reno-cardiac) N3 syndrome) or (dyspnea N2 paroxysmal) or (asthma N2 cardiac) or (cardiac N2 edema) or preserved ejection fraction or HFpEF or normal ejection fraction or HFnEF) | 127,921 |
| S3 | (MH "Heart Failure+") | 47,344 |
| S2 | (MH "Health Facility Planning+") OR (MH "State Health Plans") OR (MH "Health Systems Agencies") OR (MH "Health and Welfare Planning") OR (MH "Health Resource Allocation") OR (MH "Health Services Needs and Demand+") OR (MH "National Health Programs+") OR (MH "Allied Health Organizations") OR (MH "Health Maintenance Organizations") OR (MH "Health Care Reform") OR (MH "Health Resource Utilization") OR (MH "Health Services Research") OR (MH "Needs Assessment") OR TX (((health or healthcare or "health care") N3 (implementation* or adoption* or coalition* or rationing or reform* or priorit* or resource* or "appropriateness review*")) or ((health or healthcare or health-care or "health care") N3 service* N3 (evaluation* or "comparative effectiveness" or research or need* or demand* or "needs assessment*" or "educational need*" or national*)) or ((organization* or management) N3 case N3 stud*) or (comparative N3 effectiveness N3 research) or (("single payer" or single-payer) N4 (system* or plan*)) or (medicine N3 (socialized or state)) or (medically N4 underserved N4 area*) or (physician* N4 shortage) or ((health or healthcare or health-care or "health care") N4 (plan* or program* or intervention*) N4 (support* or subsid* or grant* or organization* or center* or centre* or council* or agenc* or guideline* or recommendation* or technical* or national* or regional* or state* or province* or comprehensiv* or communit* or population-based or facilit* or system*))) | 517,681 |
| S1 | (MH "Telemedicine") OR (MH "Telehealth") OR (MH "Remote Consultation") OR TX (mhealth or telehealth or ehealth or e-health or (mobile N2 health) or ((monitoring or consultation) N2 remote) or (mobile N health) or telemedicine or telenursing or telehomecare or telecare or teleconsultation* or telemonitor* or telemanagement or telesurveillance or evisit or e-visit) | 63,089 |
