# Supplementary material for: A realist review of factors critical for the implementation of eHealth in chronic disease management
Source: BMC Health Serv Res. 2025 Apr 2;25:496. doi: 10.1186/s12913-025-12361-0 (PMC11966836; doi:10.1186/s12913-025-12361-0)
Supplement: Supplementary file 3 — Supplementary Material 3. [file 12913_2025_12361_MOESM3_ESM.docx]

## Appendix 3. Broad and specific themes of actions and responses facilitating ehealth implementation in CDM.

| **STUDY ID** | **Action Main Category** | **Action Sub-Category** | **Response Main Category** | **Response Sub-Category** |
| --- | --- | --- | --- | --- |
| 1 | Program features | Ease of use | Patient | Self-management |
| 1 | Access | Provider | Patient | Sense of security |
| 7 | Patient | Training | Patient | Ease of use |
| 8 | Program features | Ease of use | Patient | Ease of use, reliability |
| 10 | Care | Clinical judgement | Program | Reach |
| 10 | Provider | Relevant expertise | Care | Improved care |
| 11 | Program features | Ease of use; Perceived usefulness, increased autonomy | Patient | Ease |
| 11 | Integration | Within broader self-management approaches | Patient | Acceptance |
| 11 | Patient | Program reach | Program | Cost (Return) |
| 13 | Communication | Follow up | Patient | Positive experience |
| 16 | Communication | Feedback | Provider | Access to information |
| 19 | Provider | Training | Provider | Relationship building |
| 21 | Program features | Ease of use | Patient | Positive attitude |
| 21 | Patient | Program experience | Patient | Sense of security |
| 21 | Motivation | Reduced workload | Provider | Perceived usefulness |
| 22 | Program features | Ease of use | Patient | Satisfaction |
| 22 | User | Program experience | User | Ease of use |
| 23 | Patient | Support; Communication | Patient | Acceptance |
| 26 | Motivation | Comfort, convenience | Patient | Ease of use |
| 26 | Communication | Continuity | Care | Education |
| 26 | User | Reduced travel costs | Program | Reach |
| 26 | Communication | Communication | User | Relationship building |
| 27 | Access | Information | Patient | Motivation |
| 27 | Program use | Program use | Patient | Self-management |
| 27 | Program use | Program use | User | Communication |
| 27 | Program use | Program use | User | Perceived usefulness |
| 28 | Patient | Program experience | Patient | Self-management |
| 28 | Patient | Program benefits | Patient | Self-empowerment |
| 34 | Patient | Program experience | Patient | Communication; Positive experience |
| 34 | Program use | Program use | Patient | Self-management |
| 35 | Program features | Ease of use | User | Positive attitude |
| 35 | Patient | Support | User | Positive attitude |
| 35 | Access | Information | Patient | Self-management |
| 37 | Patient | Program experience | Patient | Satisfaction |
| 37 | Access | Care | Patient | Satisfaction |
| 38 | Program features | Ease of use | Care | Improved care |
| 38 | Patient | Reassurance | Patient | Satisfaction |
| 38 | User | Relationship building | Patient | Satisfaction |
| 38 | User | Relationship building | Care | Improved care |
| 41 | Patient | Program experience | Patient | Self-management |
| 41 | Patient | Program experience | Patient | Self-awareness |
| 41 | Patient | Program experience | Patient | Reassurance |
| 41 | Access | Information | Care | Improved care |
| 41 | User | Program benefits | User | Motivation |
| 43 | Access | Information | User | Perceived usefulness |
| 43 | Access | Information | User | Reassurance |
| 49 | Motivation | Relationship building | Provider | Perceived usefulness |
| 49 | Other | Legislation | Care | Sustainable services |
| 51 | Program features | Ease of use | Care | Integrated care |
| 51 | Program use | Program use | Care | Improved care |
| 51 | Care | Preparation | Care | Improved care |
| 51 | Program features | Various strategies, technical modalities | User | Relationship building |
| 51 | SDOH factors | Health: early stage disease | Program | Uptake |
| 54 | Motivation | Improved health | Program | Cost (Effectiveness) |
| 55 | Motivation | Reduced travel, healthcare costs | User | Acceptance |
| 55 | Program features | Various strategies, technical modalities | Provider | Perceived usefulness |
| 55 | User | Relationship building | User | Communication |
| 55 | Communication | Quality | User | Relationship building |
| 55 | Communication | Follow up | User | Acceptance |
| 60 | Program features | Ease of use | Provider | Acceptance |
| 60 | Communication | Collaboration | Provider | Acceptance |
| 60 | Provider | Training | Provider | Acceptance |
| 60 | Program features | Flexible processes | Provider | Acceptance |
| 60 | User | Perception of safety | Provider | Acceptance |
| 60 | Integration | Within routine care | Provider | Acceptance |
| 60 | Communication | Feedback | Provider | Acceptance |
| 60 | Other | Leadership | Provider | Acceptance |
| 62 | Motivation | Support and reassurance | Patient | Satisfaction |
| 62 | SDOH factors | Health: applicability | Care | Sustainable services |
| 62 | User | Relationship building | Care | Improved care |
| 63 | Motivation | Self-management | Program | Uptake |
| 63 | Access | Care | Program | Uptake |
| 63 | Program features | Ease of use | Program | Uptake |
| 63 | User | Perceived value | Program | Uptake |
| 65 | Communication | Relationship building | Program | Uptake |
| 67 | Motivation | Ease of use;  Perceived usefulness | Patient | Satisfaction |
| 67 | SDOH factors | Income level | User | Satisfaction |
| 71 | Information | Information | Patient | Self-management |
| 71 | Care | Provider led | Patient | Self-empowerment |
| 71 | Program features | Remote communication | User | Communication |
| 71 | Program features | Contact Frequency | Patient | Reassurance |
| 71 | Program use | Program use | Patient | Self-empowerment |
| 73 | User | Type of role | User | Perceived usefulness |
| 73 | Access | Information | Combination: Patient and Care | Self-management; Improved care |
| 74 | SDOH factors | Context and needs | User | Perceived usefulness |
| 74 | Program features | Ease of use | Program | Adherence |
| 74 | Motivation | Patient-level | Program | Use |
| 74 | Patient | Reassurance | Patient | Self-empowerment |
| 74 | Communication | Open | Patient | Satisfaction; Motivation |
| 74 | Program features | Administrative support | User | Performance |
| 74 | Integration | Across providers and programs | Program | Objectives |
| 74 | Other | Legislation | Program | Implementation |
| 76 | Program features | Equipment cost | Program | Cost |
| 77 | Program features | Processes | Program | Reach |
| 77 | Program features | Processes | Program | Resource use |
| 77 | Communication | Collaboration | Program | Awareness |
| 77 | User | Relationship building | Program | Cost (Financial Security) |
| 79 | Motivation | Empathy, Acceptance | Program | Uptake |
| 79 | Program features | Personalization | Provider | Role |
| 79 | Program features | Program reach | Program | Uptake |
| 79 | Program features | Personalization | Program | Uptake |
| 79 | Patient | Self-awareness | User | Reassurance |
| 79 | Patient | Self-awareness | Patient | Motivation |
| 87 | Information | Written and verbal | Patient | Training |
| 94 | Patient | Program experience | Patient | Confidence |
| 94 | Program features | Remote communication | Care | Improved care |
| 94 | Program use | Program use | Provider | Time management |
| 94 | Program use | Program use | Provider | Reassurance |
| 94 | Program use | Program use | Provider | Job satisfaction |
| 99 | Program features | Ease of use | User | Positive attitude, Confidence |
| 99 | Program features | Ability to contact provider | Patient | Confidence |
| 99 | Provider | Reassurance | Patient | Self-management |
| 100 | Motivation | Relationship building, Trust | User | Experience |
| 100 | Program use | Program use | Care | Improved care |
| 100 | Care | Planning | Care | Integrated care |
| 103 | User | Training | Program | Adoption |
| 103 | User | Technical support | Program | Adoption |
| 103 | SDOH factors | Social support | Program | Adoption |
| 118 | SDOH factors | Social support | Patient | Self-management |
| 118 | SDOH factors | Social support | Patient | Self-management |
| 118 | Program features | Disease Education | Care | Improved care |
| 124 | Program features | Disease Education; SDOH (Social Support) | Patient | Overcome SDOH barrier |
| 170 | SDOH factors | Geographical distance | Patient | Willingness to pay |
| 219 | Program features | Program purpose | Program | Implementation |
| 219 | User | Relationship building | User | Acceptance |
| 219 | Program features | Adaptive, flexible processes | Program | Adoption |
| 219 | Program features | Reflexive monitoring | Program | Feasability |
| 221 | Motivation | Empowerment, confidence Program: easy to use | Program | Adoption |
| 221 | Program features | Usability | Program | Adoption |
| 221 | Program features | System readiness | Program | Adoption |
| 222 | Program use | Program use | Patient | Self-management |
| 222 | Communication | Provider led | User | Perceived usefulness |
| 222 | Access | Information | Patient | Self-management |
| 222 | Motivation | Values and self-identity | Program | Adoption |
| 222 | Motivation | Personal motivation | Program | Cost (Staffing) |
| 267 | SDOH factors | Generational differences | User | Preferences |
| 303 | Program features | Staffing | Program | Cost |
| 303 | Program features | Equipment and transmission fees | Program | Cost (Sustainability) |
| 305 | Program features | Personnel Time | Program | Cost (Staffing) |
| 312 | Program use | Program use | Patient | Self-management |
| 312 | Access | Care | Patient | Self-empowerment |
| 312 | Program features | Personalization | User | Engagement |
| 312 | User | Training | User | Perceived usefulness |
| 315 | User | Perceived value | Program | Implementation |
| 315 | Program features | Low cost | Program | Implementation |
| 340 | Program features | Ease of use | Care | Integrated care |
| 340 | Program features | Personalization | Provider | Role |
| 340 | Program features | Patient reminders | Program | Use |
| 340 | Program features | Disease Education | Care | Integrated care |
| 340 | Program features | Patient Led | User | Engagement |
| 349 | Program use | Program use | Patient | Self-management |
| 353 | Information | Access | Care | Improved care |
| 353 | Program use | Program use | Patient | Self-management |
| 357 | Patient | Program experience | Patient | Satisfaction |
| 357 | Access | Care | User | Perceived usefulness |
| 357 | Access | Care | Other | Other: System utilization |
| 357 | Program use | Program use | Provider | Time management |
| 357 | Program features | Personalization, Various Modalities | Program | Adoption |
| 371 | User | Relationship building | User | Perceived usefulness |
| 378 | Access | Access to care provider | Patient | Perceived usefulness |
| 378 | Access | Access to inforation | Care | Continuity of care |
| 380 | Program features | Ease of use | Patient | Self-awareness |
| 380 | Program use | Program use | Care | Improved care |
| 380 | Program use | Program use | Patient | Self-management |
| 380 | Program use | Program use | Patient | Security |
| 380 | Program use | Program use | Patient | Security |
| 380 | User | Technical support | User | Confidence |
| 386 | Motivation | Willingess and desire to learn | Care | Access to care |
| 386 | User | Perceived value | Care | Access to care |
| 386 | Program features | Ease of use | Care | Access to care |
| 386 | Program features | Personalization | Care | Access to care |
| 394 | Motivation | Self-management | Program | Acceptance |
| 394 | Program features | Remote communication | User | Flexibility |
| 394 | User | Security | User | Security |
| 394 | Program features | Various strategies, technical modalities | User | Perceived usefulness |
