# Supplementary material for: A realist review of factors critical for the implementation of eHealth in chronic disease management
Source: BMC Health Serv Res. 2025 Apr 2;25:496. doi: 10.1186/s12913-025-12361-0 (PMC11966836; doi:10.1186/s12913-025-12361-0)
Supplement: Supplementary file 4 — Supplementary Material 4. [file 12913_2025_12361_MOESM4_ESM.docx]

## Appendix 4. Broad and specific themes of actions and responses hindering ehealth implementation in CDM.

| **Study ID** | **Action Main Category** | **Action Sub-Category** | **Response Main Category** | **Response Sub-Category** |
| --- | --- | --- | --- | --- |
| 11 | Program use | Increased involvement (patient communication, need for information) | Provider | Increased workload |
| 13 | Program use | Increased involvement (data entry, reminders) | User | Negative experience |
| 16 | Organizational | Mundane components, Conflicting information | Provider | Increased workload |
| 19 | User | Inexperience | User | Perceived usability |
| 19 | Technology | Technical problems | Organizational | Time loss |
| 19 | Training | Lack of training | Care | Poor care |
| 19 | User | Poor attitude | Intervention | Target enrollment |
| 23 | Technology | Technical problems | Patient | Negative experience |
| 23 | Organizational | Changes | User | Reduced learning |
| 26 | Technology | Technical problems | Provider, Care | Negative experience, care disruption |
| 38 | Organizational | Conflicting information | Organizational | Operational problems |
| 38 | Technology | Technical problems | Provider | Negative experience |
| 38 | Organizational | Conflicting information | Provider | Negative experience |
| 41 | Cost | Cost | Implementation | Implementation barrier |
| 41 | Program use | Increased involvement (clinical workload, workflow changes) | Implementation | Implementation barrier |
| 41 | Organizational | Operational problems | Implementation | Implementation barrier |
| 41 | Intervention | System dependency | Implementation | Implementation barrier |
| 43 | Technology | Equipment | Organizational | Operational problems |
| 43 | Technology | Technical problems | User | Negative experience |
| 49 | Organizational | Operational problems | Implementation | Implementation barrier |
| 55 | SDOH | Distance | User | Perceived usability |
| 60 | Organizational | Disruption | Provider | Poor staff acceptance |
| 60 | User | Negative impact on patient staff relationship | Provider | Poor staff acceptance |
| 60 | User | Perceived usefulness | Provider | Poor staff acceptance |
| 60 | User | Low authonomy | Provider | Poor staff acceptance |
| 60 | Technology | Technical problems | Provider | Poor staff acceptance |
| 60 | Technology | User friendliness concerns | Provider | Poor staff acceptance |
| 60 | User | Concerns (Reliability) | Provider | Poor staff acceptance |
| 60 | User | Low literacy (Technical) | Provider | Poor staff acceptance |
| 60 | Technology | Installationa challenges | Provider | Poor staff acceptance |
| 60 | Organizational | Low efficiency | Provider | Poor staff acceptance |
| 60 | User | Concerns (Safety) | Provider | Poor staff acceptance |
| 60 | Organizational | Poor change management | Provider | Poor staff acceptance |
| 62 | Program use | Increased involvement (additional workload) | Provider | Negative experience |
| 63 | Technology | Technical problems | Implementation | Uptake |
| 63 | User | Perceived usefulness | Implementation | Uptake |
| 63 | SDOH | Distance | Implementation | Uptake |
| 63 | User | Anxiety | Implementation | Uptake |
| 63 | Technology | Technical problems | Implementation | Uptake |
| 65 | Organizational | Operational problems | Provider | Increased workload |
| 65 | Intervention | Target population | Intervention | Program effectiveness |
| 65 | Cost | Payment models | User | Acceptance |
| 68 | Organizational | Disruption | User | Negative experience |
| 74 | Technology | Technical problems | Implementation | Implementation |
| 74 | User | Time constraints | Implementation | Implementation |
| 74 | SDOH | Distance | Implementation | Implementation |
| 74 | Intervention | Access | Intervention | Program effectiveness |
| 77 | Cost | Funding | Intervention | Program improvements |
| 77 | Cost | Funding | Implementation | Implementation |
| 93 | SDOH | Less education | Implementation | Implementation barrier |
| 93 | User | Low literacy (Technical) | Implementation | Implementation barrier |
| 93 | User | Medication non-adherence | Implementation | Implementation barrier |
| 93 | User | Desire for in-person contact | Implementation | Implementation barrier |
| 93 | User | Low literacy (Health) | Implementation | Implementation barrier |
| 93 | Technology | Expensive technology | Implementation | Implementation barrier |
| 93 | SDOH | Limited internet access | Implementation | Implementation barrier |
| 93 | Intervention | Lack of tailoring to patient needs | Implementation | Implementation barrier |
| 93 | Intervention | Lack of reliability | Implementation | Implementation barrier |
| 93 | Intervention | Irrelevant content | Implementation | Implementation barrier |
| 93 | Organizational | Lack of workflow integration | Implementation | Implementation barrier |
| 93 | Cost | Lack of insurnace reimbursement | Implementation | Implementation barrier |
| 93 | Cost | High cost of intervention | Implementation | Implementation barrier |
| 117 | Technology | Technical problems | User | Acceptance |
| 118 | Program use | Increased involvement | Organizational | Care disruption |
| 118 | SDOH | Language | Care | Poor care |
| 118 | SDOH | Religion | Implementation | Adherence |
| 170 | SDOH | Age | Cost | Willingness to pay |
| 171 | Training | Training | Cost | Costs |
| 221 | User | Perceived usefulness | Program use | Adoption |
| 221 | Organizational | System dependency, | Program use | Adoption |
| 221 | User | Increased involvement (workload), roles | Program use | Adoption |
| 222 | User | Perceived usefulness | User | Perceived usability |
| 312 | SDOH | Language | Implementation | Usability |
| 315 | Program use | Increased involvement (workload), limited time | Implementation | Implementation barrier |
| 315 | Training | Lack of training | Implementation | Implementation barrier |
| 353 | Program use | Increased incolvement (workload) | Implementation | Adoption |
| 353 | User | Communication | User | Negative experience |
| 353 | Program use | Increased involvement (workload) | Provider | Negative experience |
| 371 | Program use | Increased involvement | Organizational | Increased workload |
| 378 | Program use | Increased involvement (workload), limited time | Provider | Increased workload |
| 380 | User | Perceived usefulness | Implementation | Uptake |
| 380 | Intervention | Target population | User | Lack of confidence |
| 380 | User | Perceived usefulness | Implementation | Uptake |
| 380 | SDOH | Distance | Implementation | Implementation barrier |
| 386 | SDOH | Age | Implementation | Access |
| 386 | Training | Lack of training | Implementation | Access |
| 386 | Cost | Funding | Implementation | Access |
| 386 | Training | Lack of training | Implementation | Uptake |
| 386 | SDOH | Distance | Implementation | Implementation barrier |
| 386 | User | Lack of trust | User | Demotivation |
| 394 | Training | Lack of training | Implementation | Implementation barrier |
