# Supplementary material for: A realist review of factors critical for the implementation of eHealth in chronic disease management
Source: BMC Health Serv Res. 2025 Apr 2;25:496. doi: 10.1186/s12913-025-12361-0 (PMC11966836; doi:10.1186/s12913-025-12361-0)
Supplement: Supplementary file 5 — Supplementary Material 5. [file 12913_2025_12361_MOESM5_ESM.docx]

## Appendix 5. Facilitating CMOs

# CMO 1

| Study ID | Care Setting | Geography | Intervention | Implementation Stage | Provider -Role | Provider-Experience | Patient-Social | Chronic Condition | Patient-Health | Action Main | Action Sub | Response Main | Response Sub | Primary Outcome | Anticipated Outcome | Unanticipated Outcome | Outcome Success | Confidence in CMO |
| --- | --- | --- | --- | --- | --- | --- | --- | --- | --- | --- | --- | --- | --- | --- | --- | --- | --- | --- |
| 1 | (Primary) university based and veterans health administration | -- | -- | -- | Physicians, nurses | -- | Different social backgrounds | HF | HF for min. 6 months | Program features | Ease of use | Patient | Self-management | Feasibility, Efficacy | Usability | Improved self-management and perceived usefulness | Yes | 1 |
| 8 | (Primary) University based outpatient clinic | -- | Training/Instructions Initial intake process before enrollment | -- | -- | -- | -- | HF | -- | Program features | Ease of use | Patient | Ease of use, reliability | Acceptance, Feasibility, Reliability | Reliability, Usability, Feasibility | Perceived usefulness | Yes | 0 |
| 11 | -- | -- | -- | -- | -- | -- | -- | Diabetes | -- | Program features | Ease of use; Perceived usefulness, increased autonomy | Patient | Ease | Implementation factors | Program Acceptance (by patients) | Cost return | Yes | 0 |
| 21 | (Primary) Family health network | (Mixed) Semi-rural network | Provide broad range of care |  | Family MDs, nurses | Providing care to ~10,000 patients | Mostly retired | Combination (Diabetes, Cardiovascular disease, COPD) | Most frail | Program features | Ease of use | Patient | Positive attitude | Feasibility, Efficacy | Program Acceptance | Not reported | Yes | 1 |
| 22 | (Tertiary) European hospitals (multiple) | -- | -- | Beyond Pilot | -- | Suitable managerial skills, structure and knowledge about HF and ICD for routine care. | -- | HF | with implants | Program features | Ease of use | Patient | Satisfaction | Implementation | Patient and Physician Satisfaction | Ease of use over time, MD perspective data review in remote follow up as exhaustive as in person | Yes | 1 |
| 380 | -- | -- | -- | - | - | -- | -- | Combination  (CKD, COPD, HF, Diabetes) | -- | Program features | Ease of use | Patient | \Self-awareness | Patient beliefs, Attitudes, Expectations and Experiences | Not reported | Not reported | Yes | 1 |
| 38 | -- | -- | Varying models used | -- | Community respiratory physiotherapists, community nurses, practice admin staff in consultation with GPs | -- | -- | COPD | -- | Program features | Ease of use | Care | Improved care | Views | Impact on telemonitoring | Not reported | Unclear | 1 |
| 51 | (Tertiary) Multiple regional hospitals | Urban area | -- | -- | -- | Certified diabetes case managers | -- | Diabetes | -- | Program features | Ease of use | Care | Integrated care | Experiences, Acceptance, Perceived effectiveness | Acceptance, Perceived effectiveness | Not reported | Yes | 1 |
| 340 | -- | -- | -- | Beyond Pilot | Clinical Commission Group, membership bodies | -- | -- | Diabetes | -- | Program features | Ease of use | Care | Integrated care | Implementation factors | Not reported | Flexible approach is necessary for implementation | Unclear | 1 |
| 386 | -- | -- | -- | -- | -- | -- | -- | Combination  (HF, Cardiovascular disease) | -- | Program features | Ease of use | Care | Access to care | Engagement factors | Not reported | Not reported | Unclear | 1 |
| 63 | -- | -- | -- | -- | -- | -- | -- | Combination  (COPD, HF) | -- | Program features | Ease of use | Program | Uptake | Uptake and Acceptance factors | Not reported | Not reported | Yes | 1 |
| 74 | Home Care | Mixed Across 3 LHINs (NE, TC, CW) servicing 6,334 patients | -- | Beyond Pilot | -- | -- | -- | Combination (COPD, HF) | -- | Program features | Ease of use | Program | Adherence | Implementation factors | Not reported | Not reported | Yes | 1 |
| 60 | -- | -- | -- | -- | GPs, staff nurses, district nurses, social care staff, health care professionals | -- | -- | Combination  (CHF, COPD) | -- | Program features | Ease of use | Provider | Acceptance | Acceptance | Staff Acceptance | Not reported | Unclear | 1 |
| 35 | -- | (Mixed) Multiple regions | -- | -- | -- | -- | Elderly | Combination (Diabetes, Heart disease) |  | Program features | Ease of use | User | Positive attitude | Views | Program Satisfaction | Not reported | Yes | 1 |
| 99 | -- | -- | -- | -- | Nurse | Speciality trained nurse from TMC | Recently discharged | **COPD** | **--** | Program features | Ease of use | User | Positive attitude, Confidence | Experiences, Program impact | Not reported | Not reported | Yes | *1* |

# CMO 2

| Study ID | Care Setting | Geography | Intervention | Implementation Stage | Provider -Role | Provider-Experience | Patient-Social | Chronic Condition | Patient-Health | Action Main | Action Sub | Response Main | Response Sub | Primary Outcome | Anticipated Outcome | Unanticipated Outcome | Outcome Success | Confidence in CMO |
| --- | --- | --- | --- | --- | --- | --- | --- | --- | --- | --- | --- | --- | --- | --- | --- | --- | --- | --- |
| 38 | -- | Multiple regions | Varying models used | -- | Community respiratory physiotherapists, community nurses, practice admin staff in consultation with GPs | -- | -- | COPD | Not Reported | User | Relationship building | Patient | Satisfaction | Relationship building | Impact on telemonitoring | Not reported | Unclear | 1 |
| 38 | -- | Multiple regions | Varying models used | -- | Community respiratory physiotherapists, community nurses, practice admin staff in consultation with GPs | -- | -- | COPD | Not Reported | User | Relationship building | Care | Improved care | Relationship building | Impact on telemonitoring | Not reported | Unclear | 1 |
| 55 | -- | Dispersed Native communities and/or villages, geographically dispersed | Varying types of RM technologies | -- | Healthcare providers (including non physician) | Have history of using telemedicine | Indigenous people | Diabetes | Not Reported | User | Relationship building | User | Communication | Perspectives | Benefits of using intervention | Implementation factors | Yes | 1 |
| 62 | -- | -- | Technical support provided | -- | GP, nurses, other Unscheduled Care Service (weekend) | -- | -- | HF | Not Reported | User | Relationship building | Care | Improved care | Acceptance, Perceived usefulness | Program Acceptance, Perceived Usefulness | Not reported | Yes | 1 |
| 77 | (Community) Varied in size and organizational structure | -- | -- | -- | Community matron, specialist nursing teams | -- | Level of education varied, majority non-white, experience with cell phone | Combination  (CHF, COPD) | Not Reported | User | Relationship building | Program | Cost (Financial Security) | Adoption factors | Not reported | Not reported | Unclear | 1 |
| 219 | (Tertiary) Nurse led clinic | -- | -- | -- | -- | -- | -- | Combination  (Diabetes, HF) | Not Reported | User | Relationship building | User | Acceptance | Feasibility, Perceived usefulness | Not reported | New normal practices/routine | Yes | 1 |
| 371 | (Community/ home) Community based hospital | -- | -- | Part of an RCT | -- | Different levels of interaction with intervention | -- | COPD | Not Reported | User | Relationship building | User | Perceived usefulness | Implementation factors | Not reported | Not reported | Unclear | 1 |

# CMO3

| Study ID | Care Setting | Geography | Intervention | Implementation Stage | Provider -Role | Provider-Experience | Patient-Social | Chronic Condition | Patient-Health | Action Main | Action Sub | Response Main | Response Sub | Primary Outcome | Anticipated Outcome | Unanticipated Outcome | Outcome Success | Confidence in CMO |
| --- | --- | --- | --- | --- | --- | --- | --- | --- | --- | --- | --- | --- | --- | --- | --- | --- | --- | --- |
| 21 | (Primary) Family health network | (Mixed) Semi-rural network | Provide broad range of care | -- | Family MDs, nurses | Providing care to ~10,000 patients | Social: Mostly retired | Combination (Diabetes, Cardiovascular disease, COPD) | Most frail or at risk patients in practice for functional decline or physical deterioration | Patient | Program experience | Patient | Sense of security | Feasibility, Efficacy | Program Acceptance | Not reported | Yes | 1 |
| 28 | -- | Geography: Urban area | -- | Expanding existsing telehealth activities | -- | -- | Social: Mostly white and women, | HF | Discharged from HF clinic | Patient | Program experience | Patient | Self-management | Experiences | Enhanced Clinical Outcomes, Perceptions | Not reported | Yes | 1 |
| 34 | Primary and home care | (Mixed) Across 3 LHINs (NE, TC, CW) servicing 6,334 patients | -- | -- | Diabetic nurses | -- | Social: High, medium levels of education,Mostly (93%) Dutch origin | Diabetes | Treatment satisfaction already high | Patient | Program experience | Patient | Communication; Positive experience | Implementation factors | Patient short and long term use of intervention | High preference for features of program (i.e. access to medication and treartment plan data) | Yes | 1 |
| 37 | (Tertiary) Major acute general hospital | -- | -- | -- | -- | Provided SM care | Social: Elderly patients | COPD |  | Patient | Program experience | Patient | Satisfaction | User satisfaction, Program effects | Program Satisfaction | Not reported | Yes | 1 |
| 41 | (Tertiary) HF clinic in hospital | Urban area | Patient education and access to care provider as needed | -- | -- | -- | -- | HF | Patients included those with implantable cardioverter defibrillator | Patient | Program experience | Patient | Self-management | Implementation factors, Program effects | Perceptions, Views | Not reported | Yes | 1 |
| 41 | (Tertiary) HF clinic in hospital | Urban area | Patient education and access to care provider as needed | -- | -- | -- | -- | HF | Patients included those with implantable cardioverter defibrillator | Patient | Program experience | Patient | Self-awareness | Implementation factors, Program effects | Perceptions, Views | Not reported | Yes | 1 |
| 41 | (Tertiary) HF clinic in hospital | Urban area | Patient education and access to care provider as needed | -- | -- | -- | -- | HF | Patients included those with implantable cardioverter defibrillator | Patient | Program experience | Patient | Reassurance | Implementation factors, Program effects | Perceptions, Views | Not reported | Yes | 1 |
| 94 | -- | Regional coverage across area with 1.4 M people | Lease options for technology, support and maintenance or standard devices | -- | Doctors, matron, renal nurses, business manager and technical staff. | -- | -- | CKD | -- | Patient | Program experience | Patient | Confidence | Perceived benefits | Not reported | Impact on team | Yes | 1 |
| 357 | -- | -- | -- | -- | -- | -- | -- | Diabetes | -- | Patient | Program experience | Patient | Satisfaction | Patient satisfaction, Experiences | Not reported | Not reported | Yes | 1 |

# CMO4

| Study ID | Care Setting | Geography | Intervention | Implementation Stage | Provider -Role | Provider-Experience | Patient-Social | Chronic Condition | Patient-Health | Action Main | Action Sub | Response Main | Response Sub | Primary Outcome | Anticipated Outcome | Unanticipated Outcome | Outcome Success | Confidence in CMO |
| --- | --- | --- | --- | --- | --- | --- | --- | --- | --- | --- | --- | --- | --- | --- | --- | --- | --- | --- |
| 27 | (Primary) practise site | (Mixed) Multiple regions | -- | Pilot phase | NP, RN and nutritionist | Team based approach, RNs were certified in SM diabetes care | White background | Diabetes | Mostly diabetic for 5 years or more | Program use | Program use | Patient | Self-management | Implementation factors, Satisfaction, Frequency of use, Clinical outcomes | Program Satisfaction | Not reported | Yes | 1 |
| 27 | (Primary) practise site | (Mixed) Multiple regions | -- | Pilot phase | NP, RN and nutritionist | Team based approach, RNs were certified in SM diabetes care | White background | Diabetes | Mostly diabetic for 5 years or more | Program use | Program use | User | Communication | Implementation factors, Satisfaction, Frequency of use, Clinical outcomes | Program Satisfaction | Not reported | Yes | 1 |
| 27 | (Primary) practise site | (Mixed) Multiple regions | -- | Pilot phase | NP, RN and nutritionist | Team based approach, RNs were certified in SM diabetes care | White background | Diabetes | Mostly diabetic for 5 years or more | Program use | Program use | User | Perceived usefulness | Implementation factors, Satisfaction, Frequency of use, Clinical outcomes | Program Satisfaction | Not reported | Yes | 1 |
| 34 | Primary and home care | (Mixed) Across 3 LHINs (NE, TC, CW) servicing 6,334 patients | Instructions on frequency of use not provided, training to use equipment and access to technical support provided; limited initial development costs and low running costs | -- | Diabetic nurses | -- | High, medium levels of education,Mostly (93%) Dutch origin | Diabetes | Treatment satisfaction already high | Program use | Program use | Patient | Self-management | Implementation factors | Patient short and long term use of intervention | High preference for features of program (i.e. access to medication and treartment plan data) | Yes | 1 |
| 51 | (Tertiary) Multiple regional hospitals | Urban area | -- | -- | -- | Certified diabetes case managers | -- | Diabetes | -- | Program use | Program use | Care | Improved care | Experiences, Acceptance, Perceived effectiveness | Acceptance, Perceived effectiveness | Not reported | Yes | 1 |
| 71 | -- | -- | Varied approach (self-management vs. remote monitoring) | -- | -- | Interactions between provider and patients varied | -- | COPD | -- | Program use | Program use | Patient | Self-empowerment | Perspectives, Experiences | Not reported | Not reported | Unclear | 1 |
| 94 | -- | Regional coverage across area with 1.4 M people | Lease option Sup for technology, support and maintenance or standard devices | -- | Doctors, matron, renal nurses, business manager and technical staff. | -- | -- | CKD | -- | Program use | Program use | Provider | Time management | Perceived benefits | Not reported | Impact on team | Yes | 1 |
| 94 |  |  |  |  |  |  |  |  |  | Program use | Program use | Provider | Reassurance | Perceived benefits | Not reported | Impact on team | Yes | 1 |
| 94 |  |  |  |  |  |  |  |  |  | Program use | Program use | Provider | Job satisfaction | Perceived benefits | Not reported | Impact on team | Yes | 1 |
| 100 | Primary care | -- | Varying equipment and processes | Routine | -- | -- | -- | Diabetes | High risk patients | Program use | Program use | Care | Improved care | Experiences | Not reported | Unexpected benefits | Yes | 1 |
| 222 | (Tertiary) Medical Centre serving 33 counties | -- | -- | -- | -- | -- | Mixed ethnic background | HF | -- | Program use | Program use | Patient | Self-management | Patient adherence, Self-reported use | Not reported | Patient self concept may be better predictor of adherence | Yes | 1 |
| 312 | -- | -- | -- | -- | Care team | -- | -- | HF | Stable AF condition | Program use | Program use | Patient | Self-management | Usability, Acceptance | Not reported | Not reported | Yes | 1 |
| 349 | -- | -- | -- | -- | -- | -- | Various ethnic background, marital sttaus and insurance status | HF | -- | Program use | Program use | Patient | Self-management | Patient Experiences | Not reported | Not reported | Yes | 1 |
| 353 | Primary and home care | Urban | Training provided | Pilot phase | Home care agency | Training provided | Employed and retired | HF | -- | Program use | Program use | Patient | Self-management | Feasability | Not reported | Not reported | No | 1 |
| 357 | -- | -- | -- | -- | -- | -- |  | Diabetes | -- | Program use | Program use | Provider | Time management | Patient satisfaction, Experiences | Not reported | Not reported | Yes | 1 |
| 380 | -- | -- | -- | -- | -- | -- | -- | Combination  (CKD, COPD, HF, Diabetes) | -- | Program use | Program use | Care | Improved care | Patient beliefs, Attitudes, Expectations and Experiences | Not reported | Not reported | Yes | 1 |
| 380 | -- | -- | -- | -- | -- | -- | -- | Combination  (CKD, COPD, HF, Diabetes) | -- | Program use | Program use | Patient | Self-management | Patient beliefs, Attitudes, Expectations and Experiences | Not reported | Not reported | Yes | 1 |
| 380 | -- | -- | -- | -- | -- | -- | -- | Combination  (CKD, COPD, HF, Diabetes) | -- | Program use | Program use | Patient | Security | Patient beliefs, Attitudes, Expectations and Experiences | Not reported | Not reported | Yes | 1 |
| 380 | -- | -- | -- | -- | -- | -- | -- | Combination  (CKD, COPD, HF, Diabetes) | -- | Program use | Program use | Patient | Security | Patient beliefs, Attitudes, Expectations and Experiences | Not reported | Not reported | Yes | 1 |

# CMO5

| Study ID | Care Setting | Geography | Intervention | Implementation Stage | Provider -Role | Provider-Experience | Patient-Social | Chronic Condition | Patient-Health | Action Main | Action Sub | Response Main | Response Sub | Primary Outcome | Anticipated Outcome | Unanticipated Outcome | Outcome Success | Confidence in CMO |
| --- | --- | --- | --- | --- | --- | --- | --- | --- | --- | --- | --- | --- | --- | --- | --- | --- | --- | --- |
| 21 | (Primary) Family health network | (Mixed) Semi-rural network | Range of care, technical support provided | Provide broad range of care | Family MDs, nurses | Providing care to ~10,000 patients | Mostly retired | Combination (Diabetes, Cardiovascular disease, COPD) | Most frail or at risk patients in practice for functional decline or physical deterioration | Motivation | Reduced workload | Provider | Perceived usefulness | Feasability, Efficacy | Program Acceptance | Not reported | Yes | 1 |
| 26 | -- | (Mixed) Urban and Rural areas | -- | -- | -- | Different ethnic backgrounds, 19 years of experience | -- | Diabetes | -- | Motivation | Comfort, convenience | Patient | Ease of use | Experiences | Provider Satisfaction, Perceived benefits of using intervention | Not reported | Yes | 1 |
| 49 | (Primary) Small (10 GPs) and large practice (10+ GP) | Urban area | -- | -- | Included allied health services, including mental health and CDM nurses | -- | -- | Diabetes | Poorly controlled diabetes | Motivation | Relationship building | Provider | Perceived usefulness | Experiences, Perceptions | Implementation factors | Not reported | Yes | 1 |
| 54 | (Primary) Multiple care sites | -- | -- | -- | -- | -- | 86.3-86.9% White British ethnicity | Combination  (COPD, Diabetes, HF) | -- | Motivation | Improved health | Program | Cost (Effectiveness) | Cost, Cost effectiveness | Not reported | Not reported | No | 1 |
| 55 | -- | Dispersed Native communities and/or villages, eographically dispersed | Range of models | -- | Healthcare providers (including non physician) | Have history of using telemedicine | Indigenous populations | Diabetes | -- | Motivation | Reduced travel, healthcare costs | User | Acceptance | Perspectives | Benefits of using intervention | Implementation factors | Yes | 1 |
| 62 | -- | -- | Technical support provided | -- | GP, nurses, other Unscheduled Care Service (weekend) | -- | -- | HF | -- | Motivation | Support and reassurance | Patient | Satisfaction | Acceptance, Perceived usefulness | Program Acceptance, Perceived Usefulness | Not reported | Yes | 1 |
| 63 | -- | -- | -- | -- | -- | -- | -- | Combination  (COPD, HF) | -- | Motivation | Self-management | Program | Uptake | Uptake and Acceptance factors | Not reported | Not reported | Yes | 1 |
| 67 | -- | -- | -- | -- | -- | -- | -- | Diabetes | -- | Motivation | Ease of use;  Perceived usefulness | Patient | Satisfaction | User satisfaction, Compliance, Clinical outcomes | User Assessments | Not reported | Yes | 1 |
| 74 | Home care | Mixed Across 3 LHINs (NE, TC, CW) servicing 6,334 patients | -- | Beyond Pilot Phase | -- | -- | -- | Combination (COPD, HF) | -- | Motivation | Patient-level | Program | Use | Implementation factors | Not reported | Not reported | Yes | 1 |
| 79 | -- | -- | -- | -- | -- | -- | -- | Combination  (COPD, Diabetes, HF) | -- | Motivation | Empathy, Acceptance | Program | Uptake | Core mechanisms | Program uptake | Not reported | Unclear | 1 |
| 100 | Primary care practices and home care agencies | -- | Range of equipment and services | Beyond Pilot Phase | -- | -- | -- | Diabetes | High risk patients | Motivation | Relationship building, Trust | User | Experience | Experiences | Not reported | Unexpected benefits | Yes | 1 |
| 221 |  | -- | -- | -- | -- | -- |  | Combination  (COPD, Diabetes, HF) | -- | Motivation | Empowerment, confidence Program: easy to use | Program | Adoption | Implementation factors, Experiences | Not reported | Patients used data to manage day to day activity | Unclear | 1 |
| 222 | (Tertiary) Medical Centre serving 33 counties | -- | -- | -- | -- | -- | Mixed ethnic background | HF | -- | Motivation | Values and self-identity | Program | Adoption | Patient adherence, Self-reported use | Not reported | Patient self concept may be better predictor of adherence | Yes | 1 |
| 222 |  |  |  |  |  |  |  |  |  | Motivation | Personal motivation | Program | Cost (Staffing) | Patient adherence, Self-reported use | Not reported | Patient self concept may be better predictor of adherence | Yes | 1 |
| 386 | -- | -- | -- | -- | -- | -- | -- | Combination  (HF, Cadiovascular disease) | -- | Motivation | Willingess and desire to learn | Care | Access to care | Engagement factors | Not reported | Not reported | Unclear | 1 |
| 394 | -- | -- | -- | -- | -- | -- | -- | Combination (Cardiovascular disease, COPD, Diabetes) | -- | Motivation | Self-management | Program | Acceptance | Implementaton factors | Not reported | Not reported | Unclear | 1 |
