# Supplementary material for: A realist review of factors critical for the implementation of eHealth in chronic disease management
Source: BMC Health Serv Res. 2025 Apr 2;25:496. doi: 10.1186/s12913-025-12361-0 (PMC11966836; doi:10.1186/s12913-025-12361-0)
Supplement: Supplementary file 6 — Supplementary Material 6. [file 12913_2025_12361_MOESM6_ESM.docx]

## Appendix 6. Hindering CMOs

# CM06

| Study ID | Care Setting | Geography | Intervention | Implementation Stage | Provider -Role | Provider-Experience | Patient-Social | Chronic Condition | Patient-Health | Action Main | Action Sub | Response Main | Response Sub | Primary Outcome | Anticipated Outcome | Unanticipated Outcome | Outcome Success | Confidence in CMO |
| --- | --- | --- | --- | --- | --- | --- | --- | --- | --- | --- | --- | --- | --- | --- | --- | --- | --- | --- |
| 60 | -- | -- | -- | -- | GPs, staff nurses, district nurses, social care staff, health care professionals | -- | -- | Combination  (CHF, COPD) | -- | User | Perceived usefulness | Provider | Poor staff acceptance | Acceptance | Staff Acceptance | Not reported | Unclear | 1 |
| 60 | -- | -- | -- | -- | GPs, staff nurses, district nurses, social care staff, health care professionals | -- | -- | Combination  (CHF, COPD) | -- | User | Low literacy (Technical) | Provider | Poor staff acceptance | Acceptance | Staff Acceptance | Not reported | Unclear | 1 |
| 63 | -- | -- | -- | -- |  | -- |  | Combination  (COPD, HF) | -- | User | Perceived usefulness | Implementation | Uptake | Uptake and Acceptance factors | Not reported | Not reported | Yes | 1 |
| 93 | -- | (Mixed) Urban and Rural areas | -- | -- | Provider | -- | -- | Diabetes | -- | User | Low literacy (Technical) | Implementation | Implementation barrier | Adoption barriers | Not reported | Not reported | Unclear | 1 |
| 93 | -- | (Mixed) Urban and Rural areas | -- | -- | Provider | -- | -- | Diabetes | -- | User | Low literacy (Health) | Implementation | Implementation barrier | Adoption barriers | Not reported | Not reported | Unclear | 1 |
| 222 | Tertiary Care (Medical Centre serving 33 counties) | -- | -- | -- | -- | -- | Mixed ethnic background | HF | -- | User | Perceived usefulness | User | Perceived usability | Patient adherence, Self-reported use | Not reported | Patient self concept may be better predictor of adherence | Yes | 1 |
| 380 | -- | -- | -- | -- | -- | -- | -- | Combination  (CKD, COPD, HF, Diabetes) | -- | User | Perceived usefulness | Implementation | Uptake | Patient beliefs, Attitudes, Expectations and Experiences | Not reported | Not reported | Yes | 1 |
| 380 | -- | -- | -- | -- | -- | -- | -- | Combination  (CKD, COPD, HF, Diabetes) | -- | User | Perceived usefulness | Implementation | Uptake | Patient beliefs, Attitudes, Expectations and Experiences | Not reported | Not reported | Yes | 1 |
| 19 | Community / home care | -- | Range of devices, re-designed incorporating multiple viewpoints | -- | -- | Limited participation due to high workload demands | -- | COPD | Discharged patients | Technology | Technical problems | Organizational | Time loss | Implementation factors | Change management, Usability, Best practices | Not reported | Unclear | 1 |
| 23 | -- | Underserved | -- | -- | -- | -- | 95% white | Diabetes | Living with Diabetes | Technology | Technical problems | Patient | Negative experience | Experiences | Program Satisfaction/Acceptance | Not reported | Yes | 1 |
| 26 | -- |  | -- | -- | -- | Different ethnic backgrounds, 19 years of experience | -- | Diabetes | -- | Technology | Technical problems | Provider, Care | Negative experience, care disruption | Experiences | Provider Satisfaction, Perceived benefits of using intervention | Not reported | Yes | 1 |
| 38 | -- | -- | Varying models used | -- | Community respiratory physiotherapists, community nurses, practice admin staff in consultation with GPs | -- | -- | COPD | -- | Technology | Technical problems | Provider | Negative experience | Views | Impact on telemonitoring | Not reported | Unclear | 1 |
| 43 | -- | Underserved | -- | -- | RN managers, Physiotherapy managers, Physiotherapists, Non-clinical managers, Community nurse managers | -- | -- | COPD | Moderate, severe COPD | Technology | Technical problems | User | Negative experience | Acceptability, Feasability | Not reported | Not reported | Yes | 1 |
| 60 | -- | -- | -- | -- | GPs, staff nurses, district nurses, social care staff, health care professionals | -- | -- | Combination  (CHF, COPD) |  | Technology | Technical problems | Provider | Poor staff acceptance | Acceptance | Staff Acceptance | Not reported | Unclear | 1 |
| 63 | -- | -- | -- | -- | -- | -- | -- | Combination  (COPD, HF) | -- | Technology | Technical problems | Implementation | Uptake | Uptake and Acceptance factors | Not reported | Not reported | Yes | 1 |
| 63 | -- | -- | -- | -- | -- | -- | -- | Combination  (COPD, HF) | -- | Technology | Technical problems | Implementation | Uptake | Uptake and Acceptance factors | Not reported | Not reported | Yes | 1 |
| 74 | Community/home care | Mixed Across 3 LHINs (NE, TC, CW) servicing 6,334 patients | -- | Beyond Pilot Phase | -- | -- | -- | Combination (COPD, HF) | -- | Technology | Technical problems | Implementation | Implementation | Implementation factors | Not reported | Not reported | Yes | 1 |
| 117 | -- | -- | Several components | -- | -- | -- | -- | COPD | Stable and non-stable | Technology | Technical problems | User | Acceptance | Acceptance, Adherence, Acceptance factors | Not reported | Not reported | Yes | 1 |

# CMO8

| Study ID | Care Setting | Geography | Intervention | Implementation Stage | Provider -Role | Provider-Experience | Patient-Social | Chronic Condition | Patient-Health | Action Main | Action Sub | Response Main | Response Sub | Primary Outcome | Anticipated Outcome | Unanticipated Outcome | Outcome Success | Confidence in CMO |
| --- | --- | --- | --- | --- | --- | --- | --- | --- | --- | --- | --- | --- | --- | --- | --- | --- | --- | --- |
| 16 | Primary Care | Underserved | -- | -- | Primary care providers | Minimum 1 year experience with intervention | -- | Diabetes | -- | Organizational | Mundane components, Conflicting information | Provider | Increased workload | Acceptance, Perceived impact | Program Acceptance (by primary care providers) | Not reported | Yes | 1 |
| 23 | -- | Underserved, rural areas | -- | -- | -- | -- | 95% white | Diabetes | Living with Diabetes 9-11 years | Organizational | Changes | User | Reduced learning | Experiences | Program Satisfaction/Acceptance | Not reported | Yes | 1 |
| 38 | -- | -- | Varying models used | -- | Community respiratory physiotherapists, community nurses, practice admin staff in consultation with GPs | -- | -- | COPD | -- | Organizational | Conflicting information | Organizational | Operational problems | Views | Impact on telemonitoring | Not reported | Unclear | 1 |
| 38 | -- | -- | Varying models used | -- | Community respiratory physiotherapists, community nurses, practice admin staff in consultation with GPs | -- | -- | COPD | -- | Organizational | Conflicting information | Provider | Negative experience | Views | Impact on telemonitoring | Not reported | Unclear | 1 |
| 41 | (Tertiary) HF clinic in hospital | Urban area | Patient education and access to care provider as needed | -- | -- | -- | -- | Heart Failure | Patients included those with implantable cardioverter defibrillator | Organizational | Operational problems | Implementation | Implementation barrier | Implementation factors, Program effects | Perceptions, Views | Not reported | Yes | 1 |
| 49 | (Primary) Small (10 GPs) and large practice (10+ GP) | Urban area | -- | -- | Included allied health services, including mental health and CDM nurses | -- | -- | Diabetes | Poorly controlled diabetes | Organizational | Operational problems | Implementation | Implementation barrier | Experiences, Perceptions | Implementation factors | Not reported | Yes | 1 |
| 60 | -- | -- | -- | -- | GPs, staff nurses, district nurses, social care staff, health care professionals | -- | -- | Combination  (CHF, COPD) | -- | Organizational | Disruption | Provider | Poor staff acceptance | Acceptance | Staff Acceptance | Not reported | Unclear | 1 |
| 60 | -- | -- | -- | -- | GPs, staff nurses, district nurses, social care staff, health care professionals | -- | -- | Combination  (CHF, COPD) | -- | Organizational | Low efficiency | Provider | Poor staff acceptance | Acceptance | Staff Acceptance | Not reported | Unclear | 1 |
| 60 | -- | -- | -- | -- | GPs, staff nurses, district nurses, social care staff, health care professionals | -- | -- | Combination  (CHF, COPD) | -- | Organizational | Poor change management | Provider | Poor staff acceptance | Uptake and Acceptance factors | Not reported | Not reported | Yes | 1 |
| 63 | -- | -- | -- | -- | -- | -- | -- | Combination  (COPD, HF) | -- | Organizational | Mundane components | Implementation | Uptake | Uptake and Acceptance factors | Not reported | Not reported | Yes | 1 |
| 65 | -- | -- | -- | -- | -- | -- | -- | Diabetes | -- | Organizational | Operational problems | Provider | Increased workload | Implementation factors | Not reported | Not reported | Unclear | 1 |
| 68 | Home (community) care | Urban area | -- | Beyond Pilot Phase | HF nurses, Community matrons and community support workers | -- | -- | HF | -- | Organizational | Disruption | User | Negative experience | Experiences | Disruption Experience | Not reported | Unclear | 1 |
| 93 | -- | Mixed: Urban, Semi-rural, rural | -- | -- | Provider | -- | -- | Diabetes | -- | Organizational | Lack of workflow integration | Implementation | Implementation barrier | Adoption barriers | Not reported | Not reported | Unclear | 1 |

# CMO9

| Study ID | Care Setting | Geography | Intervention | Implementation Stage | Provider -Role | Provider-Experience | Patient-Social | Chronic Condition | Patient-Health | Action Main | Action Sub | Response Main | Response Sub | Primary Outcome | Anticipated Outcome | Unanticipated Outcome | Outcome Success | Confidence in CMO |
| --- | --- | --- | --- | --- | --- | --- | --- | --- | --- | --- | --- | --- | --- | --- | --- | --- | --- | --- |
| 55 | -- | Native communities/villages, geographically disperesed | Range of models | -- | Healthcare providers (including non physician) | Have history of using telemedicine | Indigenous populations | Diabetes | -- | SDOH | Distance | User | Perceived usability | Perspectives | Benefits of using intervention | Implementation factors | Yes | 1 |
| 63 | -- | -- | -- | -- | -- | -- | -- | Combination  (COPD, HF) | -- | SDOH | Distance | Implementation | Uptake | Uptake and Acceptance factors | Not reported | Not reported | Yes | 1 |
| 74 | -- | Mixed: Urban, Semi-rural, rural | -- | Beyond Pilot Phase | -- | -- | -- | Combination (COPD, HF) | -- | SDOH | Distance | Implementation | Implementation | Implementation factors | Not reported | Not reported | Yes | 1 |
| 380 | -- | -- | -- | -- | -- | -- | -- | Combination  (CKD, COPD, HF, Diabetes) | -- | SDOH | Distance | Implementation | Implementation barrier | Patient beliefs, Attitudes, Expectations and Experiences | Not reported | Not reported | Yes | 1 |
| 386 | -- | -- | -- | -- | -- | -- | -- | Combination  (HF, Cadiovascular disease) | -- | SDOH | Distance | Implementation | Implementation barrier | Engagement factors | Not reported | Not reported | Unclear | 1 |

# CMO10

| Study ID | Care Setting | Geography | Intervention | Implementation Stage | Provider -Role | Provider-Experience | Patient-Social | Chronic Condition | Patient-Health | Action Main | Action Sub | Response Main | Response Sub | Primary Outcome | Anticipated Outcome | Unanticipated Outcome | Outcome Success | Confidence in CMO |
| --- | --- | --- | --- | --- | --- | --- | --- | --- | --- | --- | --- | --- | --- | --- | --- | --- | --- | --- |
| 11 | -- | -- | -- | -- | -- | -- | -- | Diabetes | Includes pediatric population | Program use | Increased involvement (patient communication, need for information) | Provider | Increased workload | Implementation factors | Program Acceptance (by patients) | Cost return | Yes | 0 |
| 13 | Tertiary care | Multiple medical centres | -- | -- | Hospital nurse | -- | -- | HF | -- | Program use | Increased involvement (data entry, reminders) | User | Negative experience | Feasability | Feasability | Benefits of integrating | Yes | 1 |
| 41 | (Tertiary) HF clinic in hospital | Urban area | Patient education and access to care provider as needed | -- | -- | -- | -- | HF | Patients included those with implantable cardioverter defibrillator | Program use | Increased involvement (clinical workload, workflow changes) | Implementation | Implementation barrier | Implementation factors, Program effects | Perceptions, Views | Not reported | Yes | 1 |
| 62 | -- | -- | Technical support provided | -- | GP, nurses, other Unscheduled Care Service (weekend) | -- | -- | HF | -- | Program use | Increased involvement (additional workload) | Provider | Negative experience | Acceptance, Perceived usefulness | Program Acceptance, Perceived Usefulness | Not reported | Yes | 1 |
| 118 | (Tertiary) Hospital | -- | -- | Pilot phase | Nurses, family MDs and information technology | Varied from 3months to 4 years | -- | Combination  (Diabetes, Hypertension) | Geriatric | Program use | Increased involvement | Organizational | Care disruption | Cultural factors | Cultural factors | Not reported | Unclear | 1 |
| 315 | -- | -- | -- | -- | -- | -- | -- | Diabetes | -- | Program use | Increased involvement (workload), limited time | Implementation | Implementation barrier | Implementation factors | Not reported | Not reported | Unclear | 1 |
| 371 | (Community/home care) hospital | -- | -- | Part of an RCT | -- | Different levels of interaction with intervention | -- | COPD | -- | Program use | Technical involvement | User | Perceived usability | Implementation factors | Not reported | Not reported | Unclear | 1 |
| 371 | (Community/home care) hospital | -- | -- | Part of an RCT | -- | Different levels of interaction with intervention | -- | COPD | -- | Program use | Increased involvement | Organizational | Increased workload | Implementation factors | Not reported | Not reported | Unclear | 1 |
