# Supplementary material for: A realist review of factors critical for the implementation of eHealth in chronic disease management
Source: BMC Health Serv Res. 2025 Apr 2;25:496. doi: 10.1186/s12913-025-12361-0 (PMC11966836; doi:10.1186/s12913-025-12361-0)
Supplement: Supplementary file 7 — Supplementary Material 7. [file 12913_2025_12361_MOESM7_ESM.pptx]

## Slide 1
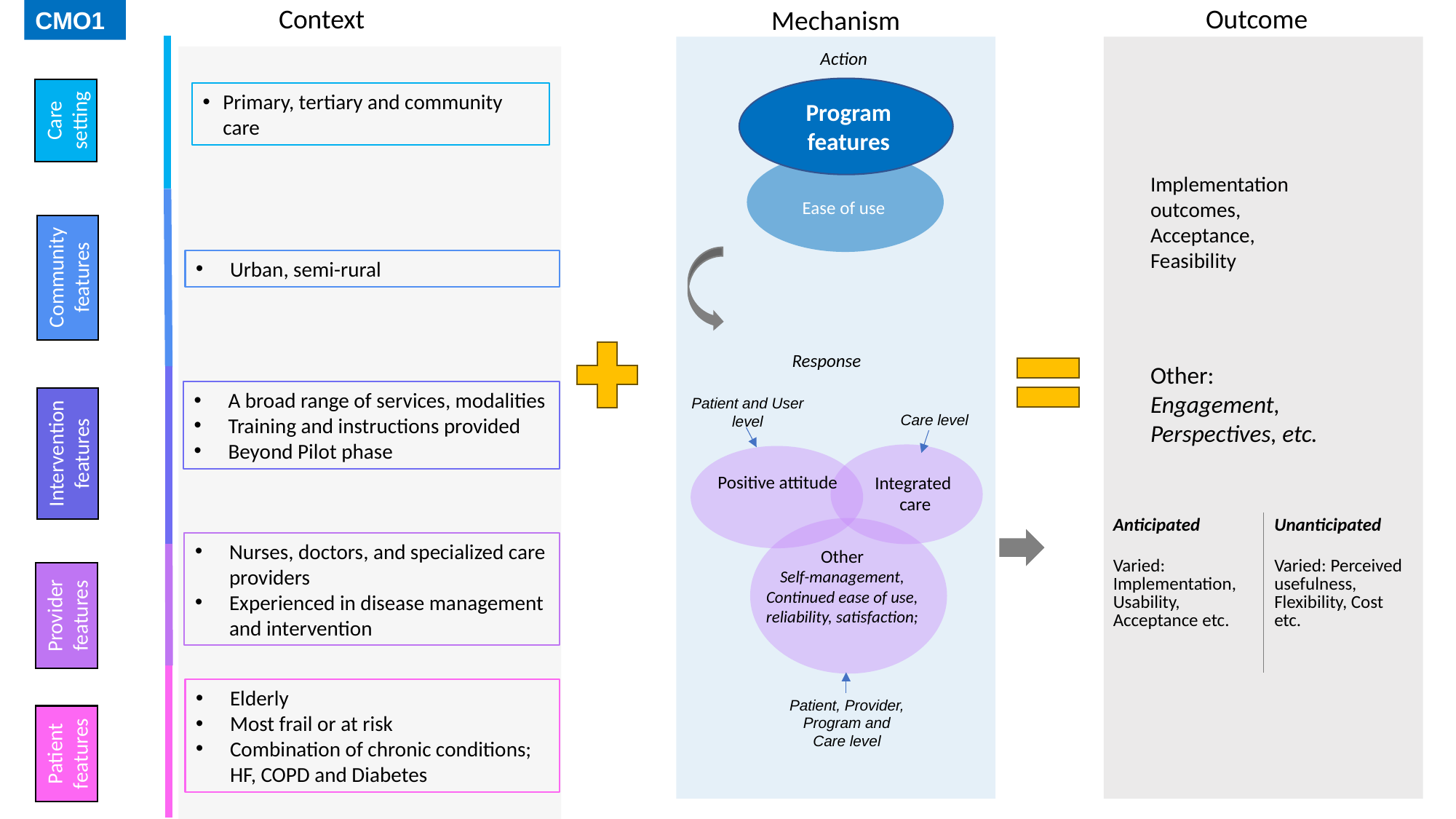

CMO1
Context
Outcome
Mechanism
Action
Primary, tertiary and community care
Care setting
Program features
Implementation outcomes, Acceptance, Feasibility
Other:
Engagement, Perspectives, etc.
Ease of use
Community features
Urban, semi-rural
Response
A broad range of services, modalities
Training and instructions provided
Beyond Pilot phase
Patient and User level
Care level
Intervention features
Positive attitude
Integrated
 care
| Anticipated | Unanticipated |
| --- | --- |
| Varied: Implementation, Usability, Acceptance etc. | Varied: Perceived usefulness, Flexibility, Cost etc. |
Nurses, doctors, and specialized care providers
Experienced in disease management and intervention
Other
Self-management, Continued ease of use, reliability, satisfaction;
Provider features
Elderly
Most frail or at risk
Combination of chronic conditions; HF, COPD and Diabetes
Patient, Provider, Program and Care level
Patient features

## Slide 2
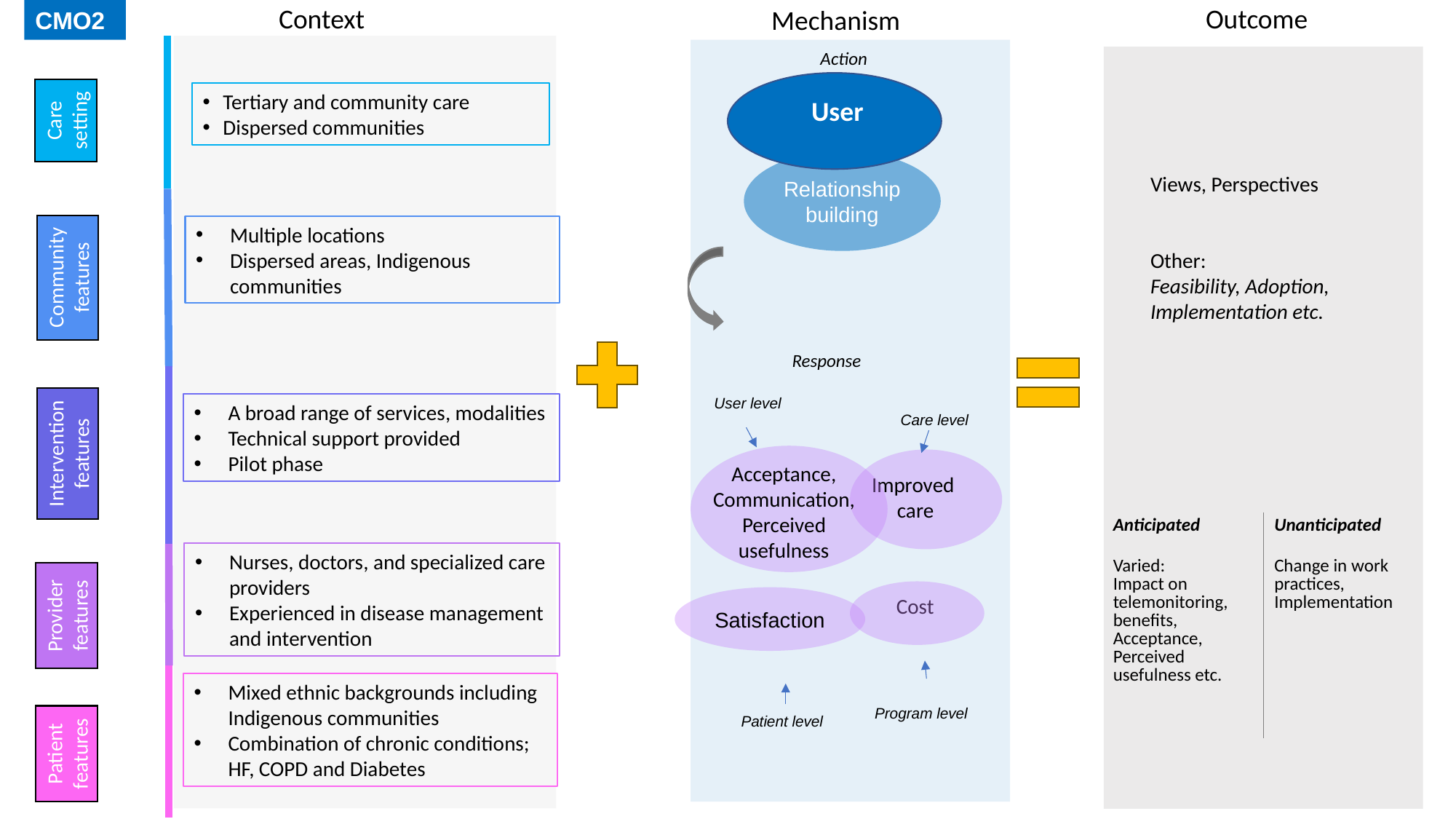

CMO2
Context
Outcome
Mechanism
Action
Tertiary and community care
Dispersed communities
User
Care setting
Relationship building
Views, Perspectives
Other:
Feasibility, Adoption, Implementation etc.
Multiple locations
Dispersed areas, Indigenous communities
Community features
Response
User level
A broad range of services, modalities
Technical support provided
Pilot phase
Care level
Intervention features
Acceptance, Communication, Perceived usefulness
Improved
 care
| Anticipated | Unanticipated |
| --- | --- |
| Varied: Impact on telemonitoring, benefits, Acceptance, Perceived usefulness etc. | Change in work practices, Implementation |
Nurses, doctors, and specialized care providers
Experienced in disease management and intervention
Provider features
Satisfaction
Cost
Mixed ethnic backgrounds including Indigenous communities
Combination of chronic conditions; HF, COPD and Diabetes
Program level
Patient level
Patient features

## Slide 3
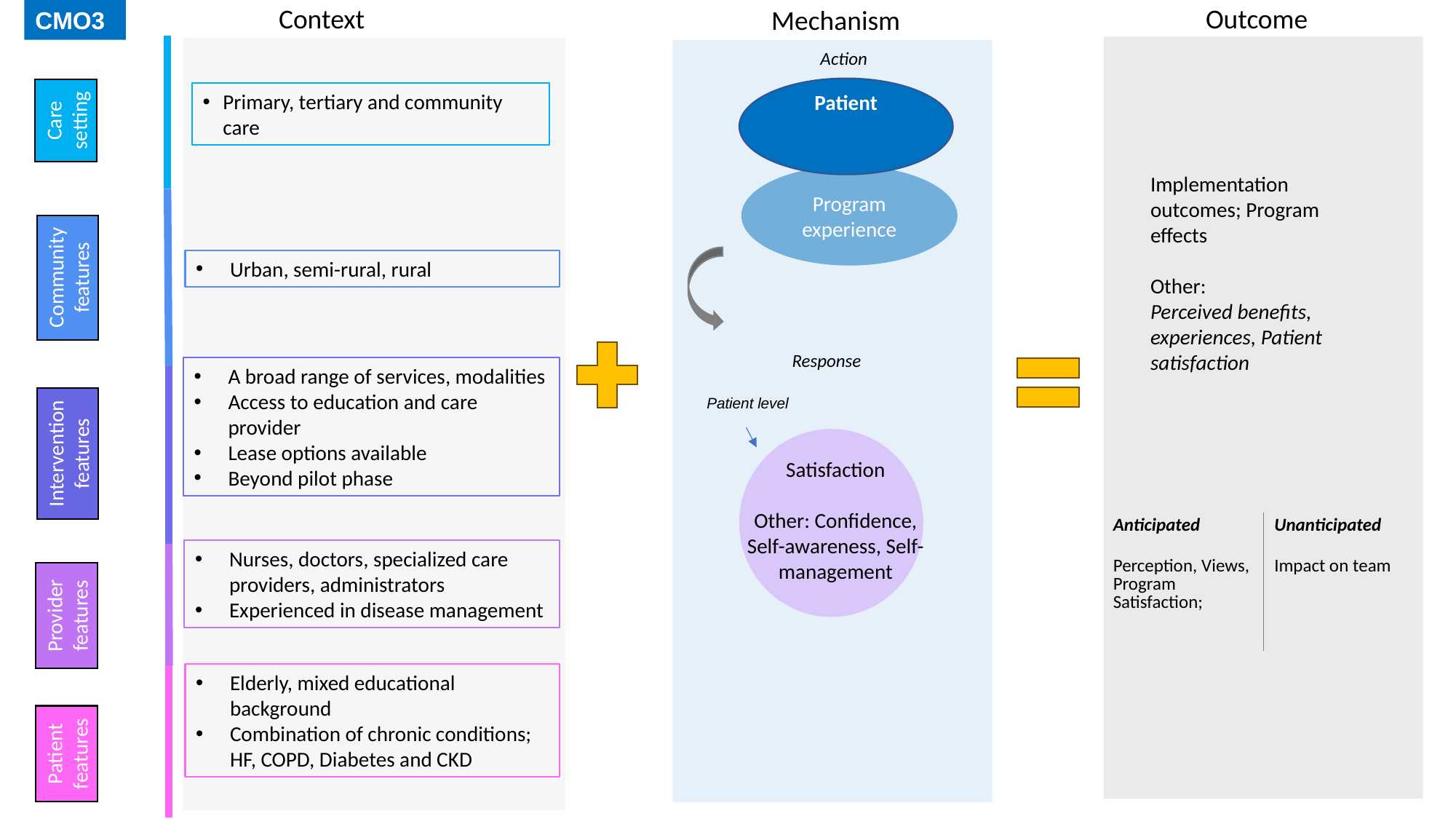

CMO3
Context
Outcome
Mechanism
Action
Primary, tertiary and community care
Patient
Care setting
Implementation outcomes; Program effects
Other:
Perceived benefits, experiences, Patient satisfaction
Program experience
Community features
Urban, semi-rural, rural
Response
A broad range of services, modalities
Access to education and care provider
Lease options available
Beyond pilot phase
Patient level
Intervention features
Satisfaction
Other: Confidence, Self-awareness, Self-management
| Anticipated | Unanticipated |
| --- | --- |
| Perception, Views, Program Satisfaction; | Impact on team |
Nurses, doctors, specialized care providers, administrators
Experienced in disease management
Provider features
Elderly, mixed educational background
Combination of chronic conditions; HF, COPD, Diabetes and CKD
Patient features

## Slide 4
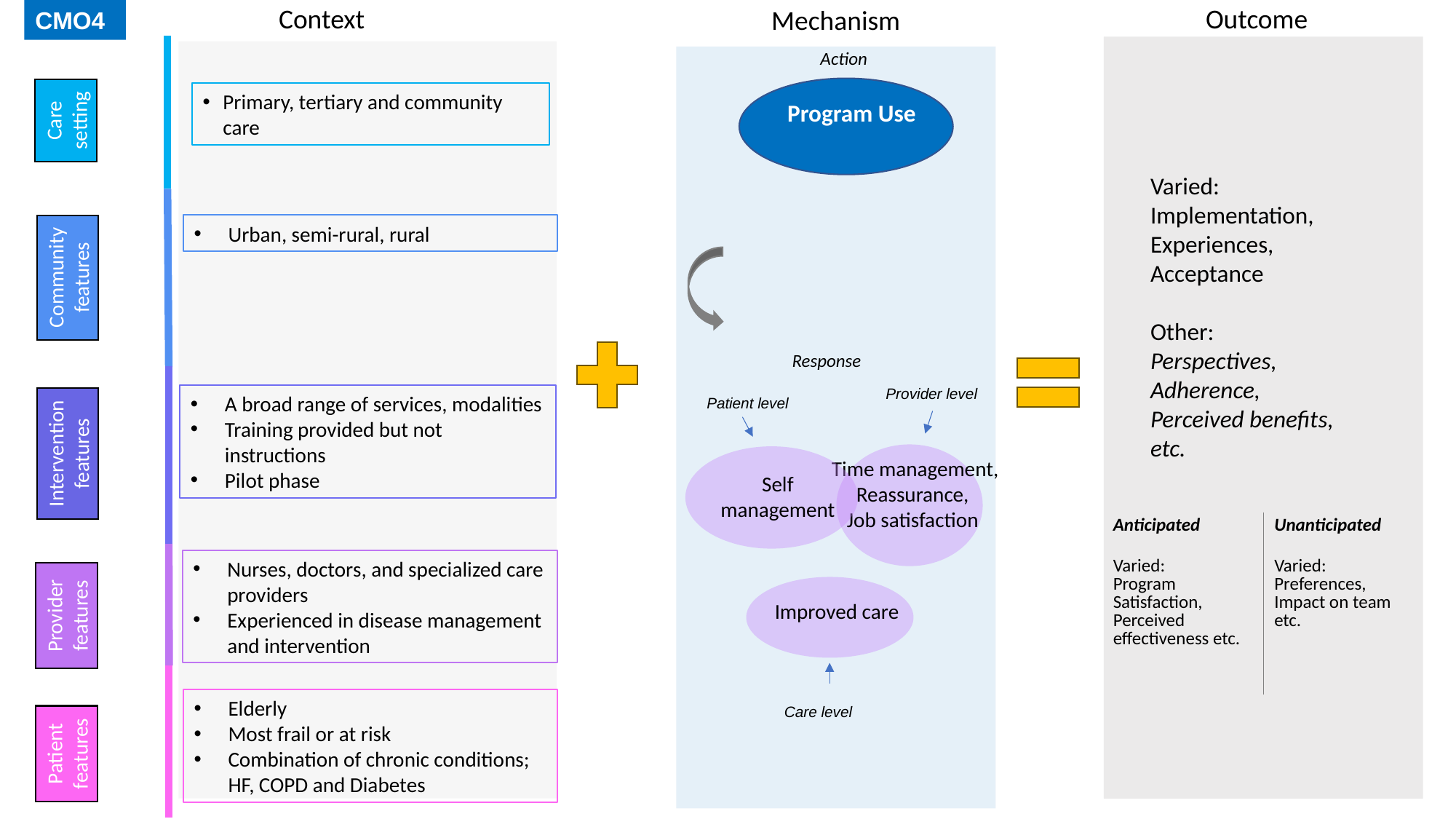

CMO4
Context
Outcome
Mechanism
Action
Primary, tertiary and community care
Care setting
Program Use
Varied: Implementation, Experiences, Acceptance
Other:
Perspectives, Adherence, Perceived benefits, etc.
Urban, semi-rural, rural
Community features
Response
Provider level
A broad range of services, modalities
Training provided but not instructions
Pilot phase
Patient level
Intervention features
Time management, Reassurance,
Job satisfaction
Self management
| Anticipated | Unanticipated |
| --- | --- |
| Varied: Program Satisfaction, Perceived effectiveness etc. | Varied: Preferences, Impact on team etc. |
Nurses, doctors, and specialized care providers
Experienced in disease management and intervention
Provider features
Improved care
Elderly
Most frail or at risk
Combination of chronic conditions; HF, COPD and Diabetes
Care level
Patient features

## Slide 5
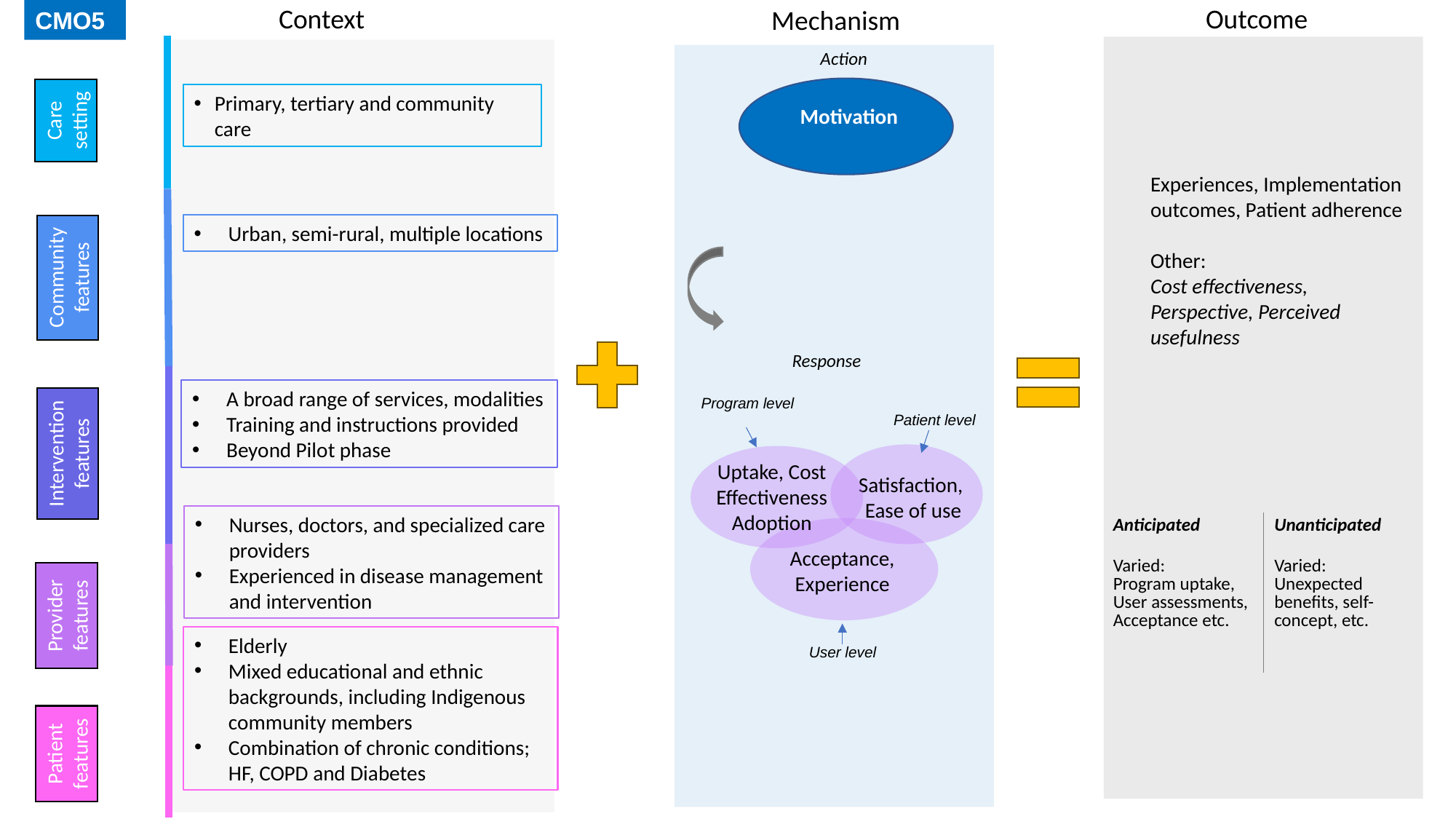

CMO5
Context
Outcome
Mechanism
Action
Primary, tertiary and community care
Care setting
Motivation
Experiences, Implementation outcomes, Patient adherence
Other:
Cost effectiveness, Perspective, Perceived usefulness
Urban, semi-rural, multiple locations
Community features
Response
A broad range of services, modalities
Training and instructions provided
Beyond Pilot phase
Program level
Patient level
Intervention features
Uptake, Cost Effectiveness Adoption
Satisfaction,
Ease of use
Nurses, doctors, and specialized care providers
Experienced in disease management and intervention
| Anticipated | Unanticipated |
| --- | --- |
| Varied: Program uptake, User assessments, Acceptance etc. | Varied: Unexpected benefits, self-concept, etc. |
Acceptance,
Experience
Provider features
Elderly
Mixed educational and ethnic backgrounds, including Indigenous community members
Combination of chronic conditions; HF, COPD and Diabetes
User level
Patient features

## Slide 6
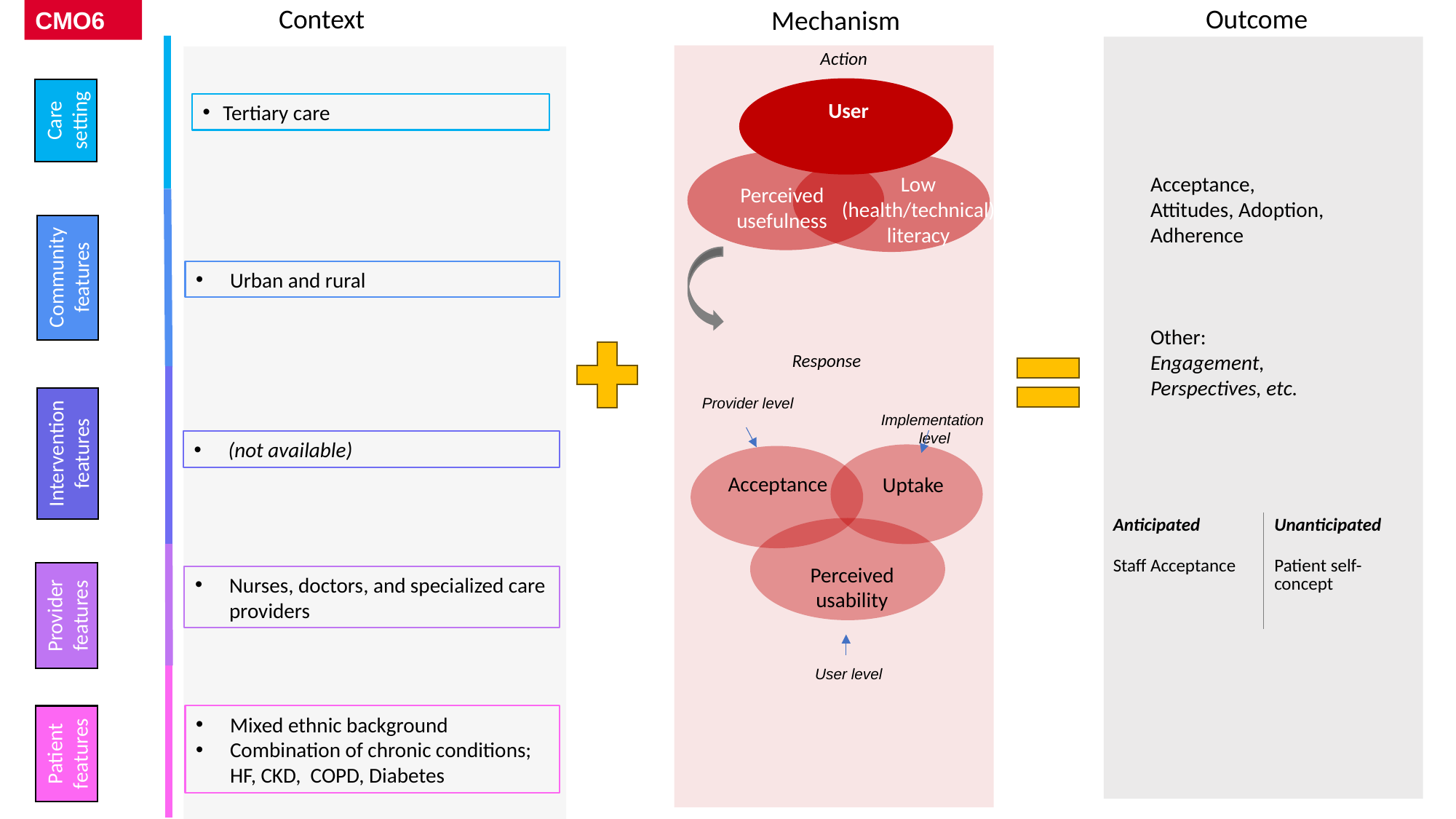

CMO6
Context
Outcome
Mechanism
Action
Care setting
User
Tertiary care
Low (health/technical) literacy
Acceptance, Attitudes, Adoption, Adherence
Other:
Engagement, Perspectives, etc.
Perceived usefulness
Community features
Urban and rural
Response
Provider level
Implementation level
Intervention features
(not available)
Acceptance
Uptake
| Anticipated | Unanticipated |
| --- | --- |
| Staff Acceptance | Patient self-concept |
Perceived usability
Nurses, doctors, and specialized care providers
Provider features
User level
Mixed ethnic background
Combination of chronic conditions; HF, CKD, COPD, Diabetes
Patient features

## Slide 7
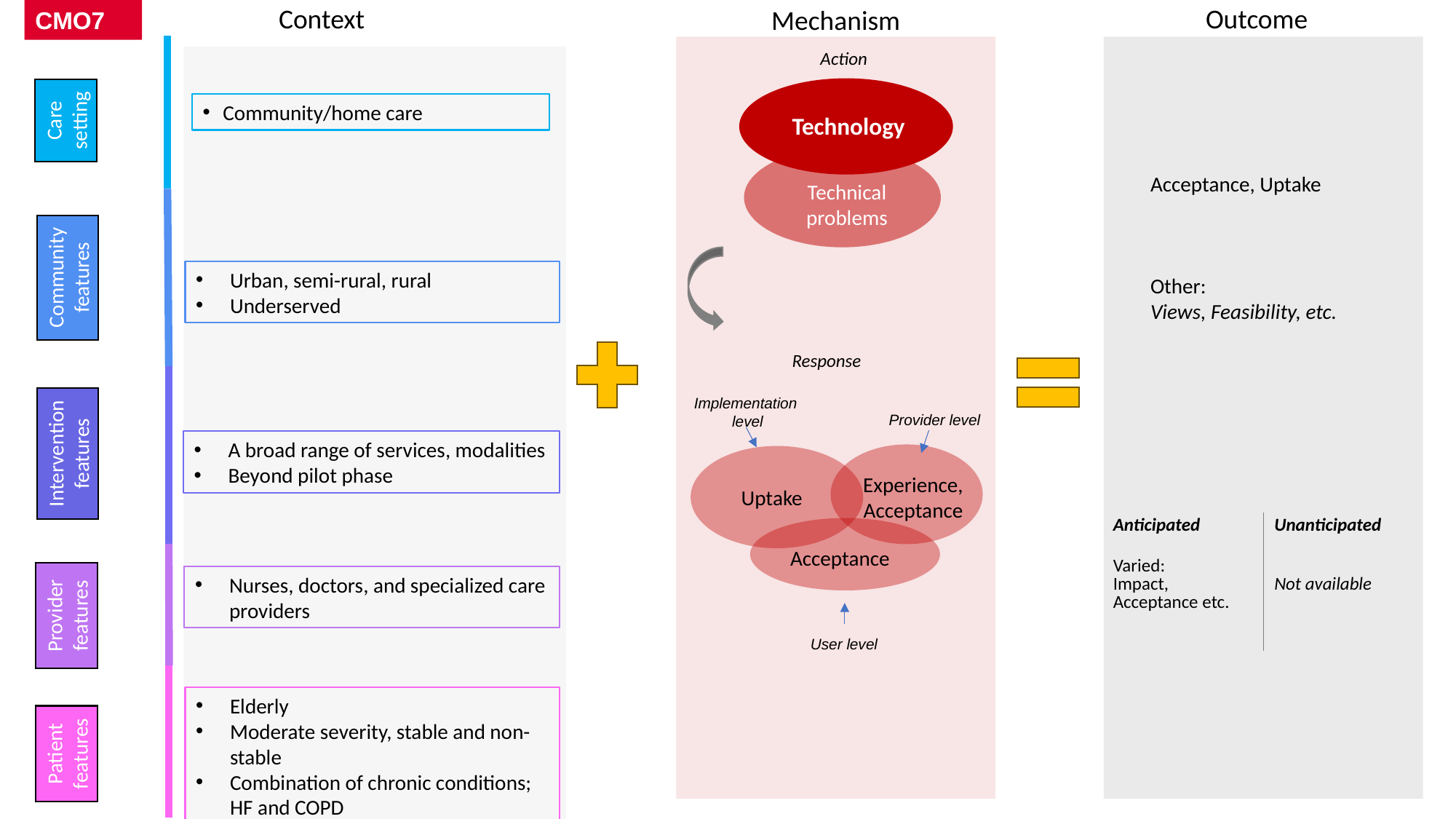

CMO7
Context
Outcome
Mechanism
Action
Care setting
Community/home care
Technology
Acceptance, Uptake
Other:
Views, Feasibility, etc.
Technical problems
Community features
Urban, semi-rural, rural
Underserved
Response
Implementation level
Provider level
Intervention features
A broad range of services, modalities
Beyond pilot phase
Experience, Acceptance
Uptake
| Anticipated | Unanticipated |
| --- | --- |
| Varied: Impact, Acceptance etc. | Not available |
Acceptance
Nurses, doctors, and specialized care providers
Provider features
User level
Elderly
Moderate severity, stable and non-stable
Combination of chronic conditions; HF and COPD
Patient features

## Slide 8
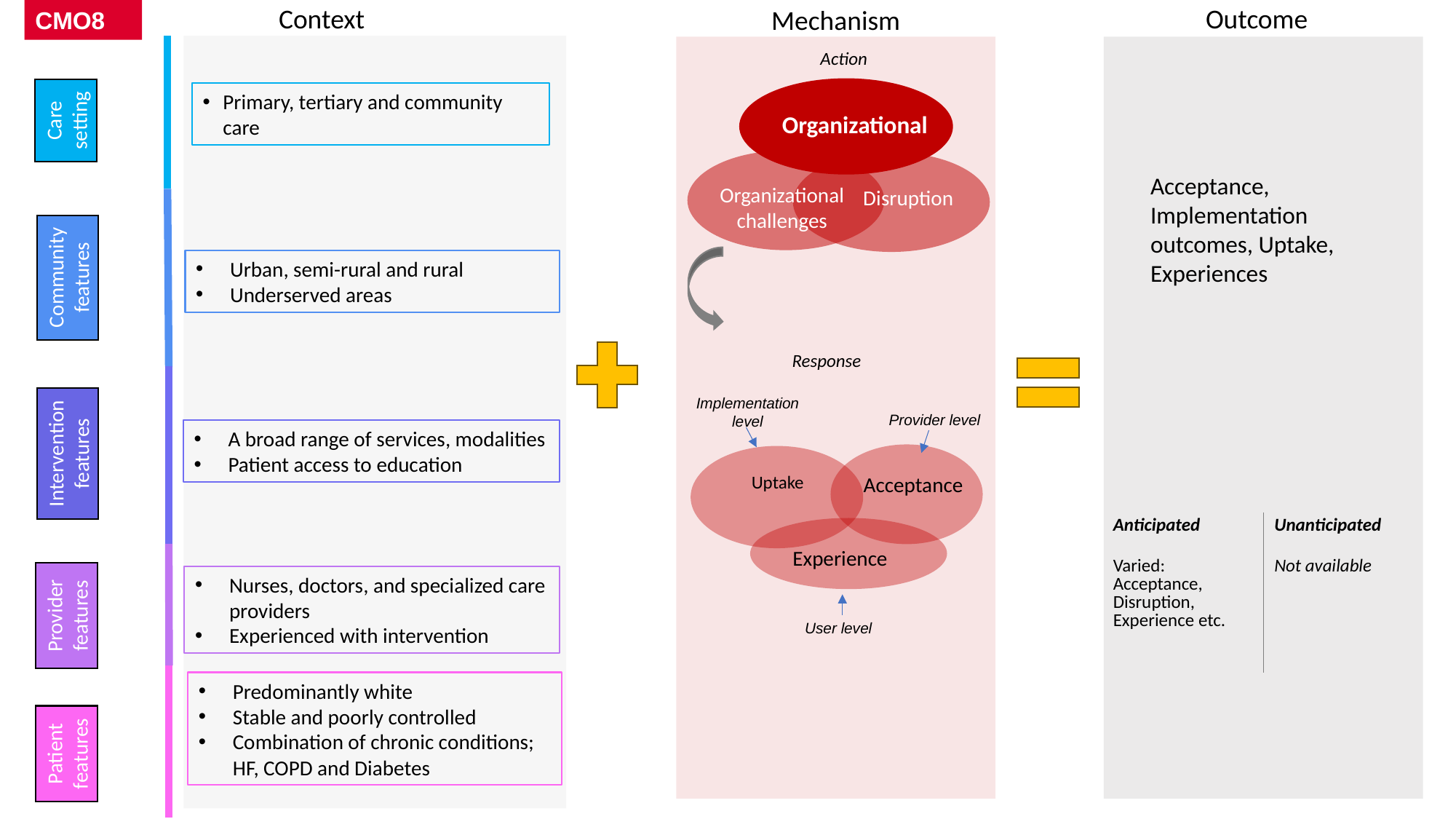

CMO8
Context
Outcome
Mechanism
Action
Primary, tertiary and community care
Care setting
Organizational
Acceptance, Implementation outcomes, Uptake, Experiences
Organizational challenges
Disruption
Community features
Urban, semi-rural and rural
Underserved areas
Response
Implementation level
Provider level
A broad range of services, modalities
Patient access to education
Intervention features
Uptake
Acceptance
| Anticipated | Unanticipated |
| --- | --- |
| Varied: Acceptance, Disruption, Experience etc. | Not available |
Experience
Nurses, doctors, and specialized care providers
Experienced with intervention
Provider features
User level
Predominantly white
Stable and poorly controlled
Combination of chronic conditions; HF, COPD and Diabetes
Patient features

## Slide 9
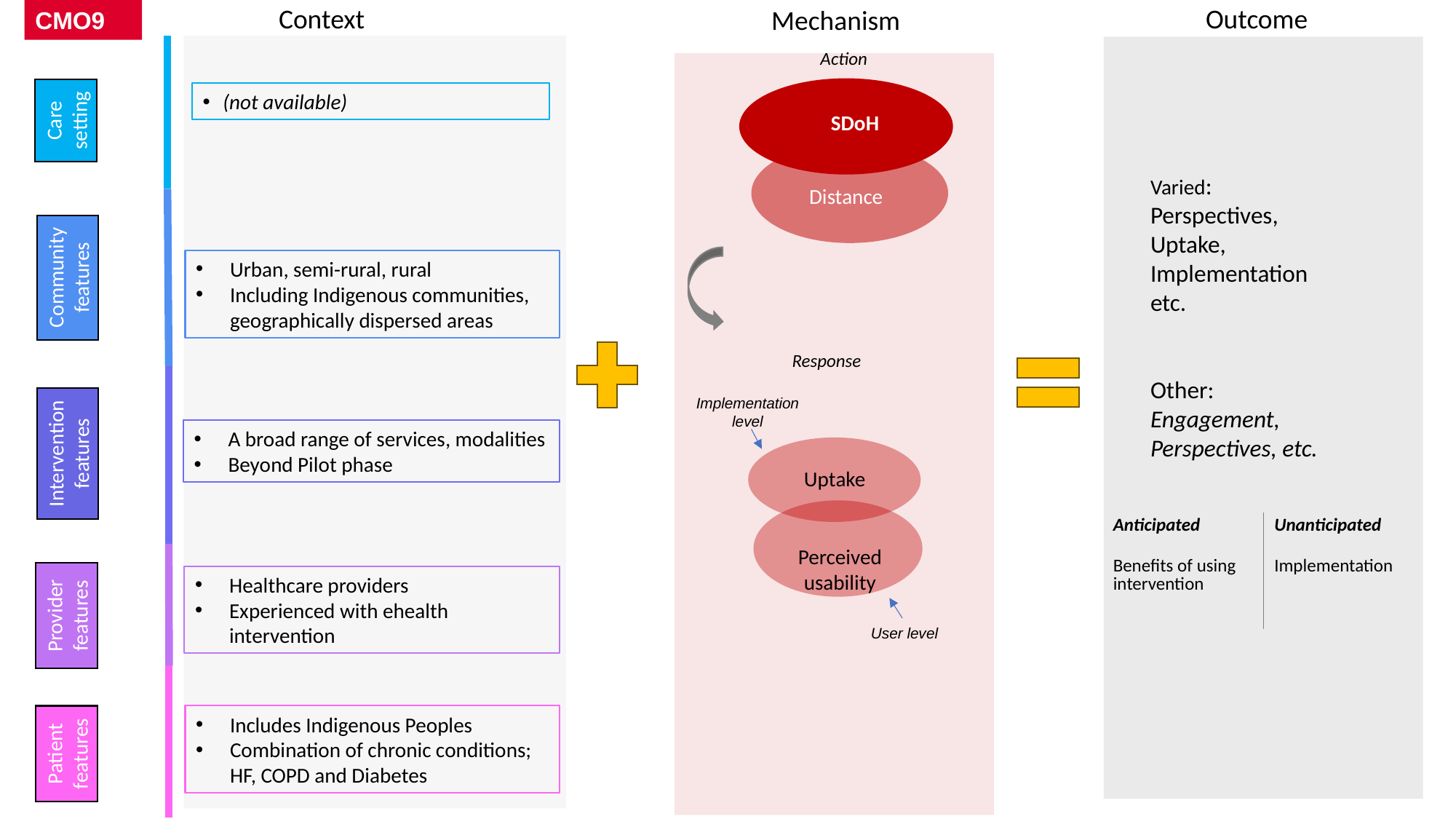

CMO9
Context
Outcome
Mechanism
Action
(not available)
Care setting
SDoH
Varied: Perspectives, Uptake, Implementation etc.
Other:
Engagement, Perspectives, etc.
Distance
Community features
Urban, semi-rural, rural
Including Indigenous communities, geographically dispersed areas
Response
Implementation level
A broad range of services, modalities
Beyond Pilot phase
Intervention features
Uptake
| Anticipated | Unanticipated |
| --- | --- |
| Benefits of using intervention | Implementation |
Perceived usability
Healthcare providers
Experienced with ehealth intervention
Provider features
User level
Includes Indigenous Peoples
Combination of chronic conditions; HF, COPD and Diabetes
Patient features

## Slide 10
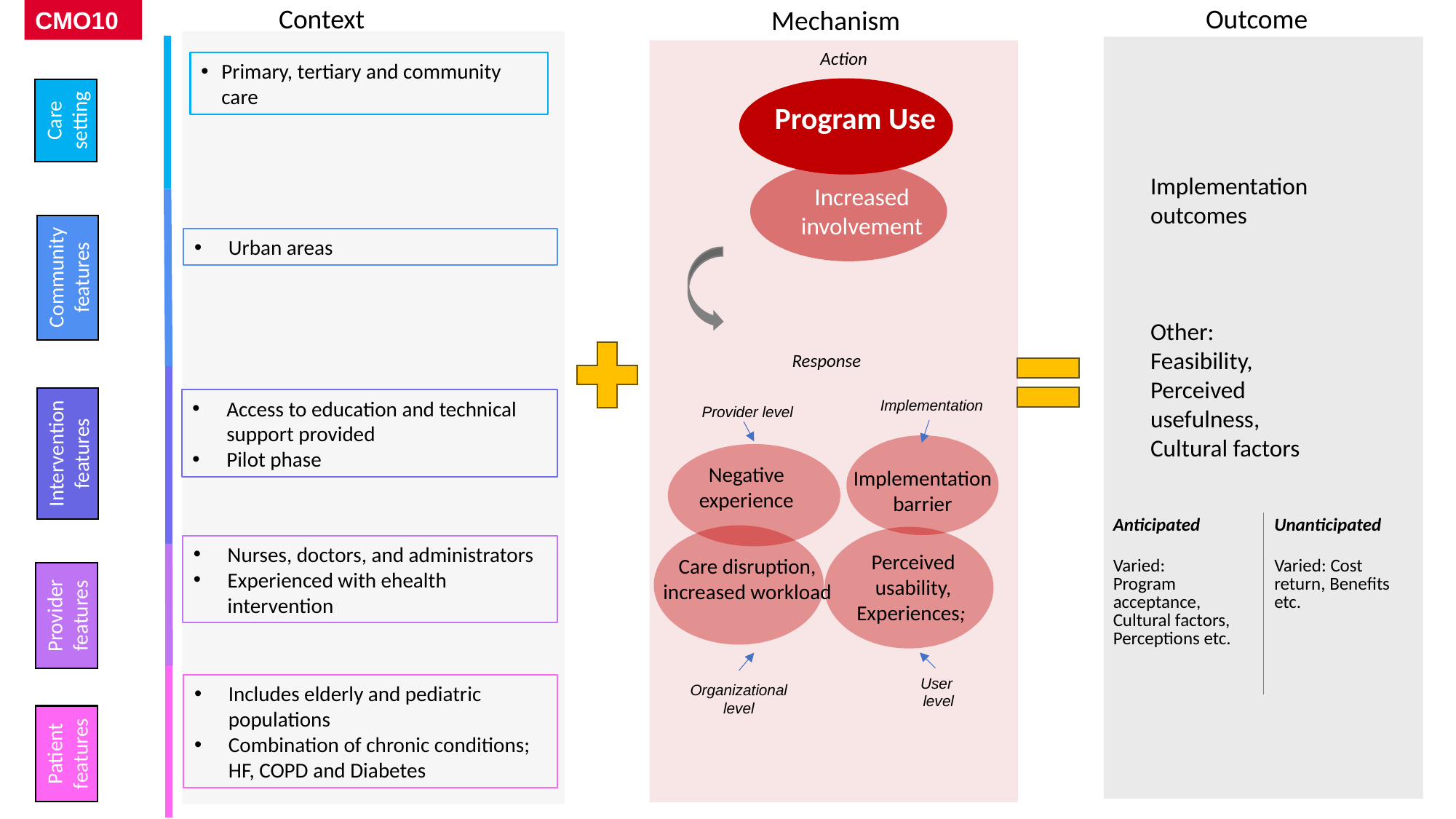

CMO10
Context
Outcome
Mechanism
Action
Primary, tertiary and community care
Care setting
Program Use
Implementation outcomes
Other:
Feasibility, Perceived usefulness, Cultural factors
Increased involvement
Urban areas
Community features
Response
Access to education and technical support provided
Pilot phase
Implementation
Provider level
Intervention features
Negative experience
Implementation barrier
| Anticipated | Unanticipated |
| --- | --- |
| Varied: Program acceptance, Cultural factors, Perceptions etc. | Varied: Cost return, Benefits etc. |
Nurses, doctors, and administrators
Experienced with ehealth intervention
Perceived usability, Experiences;
Care disruption, increased workload
Provider features
User
level
Includes elderly and pediatric populations
Combination of chronic conditions; HF, COPD and Diabetes
Organizational level
Patient features
